# The optimal threshold of PD-L1 combined positive score to predict the benefit of PD-1 antibody plus chemotherapy for patients with HER2-negative gastric adenocarcinoma: a meta-analysis

**DOI:** 10.1007/s00262-024-03726-1

**Published:** 2024-05-16

**Authors:** Ji-Bin Li, Ming-Yu Lai, Zhuo-Chen Lin, Wen-Long Guan, Yu-Ting Sun, Jing Yang, Wen-Xuan Wang, Zhi-Rong Yang, Miao-Zhen Qiu

**Affiliations:** 1 Department of Clinical Research, State Key Laboratory of Oncology in South China, Collaborative Innovation Center for Cancer Medicine, Sun Yat-Sen University Cancer Center, 651 Dongfeng Road East, Guangzhou, 510060 People’s Republic of China; 2 Department of Medical Oncology, State Key Laboratory of Oncology in South China, Collaborative Innovation Center for Cancer Medicine, Sun Yat-Sen University Cancer Center, 651 Dongfeng Road East, Guangzhou, 510060 People’s Republic of China; 3 Department of Medical Records, The First Affiliated Hospital, Sun Yat‑Sen University, Guangzhou, People’s Republic of China; 4 School of Public Health, Sun Yat-Sen University, Guangzhou, People’s Republic of China; 5 Center for Biomedical Information Technology, Shenzhen Institute of Advanced Technology, Chinese Academy of Sciences, Shenzhen, 518055 People’s Republic of China

**Keywords:** Gastric cancer, Immune checkpoint inhibitors, Combined positive score, Meta-analysis, Cutoff value

## Abstract

**Background:**

Immune checkpoint inhibitors (ICIs) combined with chemotherapy have become the first-line treatment of metastatic gastric and gastroesophageal adenocarcinomas (GEACs). This study aims to figure out the optimal combined positive score (CPS) cutoff value.

**Methods:**

We searched for randomized phase III trials to investigate the efficacy of ICIs plus chemotherapy for metastatic GEACs compared with chemotherapy alone. Pooled analyses of hazard ratios (HRs) based on PD-L1 expression were performed.

**Results:**

A total of six trials (KEYNOTE-062, KEYNOTE-590, KEYNOTE-859, ATTRACTION-04, CheckMate 649, and ORIENT-16) were included, comprising 5,242 patients. ICIs plus chemotherapy significantly improved OS (HR: 0.79, 95% CI 0.72–0.86 in global patients; HR: 0.75, 95% CI 0.57–0.98 in Asian patients) and PFS (HR: 0.74, 95% CI 0.68–0.82 in global patients; HR: 0.64, 95% CI 0.56–0.73 in Asian patients) compared with chemotherapy alone. The differences in OS (ratio of HR: 1.05, 95% CI 0.79–1.40; predictive value: − 5.1%) and PFS (ratio of HR: 1.16, 95% CI 0.98–1.36; predictive value: − 13.5%) were not statistically significant between the global and Asian patients. Subgroup analyses indicated that the optimal CPS threshold was at ≥ 5 for OS and ≥ 10 for PFS with the highest predictive values.

**Conclusions:**

The benefit derived from ICIs plus chemotherapy is similar between Asian and global GEAC patients. However, those with a PD-L1 CPS < 5 or CPS < 10 may not have significant benefits from ICIs therapy. Therefore, it is advisable to routinely assess PD-L1 expression in GEAC patients considered for ICIs treatment.

**Supplementary Information:**

The online version contains supplementary material available at 10.1007/s00262-024-03726-1.

## Introduction

Several randomized phase III clinical trials (RCTs) reported a significant improvement in objective response rate (ORR) with immune checkpoint inhibitors (ICIs) combined with chemotherapy, compared with doublet chemotherapy, as the first-line treatment for metastatic gastric and gastroesophageal adenocarcinomas (GEACs) [1–4]. However, the improvement of overall survival (OS) and progression-free survival (PFS) is inconclusive [2, 3, 5]. CheckMate 649 and ORIENT-16 showed significant improvements in PFS and OS in both PD-L1 combined positive score (CPS) > 5 patients and the whole population cohort [1, 4]. Based on these two studies, nivolumab was approved by the U.S. Food and Drug Administration and Sintilimab by the Chinese National Medical Products Administration as first-line therapies regardless of PD-L1 status for GEACs.

In the RATIONALE-305 trial, tislelizumab plus chemotherapy demonstrated statistically significant improvement in OS, PFS, and ORR compared to placebo plus chemotherapy in patients with PD-L1-positive gastric cancer (GC)/gastroesophageal junction cancer (GEJC) [6]. A recent secondary analysis of individual patient data indicates the lack of benefit in adding ICIs to chemotherapy in gastric adenocarcinoma patients with low PD-L1 expression [7]. Therefore, the efficacy of ICIs plus chemotherapy for patients with low PD-L1 expression remains uncertain. To optimize the use of ICIs, determining the optimal cutoff value of CPS to guide ICIs treatment is necessary. Moreover, previous studies indicated that Asian patients might benefit more from ICIs than those from other countries worldwide [8–10]. With the release of new survival data in the Asian population, it becomes possible to compare the survival benefit between Asian patients and the global population.

In this study, we conducted a series of pooled analyses to clarify the survival benefit in the subgroup of different PD-L1 expression patients with gastric adenocarcinoma, and further assessed the probably optimal cutoff value of PD-L1 CPS that patients could significantly benefit from adding ICIs treatment to chemotherapy, stratified by Asian and global patients.

## Methods

### Study selection and eligibility criteria

A comprehensive literature search was conducted on PubMed, Embase, and Cochrane databases from January 2015 to February 2023 using key terms of gastric adenocarcinoma, immunotherapy, and RCTs and their derivation. Considering that human epithelial growth factor receptor 2 (HER2)-positive patients have different treatment approaches, we focused only on HER2-negative patients in this study. The full search strategy is shown in supplemental Table S1. We also searched the abstracts from annual conferences of the American Society of Clinical Oncology, the European Society of Medical Oncology, and the American Association for Cancer Research. If multiple publications of the same trial were identified, the latest and most comprehensive publication was selected. After removing duplications, two investigators (JBL and MYL) independently reviewed and screened the publications. Any discrepancies were resolved by a panel discussion. All relevant publications and their supplemental materials were thoroughly assessed.

Phase III RCTs that investigated the efficacy of ICIs plus chemotherapy compared to chemotherapy among HER2-negative patients with unresectable or metastatic GEACs were considered for this study. Additional criteria for the clinical studies included: the articles were written in English, the patients were adults, first-line setting, histological diagnosis of adenocarcinoma, and PD-L1 status was evaluated by CPS or tumor cell proportion score (TPS) method. This study was conducted in accordance with the Preferred Reporting Items for Systematic Reviews and Meta-analyses (PRISMA) reporting guideline (Supplemental Table S2) [11].

### Data extraction and quality assessment

Two investigators (JBL and MYL) independently extracted the data, and any disagreements were resolved through a panel discussion. The extracted data include the study title, publication year, clinical trial identification number, country of origin, immunotherapy drugs, number of patients, and PD-L1 expression characteristics (i.e., threshold, type of PD-L1 antibody clone, and immunohistochemical scoring method of PD-L1). The main extracted outcomes were OS and PFS based on the PD-L1 expression. The hazard ratios (HRs) with 95% confidence intervals (CIs) of OS and PFS were also retrieved. In cases where HRs with 95% CIs for specific PD-L1 expression subgroups were not reported, the estimates were obtained using reconstructed individual patient survival data (IPD) (Supplemental methods).

The risk of bias of the trials was independently assessed by two investigators (JBL and MYL) using the Cochrane Risk of Bias (RoB 2) tool for RCTs [12]. Our preliminary assessment indicated that the methodological quality of the included trials was rated as having a low risk of bias (Supplemental Table S3).

### Statistical analysis

The pooled HRs and corresponding 95% CIs are summarized using the generic inverse variance method. The heterogeneity across studies was assessed using the *I*^2^ statistics [13]. As recommended in the previous literature [14, 15], the fixed-effect meta-analysis was performed when *I*^2^ = 0. Conversely, the random-effect model was performed when *I*^2^ > 0 to account for heterogeneity, using the restricted maximum likelihood method for *τ*^2^. The Hartung–Knapp correction was applied to properly analyze the outcomes generated by a few articles [15], as this method has been shown to substantially outperform the DerSimonian–Laird method [16]. The pre-specified subgroup analyses were conducted between global and Asian patients and between those with high and low PD-L1 expression at specific CPS thresholds (i.e., ≥ 1 vs. < 1, ≥ 5 vs. < 5, and ≥ 10 vs. < 10). Sensitivity analyses were performed by excluding ORIENT-16 and ATTRACTION-04 from global patients because both trials only involved Asian patients. The sensitivity was also done using the Bayesian random-effect method to account for the limited number of included studies [17]. Publication bias was investigated using funnel plots and Egger’s test.

We computed the ratios of HRs at various CPS thresholds, whereby a ratio of 1.00 indicates that the HR of the high and low PD-L1 expression subgroups (or global and Asian patients) is equal in magnitude, and ratios greater than or less than 1.00 indicates that the efficacy of ICIs treatment was better in low (Asian) or high PD-L1 (global) group [18, 19]. We also calculated the predictive value between low and high PD-L1 expression subgroups at specific CPS thresholds (or global and Asian patients), which was defined as the ratio of HRs across two groups, with a higher percentage indicating a more favorable outcome for the high PD-L1 expression group or Asian patients [20].

The cumulative survival probabilities were estimated using Kaplan–Meier curves based on the reconstruction IPD data, and the differences were compared using the log-rank test. HRs with their corresponding 95% CIs were calculated using the unadjusted Cox proportional hazards model. All analyses were performed in *R* (version 4.2.2) using the survival, ggplot2, KMSubtraction, IPDfromKM, and metafor packages. A two-sided *p* < 0.05 was considered statistically significant.

## Results

### Baseline characteristics

Six articles (ATTRACTION-04 [3], CheckMate 649 [1, 21], KEYNOTE-062 [2, 22], and KEYNOTE-590 [23]) and two conference abstracts (ORIENT-16 [4] and KEYNOTE-859 [5]) were involved (Supplemental Figure S1). A total of 5242 patients were, with 2624 (50.1%) in ICIs plus chemotherapy and 2618 (49.9%) in chemotherapy groups, involved. CheckMate 649, KEYNOTE-062, KEYNOTE-590, and KEYNOTE-859 enrolled patients from various countries (referred to as global patients in this study), while ATTRACTION-04 and ORIENT-16 enrolled only Asian patients. Moreover, CheckMate 649 and KEYNOTE-062 reported subgroup outcomes for Asian patients. Five trials, including ATTRACTION-04, CheckMate 649, ORIENT-16, KEYNOTE-590, and KEYNOTE-859, enrolled patients regardless of PD-L1 expression status, while KEYNOTE-062 enrolled patients with a CPS ≥ 1. KEYNOTE-590 enrolled patients with ESCC and oesophageal/gastroesophageal junction adenocarcinoma, and only data on GEJ adenocarcinoma were included in this study (Table 1).

**Table 1:** Study information and characteristics of trials

Study name, year	Patients	Histology	PD-L1 detection assays	CPS of patients	Treatment arms	Sample size				Median OS	Median PFS
						All	CPS ≥ 1	CPS ≥ 5	CPS ≥ 10		
CheckMate 649, 2021	Global	Previously untreated, unresectable advanced or metastatic gastric, gastroesophageal junction, or esophageal adenocarcinoma	28–8 assay	Regardless of PD-L1 expression	Nivolumab plus chemotherapy	789	641	473	–	13.8	7.7
					Chemotherapy	792	655	482	–	11.6	6.9
CheckMate 649, 2022	Asian		28–8 assay	Regardless of PD-L1 expression	Nivolumab plus chemotherapy	99	89	75	–	14.3	8.3
					Chemotherapy	109	94	81	–	10.3	5.6
KEYNOTE-062, 2020	Global	Advanced gastric/gastroesophageal junction (G/GEJ) cancer	22C3 assay	CPS ≥ 1	Pembrolizumab plus chemotherapy	257	257	–	99	12.5	6.9
					Chemotherapy	250	250	–	90	11.1	6.4
KEYNOTE-062, 2022	Asian		22C3 assay	CPS ≥ 1	Pembrolizumab plus chemotherapy	64	64	–	26	16.5	8.5
					Chemotherapy	61	61	–	22	13.8	6.5
ATTRACTION-04, 2022	Asian	HER2-negative, unresectable advanced or recurrent gastric or gastroesophageal junction cancer	28–8 assay	Regardless of PD-L1 expression	Nivolumab plus chemotherapy	362	–	–	–	17.5	10.5
					Chemotherapy	362	–	–	–	17.2	8.3
ORIENT-16, 2021	Asian	Untreated, unresectable, locally advanced, recurrent or metastatic G/GEJ adenocarcinoma, regardless of PD-L1 expression	22C3 assay	Regardless of PD-L1 expression	Sintilimab plus chemotherapy	327	275	197	146	15.2	7.1
					Chemotherapy	323	271	200	142	12.3	5.7
KEYNOTE-590, 2021	Global	Siewert type 1 gastroesophageal junction cancer	22C3 assay	Regardless of PD-L1 expression	Pembrolizumab plus chemotherapy	99	–	–	43	11.6	6.3
					Chemotherapy	102	–	–	54	9.9	5.7
KEYNOTE-859, 2023	Global	G/GEJ adenocarcinoma, locally advanced unresectable or metastatic disease, no prior treatment, HER2-negative	22C3 assay	Regardless of PD-L1 expression	Pembrolizumab plus chemotherapy	790	618	–	279	12.9	6.9
					Chemotherapy	789	617	–	272	11.5	5.6

G/EGJ, gastric/esophageal–gastric junction; CPS, combined positive score; OS, overall survival; PFS, progression-free survival; PD-L1, programmed cell death-ligand 1; HER2, human epidermal growth factor receptor 2

### Overall survival

**Fig. 1:**
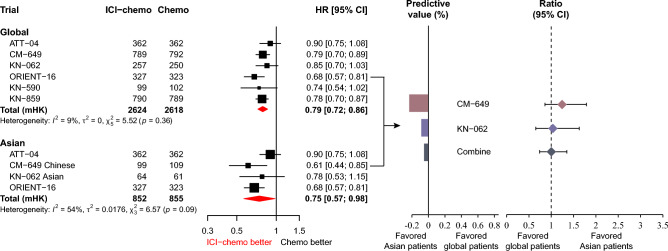
Forest plot for overall survival comparing ICIs plus chemotherapy with chemotherapy alone in global and Asian patients.${\text{Predictive value = }}\frac{{\text{HR in Asian - HR in global}}}{{\text{HR in global}}} \times 100\%$

ICIs plus chemotherapy led to a significantly improved OS in the global (pooled HR: 0.79, 95% CI 0.72–0.86) and Asian patients (pooled HR: 0.75, 95% CI 0.57–0.98) compared to chemotherapy alone (Fig. 1). The predictive value between global and Asian patients was – 5.1%, and the ratio of HR was 1.05 (95% CI 0.79–1.40, *p* = 1.0), indicating that the magnitude of benefit from ICIs plus chemotherapy is comparable between global and Asian patients. The KM curves based on the reconstructed IPD showed consistent results (Supplemental Figure S2).

**Fig. 2:**
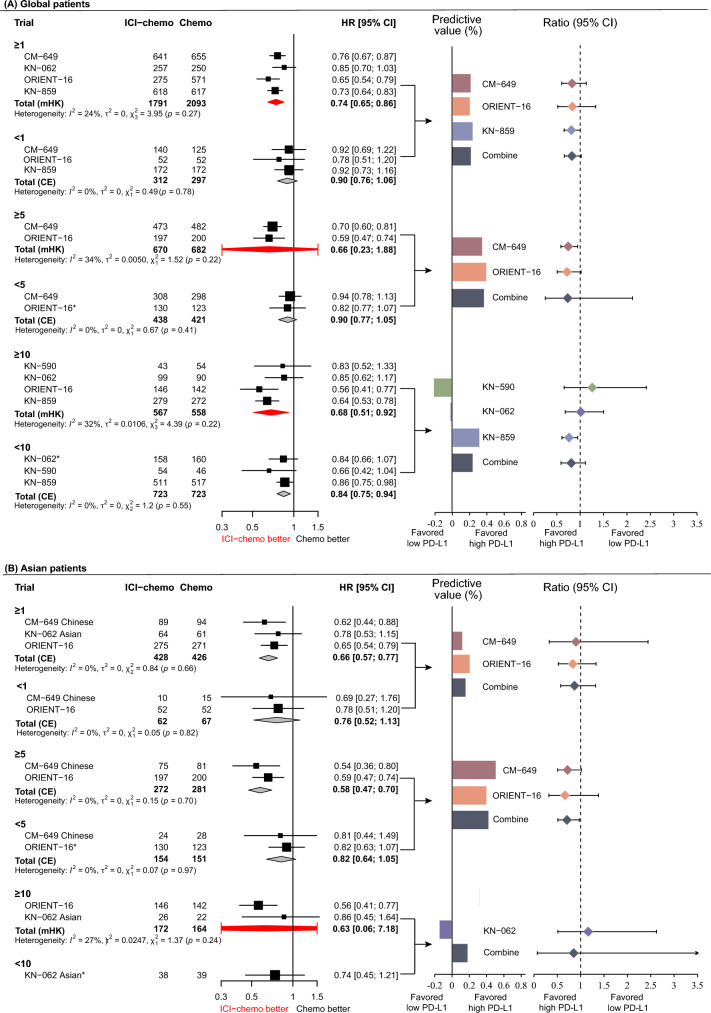
Forest plot for the overall survival of ICIs plus chemotherapy versus chemotherapy alone stratified by CPS thresholds. **A** Global patients and **B** Asian patients. *HRs and 95%CIs were derived from the reconstruction IPD data.${\text{Predictive value = }}\frac{{\text{HR in low PD - L1 group - HR in high PD - L1 group}}}{{\text{HR in high PD - L1 group}}} \times 100\%$

**Fig. 3:**
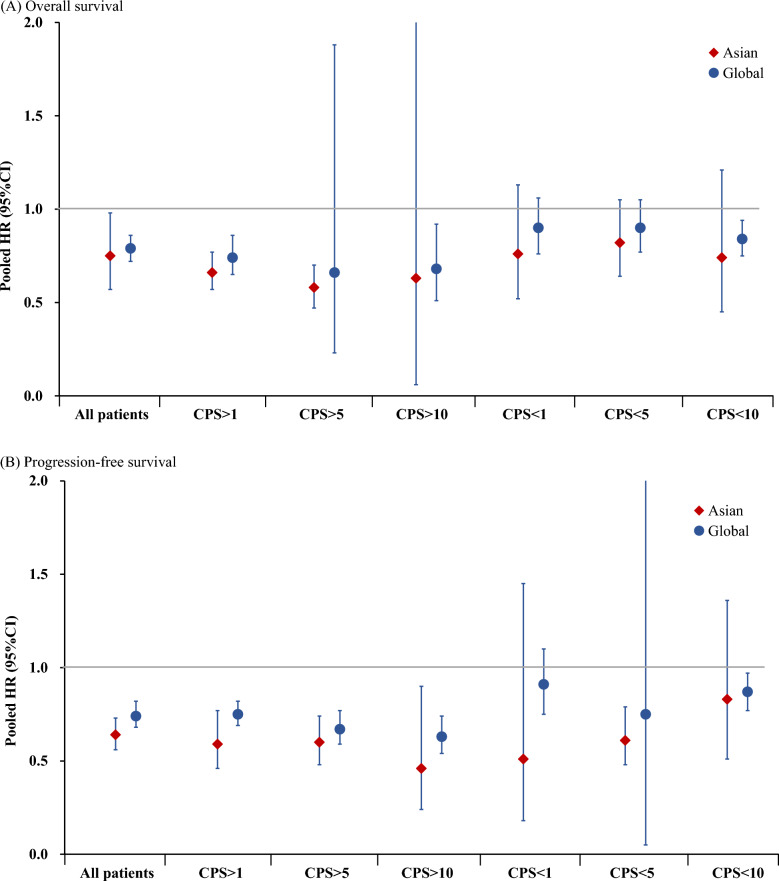
The efficacy of ICIs plus chemotherapy on OS and PFS according to CPS thresholds

In the global patients, ICIs plus chemotherapy showed a significantly improved OS for patients with CPS ≥ 1 (pooled HR: 0.74, 95% CI 0.65–0.86) and CPS ≥ 10 (pooled HR: 0.68, 95% CI 0.51–0.92) compared to chemotherapy alone. However, the differences in OS between ICIs plus chemotherapy and chemotherapy alone were statistically non-significant in the CPS < 1 subgroup (HR = 0.90, 95% CI 0.76–1.06) and CPS < 5 (HR = 0.90, 95% CI 0.77–1.05), while the difference in OS between ICIs plus chemotherapy and chemotherapy alone in the CPS < 10 subgroup was statistically significant (HR: 0.84, 95% CI 0.75–0.94) (Fig. 2A and Fig. 3A). Regarding the different CPS subgroups, the ratios of HRs (95% CIs) were 0.82 (95% CI 0.66–1.02, *p* = 0.078) between CPS ≥ 1 and < 1, 0.73 (95% CI 0.25–2.12, *p* = 0.567) between CPS ≥ 5 and < 5, and 0.81 (95% CI 0.59–1.11, *p* = 0.189) between CPS ≥ 10 and < 10, respectively. The corresponding predictive values were 21.6%, 33.4%, and 23.5% for CPS thresholds of ≥ 1, ≥ 5, and ≥ 10 (Fig. 2A). These results suggested that the potential optimal threshold of CPS appeared at ≥ 5 with the highest predictive value.

In Asian patients, the HR (95% CI) for OS between ICIs plus chemotherapy and chemotherapy alone was 0.66 (95% CI 0.57–0.77) for CPS ≥ 1, 0.58 (95% CI 0.47–0.70) for CPS ≥ 5, and 0.63 (95% CI 0.06–7.18) for CPS ≥ 10, respectively. The differences in OS between ICIs plus chemotherapy and chemotherapy alone for CPS < 1, CPS < 5, and CPS < 10 subgroups were statistically non-significant (Fig. 2B and Fig. 3A). The ratios with 95% CIs of HRs between CPS ≥ 1 and < 1, between CPS ≥ 5 and < 5, and between CPS ≥ 10 and < 10 were 0.87 (95% CI 0.57–1.32, *p* = 0.506), 0.71 (95% CI 0.51–0.97, *p* = 0.033), and 0.85 (95% CI 0.07–9.80, *p* = 0.897), and the predictive values of CPS thresholds at ≥ 1, ≥ 5, and ≥ 10 were 15.2%, 40.4%, and 17.5%, respectively (Fig. 2B). These results indicated that the optimal threshold of CPS where patients would significantly benefit from adding ICIs therapy is CPS ≥ 5.

### Progression-free survival

**Fig. 4:**
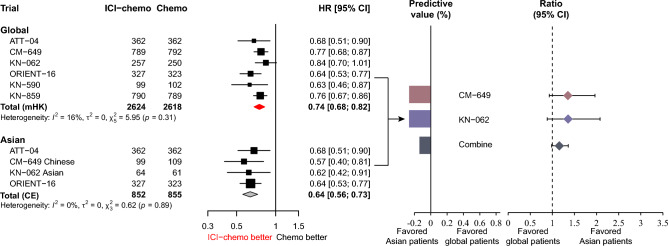
Forest plot for progression-free survival comparing ICIs plus chemotherapy with chemotherapy alone in global and Asian patients. ${\text{Predictive value = }}\frac{{\text{HR in Asian - HR in global}}}{{\text{HR in global}}} \times 100\%$

The pooled results showed a significant improvement in PFS with ICIs plus chemotherapy in the global (pooled HR: 0.74, 95% CI 0.68–0.82) and Asian patients (pooled HR: 0.64, 95% CI 0.56–0.73). The ratio of HRs between global and Asian patients was 1.16 (95% CI 0.98–1.36, *p* = 1.0), and the predictive value was − 13.5% (Fig. 4). The KM curves based on the reconstructed IPD showed consistent results (Supplemental Figure S3).

**Fig. 5:**
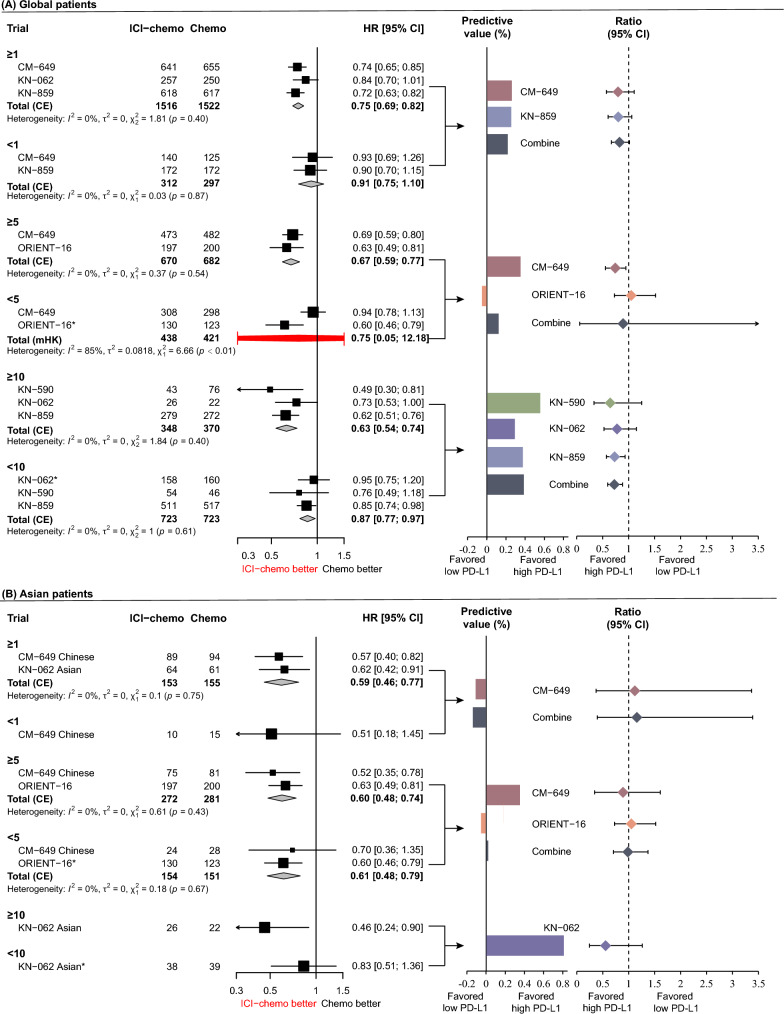
Forest plot for progression-free survival of ICIs plus chemotherapy versus chemotherapy alone stratified by CPS thresholds. **A** Global patients and **B** Asian patients. *HR and 95%CI derived from the reconstruction. ${\text{Predictive value = }}\frac{{\text{HR in low PD - L1 group - HR in high PD - L1 group}}}{{\text{HR in high PD - L1 group}}} \times 100\%$

In the global patients, the pooled results indicated that ICIs plus chemotherapy significantly improved PFS in the CPS ≥ 1 (pooled HR: 0.75, 95% CI 0.69–0.82), CPS ≥ 5 (pooled HR: 0.67, 95% CI 0.59–0.77), and CPS ≥ 10 (pooled HR: 0.63, 95% CI 0.54–0.74) subgroups compared to chemotherapy alone. In the CPS < 1 and CPS < 5 groups, there was no significant improvement in PFS between ICIs plus chemotherapy and chemotherapy, while improvement in PFS for CPS < 10 with ICIs plus chemotherapy was significantly greater than that with chemotherapy alone (HR: 0.87, 95% CI 0.77–0.97) (Fig. 5A and Fig. 3B). The predictive values at CPS thresholds ≥ 1, ≥ 5, and ≥ 10 were 21.3%, 11.9%, and 38.1%, respectively, and the corresponding ratios of HRs (95% CI) between high and low CPS thresholds were 0.82 (95% CI 0.67–1.02, *p* = 0.071), 0.89 (95% CI 0.06–13.99, *p* = 0.936), and 0.72 (95% CI 0.60–0.88, *p* = 0.001), respectively (Fig. 5A). These results suggest that a CPS ≥ 10 is the optimal CPS threshold at which patients would significantly benefit from ICIs therapy in terms of PFS.

In Asian patients, the HRs (95% CI) between ICIs plus chemotherapy and chemotherapy in the CPS ≥ 1, CPS ≥ 5, and CPS ≥ 10 groups were 0.59 (95% CI 0.46–0.77), 0.60 (95% CI 0.48–0.74), and 0.46 (95% CI 0.24–0.90), respectively. The HRs (95% CI) in the CPS < 1, < 5, and < 10 were 0.51 (95% CI 0.18–1.45), 0.61 (95% CI 0.48–0.79), and 0.83 (95% CI 0.51–1.36), respectively. Although the optimal predictive CPS threshold appeared at ≥ 10, with a predictive value of 80.4%, the ratio (95% CI) of HR between CPS ≥ 10 and < 10 was non-significant (ratio: 0.55, 95% CI 0.24–1.26, *p* = 0.160) (Fig. 5B and Fig. 3B).

The sensitivity analyses showed similar results to the main findings by using the Bayesian random-effect method (Supplemental Table S9) or excluding ORIENT-16 and ATTRACTION-04 from the global analysis (Table S10). The funnel plots and Egger’s test show no clear evidence of publication bias (Supplemental Figure S4).

## Discussion

The treatment of metastatic GEACs remains a significant challenge, with conflicting results in various RCTs regarding the efficacy of ICIs plus chemotherapy in the first-line setting. In this study, we comprehensively compared the efficacy of ICIs plus chemotherapy with chemotherapy in metastatic GEACs, indicating that the ICIs plus chemotherapy significantly improved both OS and PFS in global and Asian patients.

The relationship between race and the efficacy of ICIs remains inconclusive. Some studies have suggested that Asian GC patients may benefit more from ICIs than non-Asian patients [8–10]. Asian and non-Asian GCs have distinct tumor immunity signatures associated with T cell function according to gene expression profile analysis, which may contribute to differences in clinical outcome [24]. In addition, differences in gene expression related to T cell function and clinicopathological features could potentially explain the greater benefits observed in Asian patients with non-small cell lung cancer [25]. However, a meta-analysis from Goldvaser et al. [26] found that Asian subgroups have no increased benefit from ICIs. In line with the finding, our study indicates that the magnitude of efficacy from ICIs was similar between global and Asian patients, suggesting that the impact of race on survival benefits from ICIs was small, at least for gastric adenocarcinoma.

The optimal threshold of PD-L1 CPS where patients would considerably benefit from ICIs treatment remains controversial, despite the positive correlation between PD-L1 expression and ICI efficacy. For instance, Peng et al. [27] reported that subgroup analysis based on PD-L1 expression showed the appropriate cutoff value for CPS was > 1 for ICI monotherapy in GC. However, a secondary analysis of IPD indicated a lack of benefit in adding ICIs to chemotherapy in gastric adenocarcinoma patients with low PD-L1 expression [7]. But this study only included data from CheckMate 649. Though several new phase III RCTs were released, few RCTs have reported data on subgroups of patients with low PD-L1 expression, making it difficult to assess the survival outcomes in this specific population. In this study, we applied two methods to exploratorily determine the optimal threshold value of CPS for guiding ICIs therapy. The results suggest the optimal CPS thresholds of ≥ 5 for OS and ≥ 10 for PFS with the highest predictive values. The well-documented threshold value could help physicians to better delineate target patients who might considerably benefit from ICIs treatment. Our results also imply that the all-population-wide significant benefit from ICIs in clinical studies may be due to the high PD-L1 expression subpopulation. Thus, it is advisable to assess PD-L1 expression before treatment with ICIs in combination with chemotherapy for HER2-negative GEACs. However, given that some ratios of HR were not statistically significant, further studies are highly warranted to verify our findings.

It should be emphasized that, in addition to PD-L1 expression, other biomarkers, such as tumor mutational burden (TMB), Epstein–Barr virus-encoded RNA (EBER) status, and microsatellite instability (MSI) status, might have the potential to predict the response to ICIs. TMB serves as a predictive biomarker for immunotherapy response across various cancer types, indicating a higher likelihood of clinical benefit in patients with elevated TMB [28]. EBER-positive tumors are heavily infiltrated by immune cells, making EBER-positive GC more sensitive to chemotherapy plus ICIs [29]. MSI-high tumors harbor a high number of genetic alterations, leading to increased neoantigen load and immune recognition [30]. Given that a single biomarker may not capture the full complexity of tumor-immune interactions, a multifactorial approach by integrating multiple biomarkers might provide a comprehensive evaluation of patients’ potential responses to ICIs in future research.

The discrepancy between clinical trials affecting CPS could be partially related to the different detection assays. One significant reason for the impact of diverse PD-L1 detection methods on CPS values is the lack of standardized guidelines and harmonization in PD-L1 testing. Without uniform protocols, there is a risk of subjectivity and variability in the interpretation of PD-L1 expression levels, leading to inconsistencies in CPS values and, ultimately, the analysis results. Raghav Sundar et al*.* found the 28–8 assay yielded a much higher percentage of PD-L1-positive samples than the 22C3 assay, especially at CPS ≥ 5. However, we found the proportions of people with CPS ≥ 5 in the CheckMate 649 study (60.4%, using the 28–8 assay) and ORIENT-16 (60.1%, using the 22C3 assay) were consistent. Kim et al. [32] found that PD-L1 22C3 and 28–8 pharmDx assays were highly comparable at different CPS cutoffs in GC, which support our findings. In our analysis, the included trials utilized different PD-L1 assays. Therefore, it is important to further explore the impact of different PD-L1 assays on the observed survival benefits, and establishing consensus guidelines for PD-L1 detection and scoring criteria would enhance the comparability of CPS values and facilitate more research to understand the implications of using different PD-L1 assays in assessing treatment outcomes in GEACs.

However, there are several limitations to this study. First, the potential publication bias could not be ruled out although the test indicates no such evidence. Second, although we strictly restricted the included trials and applied appropriate statistical strategies to control heterogeneities, the influence of differences in patients, types of ICIs, and methods for assessing PD-L1 expression between trials should be warranted in explaining the findings. Third, although we have taken methodological precautions to ensure that the derived KM curves and HRs are identical or as close to the reported data as possible, we acknowledge there might be some minor differences. Fourth, given a few articles in some CPS subgroups (i.e., CPS ≥ 5 vs. < 5 and CPS ≥ 10 vs. < 10), the findings might be sensitive due to the large errors of ratios although we used the Hartung–Knapp method to correct, and the sensitivity analysis using Bayesian random-effect method showed similar results. The relatively small sample size can lead to increased variability and low statistical power, which makes it challenging to draw confirmative conclusions. Lastly, it is important to recognize that gastric cancer is a heterogeneous disease, and this study primarily focused on the predictive value of PD-L1 without exploring the correlation with other potential efficacy predictors such as MSI-H and EBER. Thus, future studies are highly warranted to validate and expand upon our findings.

In conclusion, our findings suggest a comparable magnitude of benefits between Asian and global patients by adding ICIs to chemotherapy. However, it seems that not all patients could benefit from ICIs, and the optimal CPS threshold at which patients with metastatic GEACs may benefit from ICIs appeared at ≥ 5 for OS and ≥ 10 for PFS. Oncologists should be cautious about using ICIs with chemotherapy in patients with low PD-L1 expression, as more evidence is needed to validate our findings.

### Supplementary Information

Below is the link to the electronic supplementary material.

Supplementary file1 (DOCX 10333 KB)

## Data Availability

The datasets used and/or analyzed during the current study are available from the corresponding author on reasonable request.

## Abbreviations

AACR: American Association for Cancer Research
ASCO: American Society of Clinical Oncology
CI: Confidence interval
CPS: Combined positive score
ESMO: European Society of Medical Oncology
FDA: Food and Drug Administration
GEAC: Gastric and gastroesophageal adenocarcinoma
GEJC: Gastroesophageal junction cancer
HER2: Human epithelial growth factor receptor 2
HR: Hazard ratio
ICI: Immune checkpoint inhibitor
IPD: Individual patient survival data
KM: Kaplan–Meier
NMPA: National Medical Products Administration
ORR: Objective response rate
OS: Overall survival
PD-1: Programmed death-1
PD-L1: Programmed cell death-ligand 1
PFS: Progression-free survival
PRISMA: Preferred Reporting Items for Systematic Reviews and Meta-analyses
RCT: Randomized phase III clinical trial
TPS: Tumor cell proportion score
