# Supplementary material for: The optimal threshold of PD-L1 combined positive score to predict the benefit of PD-1 antibody plus chemotherapy for patients with HER2-negative gastric adenocarcinoma: a meta-analysis
Source: Cancer Immunol Immunother. 2024 May 16;73(7):132. doi: 10.1007/s00262-024-03726-1 (PMC11098986; doi:10.1007/s00262-024-03726-1)
Supplement: Supplementary file 1 — Supplementary file1 (DOCX 10333 KB) [file 262_2024_3726_MOESM1_ESM.docx]

**Supplemental materials**

**Contents**

[Methods 2](#_Toc136012449)

[Table S1. Search information 4](#_Toc136012450)

[Table S2. PRISMA-IPD Checklist of items to include when reporting a systematic review and meta-analysis of individual participant data (IPD) 7](#_Toc136012451)

[Table S3. Risk of bias assessment 12](#_Toc136012452)

[Table S4. Summary of where and how KMSubtraction was implemented 13](#_Toc136012453)

[Table S5. Comparisons of reconstructed KM curves to original curves 15](#_Toc136012454)

[Table S6. Comparisons of KMSubtraction outcomes with reported values for PD-L1 low subgroups 46](#_Toc136012455)

[Table S7. Evaluation of KMSubtraction Bipartite Matching 65](#_Toc136012456)

[Table S8. Convergence plots and histograms of simulations 101](#_Toc136012457)

[Table S9. Sensitivity analysis for the efficacy of ICI plus chemotherapy compared to chemotherapy using Bayesian random-effect method 115](#_Toc136012458)

[Table S10. Sensitivity analysis for the efficacy of ICI plus chemotherapy compared to chemotherapy after excluding ORIENT-16 and ATT-04 from the global analysis 117](#_Toc136012459)

[Figure S1. PRISMA flowchart of study inclusions and exclusions. 118](#_Toc136012460)

[Figure S2. Overall survival curves of ICI plus chemotherapy and chemotherapy groups in based on the reconstructed IPD data 119](#_Toc136012461)

[Figure S3. Progress-free survival curves of ICI plus chemotherapy and chemotherapy groups based on the reconstructed IPD data. 120](#_Toc136012462)

[Figure S4. The funnel plots for OS and PFS. 121](#_Toc136012463)

# Methods

**Reconstruction of IPD time-to-event outcomes**

All relevant publications and the Data Supplements were thoroughly screened to capture any reported KM plots. A graphical reconstructive algorithm was applied to estimate time-to-event outcomes from reported KM curves by methods described by Guyot *et al. {Guyot, 2012 #16} and* Liu *et al. {Liu, 2021 #18}.* The KM curves of the overall cohort and the subgroups with high PD-L1 expression were extracted from the trial articles. Nonetheless, the KM curves from the subgroups with low PD-L1 expression were extracted from the trials when possible and available. For publications where KM curves of the low PD-L1 expression subgroup were unavailable, KMSubtraction was used to retrieve the corresponding IPD survival data from the data for overall patients and the subgroups with high PD-L1 expression {Zhao, 2022 #17}. Minimal-cost bipartite matching was used as the primary algorithm for matching. Monte Carlo simulations with 1000 iterations were conducted to determine the error limits for KMSubtraction.

The reconstruction quality was evaluated by visually comparing reconstructed KM curves and the original published KM curves, marginal HRs with 95% confidence intervals, and at-risk tables. To evaluate the effectiveness of matching, empirical cumulative distribution plots and Bland-Altman plots were used to demonstrate discrepancies in follow-up time between matched pairs. The KM curves of the matched cohorts were also plotted.

**Evaluation of reconstruction and matching**

Time-to-event outcomes were reconstructed from the KM curves of all six trials for the overall and different PD-L1 expression groups. A detailed summary of where KMSubtraction was used to derive subgroup data is shown in the supplement Table S4. The graphical reconstructive algorithm yielded IPD that derived similar HRs and log-rank values to the original plots. A side-by-side comparison of the original and reconstructed curves demonstrated a close match to the original KM curves on visual inspection, marginal HRs, and comparisons of number-at-risk tables (supplement Table S5).

Regarding the subgroups with low PD-L1 expression where the KM curves were unavailable, the KMSubtraction approach derived HRs similar to those previously reported. A side-by-side comparison of the original HRs and the KMSubtraction-derived curves is provided in supplement Table S6. The minimal cost bipartite matching algorithm used to match subgroup patients from the overall cohort achieved optimal matching. There were negligible mean absolute differences in follow-up time between matched pairs on the Bland-Altman plots (as seen by clustering data points near 0) (supplement Table S7). Converted limits of error were reasonable for each implementation of KMSubtraction (supplement Table S8). These results provided confidence to conduct survival analyses of the reconstructed KM plots.

# Table S1. Search information

| Date of search | 2015-01-01 ~ 2023-02-25 |
| --- | --- |
| Databases | PubMed, Cochrane, Embase |
| Pubmed | ("gastrics"[All Fields] OR "stomach"[MeSH Terms] OR "stomach"[All Fields] OR "gastric"[All Fields] OR ("stomach"[MeSH Terms] OR "stomach"[All Fields] OR "stomachs"[All Fields] OR "stomach s"[All Fields] OR "stomachal"[All Fields] OR "stomaches"[All Fields]) OR ("gastro esophageal"[All Fields] OR "gastro oesophageal"[All Fields] OR "gastro oesophagitis"[All Fields] OR "gastroesophagal"[All Fields] OR "gastroesophageal"[All Fields] OR "gastroesophagic"[All Fields] OR "gastroesophagitis"[All Fields] OR "gastrooesophageal"[All Fields]) OR ("gastro esophageal"[All Fields] OR "gastro oesophageal"[All Fields] OR "gastro oesophagitis"[All Fields] OR "gastroesophagal"[All Fields] OR "gastroesophageal"[All Fields] OR "gastroesophagic"[All Fields] OR "gastroesophagitis"[All Fields] OR "gastrooesophageal"[All Fields]) OR ("gastro esophageal"[All Fields] OR "gastro oesophageal"[All Fields] OR "gastro oesophagitis"[All Fields] OR "gastroesophagal"[All Fields] OR "gastroesophageal"[All Fields] OR "gastroesophagic"[All Fields] OR "gastroesophagitis"[All Fields] OR "gastrooesophageal"[All Fields]))  AND ((("cell cycle checkpoints"[MeSH Terms] OR ("cell"[All Fields] AND "cycle"[All Fields] AND "checkpoints"[All Fields]) OR "cell cycle checkpoints"[All Fields] OR "checkpoint"[All Fields] OR "checkpoints"[All Fields]) AND ("antagonists and inhibitors"[MeSH Subheading] OR ("antagonists"[All Fields] AND "inhibitors"[All Fields]) OR "antagonists and inhibitors"[All Fields] OR "inhibitors"[All Fields] OR "inhibitor"[All Fields] OR "inhibitor s"[All Fields])) OR "ICI"[All Fields] OR ("pembrolizumab"[Supplementary Concept] OR "pembrolizumab"[All Fields]) OR ("ipilimumab"[MeSH Terms] OR "ipilimumab"[All Fields]) OR ("nivolumab"[MeSH Terms] OR "nivolumab"[All Fields] OR "nivolumab s"[All Fields]) OR ("avelumab"[Supplementary Concept] OR "avelumab"[All Fields]) OR ("camrelizumab"[Supplementary Concept] OR "camrelizumab"[All Fields]) OR ("durvalumab"[Supplementary Concept] OR "durvalumab"[All Fields]) OR ("sintilimab"[Supplementary Concept] OR "sintilimab"[All Fields]) OR (("program"[All Fields] OR "program s"[All Fields] OR "programe"[All Fields] OR "programed"[All Fields] OR "programes"[All Fields] OR "programing"[All Fields] OR "programmability"[All Fields] OR "programmable"[All Fields] OR "programmably"[All Fields] OR "programme"[All Fields] OR "programme s"[All Fields] OR "programmed"[All Fields] OR "programmer"[All Fields] OR "programmer s"[All Fields] OR "programmers"[All Fields] OR "programmes"[All Fields] OR "programming"[All Fields] OR "programmings"[All Fields] OR "programs"[All Fields]) AND ("death"[MeSH Terms] OR "death"[All Fields] OR "deaths"[All Fields]) AND ("ligand s"[All Fields] OR "liganded"[All Fields] OR "liganding"[All Fields] OR "ligands"[MeSH Terms] OR "ligands"[All Fields] OR "ligand"[All Fields])) OR "PD-L1"[All Fields] OR "PD-1"[All Fields] OR ("immunotherapy"[MeSH Terms] OR "immunotherapy"[All Fields] OR "immunotherapies"[All Fields] OR "immunotherapy s"[All Fields]))  AND ("random*"[All Fields] AND ("clinical trials as topic"[MeSH Terms] OR ("clinical"[All Fields] AND "trials"[All Fields] AND "topic"[All Fields]) OR "clinical trials as topic"[All Fields] OR "trial"[All Fields] OR "trial s"[All Fields] OR "trialed"[All Fields] OR "trialing"[All Fields] OR "trials"[All Fields])) |
| Cochrane | (gastrics) OR "stomach"[MeSH Terms] OR (stomach) OR (gastric) OR (stomach) OR (stomachs) OR (stomach s) OR (stomachal) OR (stomaches) OR (gastro oesophagitis) OR (gastroesophagal) OR (gastroesophageal) OR (gastroesophagic) OR (gastroesophagitis) OR (gastrooesophageal) OR (gastro esophageal) OR (gastro oesophageal) OR (gastro oesophagitis) OR (gastroesophagal) OR (gastroesophageal) OR (gastroesophagic) OR (gastroesophagitis) OR (gastrooesophageal) OR (gastro esophageal) OR (gastro oesophageal) OR (gastro oesophagitis) OR (gastroesophagal) OR (gastroesophageal) OR (gastroesophagic) OR (gastroesophagitis) OR (gastrooesophageal)  AND((((cell) AND (cycle) AND (checkpoints)) OR (cell cycle checkpoints) OR (checkpoint) OR (checkpoints)) AND ((antagonists) AND (inhibitors)) OR (antagonists and inhibitors) OR (inhibitors) OR (inhibitor) OR (inhibitor s))) OR (ICI) OR ((pembrolizumab([Supplementary Concept] OR (pembrolizumab)) OR ((OR (ipilimumab)) OR ((OR (nivolumab) OR (nivolumab s)) OR ((avelumab([Supplementary Concept] OR (avelumab)) OR ((camrelizumab([Supplementary Concept] OR (camrelizumab)) OR ((durvalumab([Supplementary Concept] OR (durvalumab)) OR ((sintilimab([Supplementary Concept] OR (sintilimab)) OR (((program) OR (program s) OR (programe) OR (programed) OR (programes) OR (programing) OR (programmability) OR (programmable) OR (programmably) OR (programme) OR (programme s) OR (programmed) OR (programmer) OR (programmer s) OR (programmers) OR (programmes) OR (programming) OR (programmings) OR (programs)) AND (((death) OR (deaths)) AND ((ligand s) OR (liganded) OR (liganding) OR ( (ligands) OR (ligand))) OR (PD-L1) OR (PD-1) OR ((OR (immunotherapy) OR (immunotherapies) OR (immunotherapy s)))  AND ((random*([All Fields] AND ((clinical trials as topic([MeSH Terms] OR (clinical trials as topic)OR (trial) OR (trial s) OR (trialed) OR (trialing) OR (trials) |
| Embase | 'stomach'/exp OR 'gastrics' OR 'stomach' OR 'gastric' OR 'stomachal' OR ' stomaches’ OR ‘gastro esophageal’ OR 'gastro oesophageal’ OR 'gastro oesophagitis’ OR 'gastroesophagal’ OR 'gastroesophageal’ OR "gastroesophagic’ OR 'gastroesophagitis’ OR 'gastrooesophageal’ OR ‘gastro esophageal’ OR 'gastro oesophageal’ OR 'gastro oesophagitis’ OR 'gastroesophagal’ OR 'gastroesophageal’ OR 'gastroesophagic’ OR 'gastroesophagitis’ OR 'gastrooesophageal’ OR ‘gastro esophageal’ OR 'gastro oesophageal’ OR 'gastro oesophagitis’ OR 'gastroesophagal’ OR 'gastroesophageal’ OR 'gastroesophagic’ OR 'gastroesophagitis’ OR 'gastrooesophageal’  AND('cell cycle checkpoints'/exp OR 'cell cycle checkpoints' OR 'checkpoint' OR 'checkpoints' OR ('antagonists' AND 'inhibitors') OR 'antagonists and inhibitors' OR 'inhibitors' OR 'inhibitor' OR 'inhibitor s' OR 'ici' OR 'pembrolizumab' OR 'ipilimumab'/exp OR 'ipilimumab' OR 'nivolumab'/exp OR 'nivolumab' OR 'nivolumab s' OR 'avelumab' OR 'camrelizumab' OR 'durvalumab' OR 'sintilimab' OR 'program' OR 'program s' OR 'programe' OR 'programed' OR 'programes' OR 'programing' OR 'programmability' OR 'programmable' OR 'programmably' OR 'programme' OR 'programme s' OR 'programmed' OR 'programmer' OR 'programmer s' OR 'programmers' OR 'programmes' OR 'programming' OR 'programmings' OR 'programs') AND 'death'/exp OR 'death' OR 'deaths' OR 'ligand s' OR 'liganded' OR 'liganding' OR 'ligands'/exp OR 'ligands' OR 'ligand' OR 'pd-l1' OR 'pd-1' OR 'immunotherapy'/exp OR 'immunotherapy' OR 'immunotherapies' OR 'immunotherapy s'  AND‘random ‘AND ‘clinical trials as topic’/exp OR ‘clinical trials as topic’OR ‘trial’ OR ‘trial s’ OR ‘trialed’ OR ‘trialing’ OR ‘trials’ |

# Table S2. PRISMA-IPD Checklist of items to include when reporting a systematic review and meta-analysis of individual participant data (IPD)

| **PRISMA-IPD**  **Section/topic** | **Item No** | **Checklist item** | **Reported on page** |
| --- | --- | --- | --- |
| **Title** | | | |
| Title | 1 | Identify the report as a systematic review and meta-analysis of individual participant data. | 1 |
| **Abstract** | | | |
| Structured summary | 2 | Provide a structured summary including as applicable: | 3 |
|  |  | **Purpose**: state the research question and main objectives, with information on participants, interventions, comparators and outcomes. |  |
|  |  | **Patients and methods**: report eligibility criteria; data sources including dates of last bibliographic search or elicitation, noting that IPD were sought; methods of assessing the risk of bias. |  |
|  |  | **Results**: provide the number and type of studies and participants identified and the number (%) obtained; summary effect estimates for main outcomes (benefits and harms) with confidence intervals and measures of statistical heterogeneity. Describe the direction and size of summary effects in terms meaningful to those who would put findings into practice. |  |
|  |  | **Conclusion:** state the main strengths and limitations of the evidence, the general interpretation of the results and any important implications. |  |
|  |  | **Other:** report primary funding source, registration number and registry name for the systematic review and IPD meta-analysis. |  |
| **Introduction** | | | |
| Rationale | 3 | Describe the rationale for the review in the context of what is already known. | 5 |
| Objectives | 4 | Provide an explicit statement of the questions being addressed with reference, as applicable, to participants, interventions, comparisons, outcomes and study design (PICOS). Include any hypotheses that relate to particular types of participant-level subgroups. | 5-6 |
| **Methods** | | | |
| Protocol and registration | 5 | Indicate if a protocol exists and where it can be accessed. If available, provide registration information, including the registration number and registry name. Provide publication details, if applicable. | 6 |
| Eligibility criteria | 6 | Specify inclusion and exclusion criteria, including those relating to participants, interventions, comparisons, outcomes, study design and characteristics (e.g., years when conducted, required minimum follow-up). Note whether these were applied at the study or individual level, i.e., whether eligible participants were included (and ineligible participants excluded) from a study that included a wider population than specified by the review inclusion criteria. The rationale for the criteria should be stated. | 6 |
| Identifying studies - information sources | 7 | Describe all methods of identifying published and unpublished studies, including, as applicable: which bibliographic databases were searched with dates of coverage; details of any hand searching including of conference proceedings; use of study registers and agency or company databases; contact with the original research team and experts in the field; open adverts and surveys. Give the date of the last search or elicitation. | 6 |
| Identifying studies - search | 8 | Present the full electronic search strategy for at least one database, including any limits used, such that it could be repeated. | 6-7 |
| Study selection processes | 9 | State the process for determining which studies were eligible for inclusion. | 6-7 |
| Data collection processes | 10 | Describe how IPDs were requested, collected and managed, including any processes for querying and confirming data with investigators. If IPD were not sought from any eligible study, the reason for this should be stated (for each such study). | 7 |
|  |  | If applicable, describe how any studies for which IPD were not available were dealt with. This should include whether, how and what aggregate data were sought or extracted from study reports and publications (such as extracting data independently in duplicate) and any processes for obtaining and confirming these data with investigators. |  |
| Data items | 11 | Describe how the information and variables to be collected were chosen. List and define all study and participant-level data sought, including baseline and follow-up information. If applicable, describe methods of standardizing or translating variables within the IPD datasets to ensure common scales or measurements across studies. | 7 |
| IPD integrity | A1 | Describe what aspects of IPD were subject to data checking (such as sequence generation, data consistency and completeness, and baseline imbalance) and how this was done. | 7, Supplemental methods |
| Risk of bias assessment in individual studies. | 12 | Describe methods used to assess the risk of bias in the individual studies and whether this was applied separately for each outcome. If applicable, describe how findings of IPD checking were used to inform the assessment. Report if and how the risk of bias assessment was used in any data synthesis. | 7 |
| Specification of outcomes and effect measures | 13 | State all treatment comparisons of interests. State all outcomes addressed and define them in detail. State whether they were pre-specified for the review and, if applicable, whether they were primary/main or secondary/additional outcomes. Give the principal measures of effect (such as risk ratio, hazard ratio, and difference in means) used for each outcome. | 7 |
| Synthesis methods | 14 | Describe the meta-analysis methods used to synthesize IPD. Specify any statistical methods and models used. Issues should include (but are not restricted to):   - Use of a one-stage or two-stage approach. - How effect estimates were generated separately within each study and combined across studies (where applicable). - Specification of one-stage models (where applicable), including how clustering of patients within studies was accounted for. - Use of fixed or random effects models and any other model assumptions, such as proportional hazards. - How (summary) survival curves were generated (where applicable). - Methods for quantifying statistical heterogeneity (such as I^2^ and τ^2^). - How studies providing IPD and not providing IPD were analyzed together (where applicable). - How missing data within the IPD were dealt with (where applicable). | 8 |
| Exploration of variation in effects | A2 | If applicable, describe any methods used to explore effect variation by study or participant level characteristics (such as estimating interactions between effect and covariates). State all participant-level characteristics analyzed as potential effect modifiers and whether these were pre-specified. | 7 |
| Risk of bias across studies | 15 | Specify any assessment of the risk of bias relating to the accumulated body of evidence, including any pertaining to not obtaining IPD for particular studies, outcomes or other variables. | 7 |
| Additional analyses | 16 | Describe methods of any additional analyses, including sensitivity analyses. State which of these were pre-specified. | 8 |
| **Results** | | | |
| Study selection and IPD obtained | 17 | Give the number of studies screened, assessed for eligibility, and included in the systematic review with reasons for exclusions at each stage. Indicate the number of studies and participants for which IPDs were sought and for which IPDs were obtained. For those studies where IPD was not available, give the numbers of studies and participants for which aggregate data were available. Report reasons for non-availability of IPD. Include a flow diagram. | 9 |
| Study characteristics | 18 | For each study, present information on key study and participant characteristics (such as the description of interventions, numbers of participants, demographic data, unavailability of outcomes, funding source, and, if applicable, duration of follow-up). Provide (main) citations for each study. Where applicable, also report similar study characteristics for any studies not providing IPD. | 9 |
| IPD integrity | A3 | Report any important issues identified in checking IPD or state that there were none. | 7 |
| Risk of bias within studies | 19 | Present data on the risk of bias assessments. If applicable, describe whether data checking led to the up-weighting or down-weighting of these assessments. Consider how any potential bias impacts the robustness of meta-analysis conclusions. | 7 |
| Results of individual studies | 20 | For each comparison and each main outcome (benefit or harm), each study reports the number of eligible participants for which data were obtained and shows simple summary data for each intervention group (including, where applicable, the number of events), effect estimates and confidence intervals. These may be tabulated or included on a forest plot. | 10-12 |
| Results of syntheses | 21 | Present summary effects for each meta-analysis undertaken, including confidence intervals and measures of statistical heterogeneity. State whether the analysis was pre-specified, and report the number of studies and participants and, where applicable, the number of events on which it is based. | 10-12 |
|  |  | When exploring variation in effects due to patient or study characteristics, present summary interaction estimates for each characteristic examined, including confidence intervals and measures of statistical heterogeneity. State whether the analysis was pre-specified. State whether any interaction is consistent across trials. |  |
|  |  | Describe the direction and size of the effect in terms meaningful to those who would put findings into practice. |  |
| Risk of bias across studies | 22 | Present results of any assessment of the risk of bias relating to the accumulated body of evidence, including any availability and representativeness of available studies, outcomes or other variables. | 7 |
| Additional analyses | 23 | Give results of any additional analyses (e.g., sensitivity analyses). If applicable, this should also include any analyses incorporating aggregate data for studies that do not have IPD. If applicable, summarise the main meta-analysis results following the inclusion or exclusion of studies for which IPDs were not available. | 8 |
| **Discussion** | | | |
| Summary of evidence | 24 | Summarise the main findings, including the strength of evidence for each main outcome. | 12 |
| Strengths and limitations | 25 | Discuss any important strengths and limitations of the evidence, including the benefits of access to IPD and any limitations arising from IPD that were not available. | 14 |
| Conclusions | 26 | Provide a general interpretation of the findings in the context of other evidence. | 14-15 |
| Implications | A4 | Consider relevance to key groups (such as policymakers, service providers and service users). Consider implications for future research. | 15 |
| **Funding** | | | |
| Funding | 27 | Describe sources of funding and other support (such as the supply of IPD) and the role in the systematic review of those providing such support. | 15 |

# Table S3. Risk of bias assessment


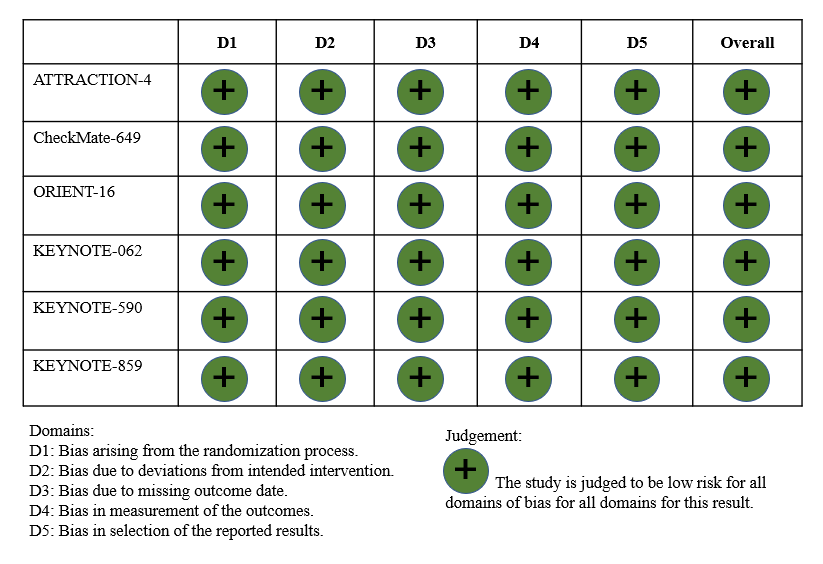


# Table S4. Summary of where and how KMSubtraction was implemented

| Trail | Comparison | Outcome | Histology | “Overall” curve | Subgroup High PD-L1 expressing curve | Did the original publication report the low PD-L1 KM curve? | Did the original publication report the low PD-L1 Hazard ratio? | Derived curves by KM Subtraction: i.e., “Subtracting” “Subgroup” curve from the “overall” curve |
| --- | --- | --- | --- | --- | --- | --- | --- | --- |
| Attraction-4 | Nivo+Chemo/Chemo | OS | G/GEJ cancer | regardless of PD-L1 expression | NA | No | Yes | NA |
|  |  |  |  |  |  |  |  |  |
|  | Nivo+Chemo/Chemo | PFS | G/GEJ cancer | regardless of PD-L1 expression | NA | No | Yes | NA |
| CheckMate-649  (both global and Chinese populations) | Nivo+Chemo/Chemo | OS | Advanced or metastatic gastric, GEJ, or oesophageal adenocarcinoma | regardless of PD-L1 expression | CPS ≥1; | No | Yes (CPS<1, CPS<5) | CPS <1;  CPS <5;  1≤CPS <5 |
|  |  |  |  |  | CPS ≥5 |  |  |  |
|  | Nivo+Chemo/Chemo | PFS | Advanced or metastatic gastric, GEJ, or oesophageal adenocarcinoma | regardless of PD-L1 expression | CPS ≥1; | No | Yes (CPS<1, CPS<5) | CPS <1;  CPS <5;  1≤CPS <5 |
|  |  |  |  |  | CPS ≥5 |  |  |  |
| ORIENT-16 | Sintili+Chemo/Chemo | OS | G/GEJ adenocarcinoma | regardless of PD-L1 expression | CPS ≥5 | No | No | CPS <5 |
|  | Sintili+Chemo/Chemo | PFS | G/GEJ adenocarcinoma | regardless of PD-L1 expression | CPS ≥5 | No | No | CPS <5 |
| Keynote-062  (both global and Asian populations) | Pembro+Chemo/Chemo | OS | G/GEJ adenocarcinoma | PD-L1 CPS ≥1 | CPS ≥10 | No | No | 1≤ CPS <10 |
|  | Pembro+Chemo/Chemo | PFS | G/GEJ adenocarcinoma | PD-L1 CPS ≥1 | CPS ≥10 | No | No | 1≤ CPS <10 |
| Keynote-590 | Pembro+Chemo/Chemo | OS | gastro-oesophageal junction cancer | regardless of PD-L1 expression | CPS ≥10 | Yes | Yes (CPS<10) | NA |
|  | Pembro+Chemo/Chemo | PFS | gastro-oesophageal junction cancer | regardless of PD-L1 expression | CPS ≥10 | Yes | Yes (CPS<10) | NA |
| Keynote-859 | Pembro+Chemo/Chemo | OS | advanced gastric/gastroesophageal  junction (G/GEJ) cancer | regardless of PD-L1 expression | NA | No | Yes (CPS<1, CPS<10) | NA |
|  | Pembro+Chemo/Chemo | PFS | advanced gastric/gastroesophageal  junction (G/GEJ) cancer | regardless of PD-L1 expression | NA | No | Yes (CPS<1, CPS<10) | NA |

#

# Table S5. Comparisons of reconstructed KM curves to original curves

| **Study** | **Cohort** | **Outcome** | **Original** | **Reconstructed** |
| --- | --- | --- | --- | --- |
| Attraction-04 | Overall cohort | OS | The original figure may be found in the primary trial manuscript.  Kang YK, Chen LT, Ryu MH, et al.: Nivolumab plus chemotherapy versus placebo plus chemotherapy in patients with HER2-negative, untreated, unresectable advanced or recurrent gastric or gastro-oesophageal junction cancer (ATTRACTION-4): a randomized, multicentre, double-blind, placebo-controlled, phase 3 trial. Lancet Oncol. 2022 Feb;23(2):234-247.  Figure 2B |  |
|  | Overall cohort | PFS | The original figure may be found in the primary trial manuscript.  Kang YK, Chen LT, Ryu MH, et al.: Nivolumab plus chemotherapy versus placebo plus chemotherapy in patients with HER2-negative, untreated, unresectable advanced or recurrent gastric or gastro-oesophageal junction cancer (ATTRACTION-4): a randomized, multicentre, double-blind, placebo-controlled, phase 3 trial. Lancet Oncol. 2022 Feb;23(2):234-247.  Figure 2A |  |
| CheckMate-649 | Overall cohort | OS | The original figure may be found in the primary trial manuscript.  Janjigian YY, Shitara K, Moehler M, et al.: First-line nivolumab plus chemotherapy versus chemotherapy alone for advanced gastric, gastro-oesophageal junction, and oesophageal adenocarcinoma (CheckMate 649): A randomized, open-label, phase 3 trial. Lancet 398:27-40, 2021  Figure 2C |  |
|  | Overall cohort | PFS | The original figure may be found in the primary trial manuscript.  Janjigian YY, Shitara K, Moehler M, et al.: First-line nivolumab plus chemotherapy versus chemotherapy alone for advanced gastric, gastro-oesophageal junction, and oesophageal adenocarcinoma (CheckMate 649): A randomized, open-label, phase 3 trial. Lancet 398:27-40, 2021  Figure 3C |  |
|  | CPS≥5 | OS | The original figure may be found in the primary trial manuscript.  Janjigian YY, Shitara K, Moehler M, et al.: First-line nivolumab plus chemotherapy versus chemotherapy alone for advanced gastric, gastro-oesophageal junction, and oesophageal adenocarcinoma (CheckMate 649): A randomized, open-label, phase 3 trial. Lancet 398:27-40, 2021  Figure 2A |  |
|  | CPS≥5 | PFS | The original figure may be found in the primary trial manuscript.  Janjigian YY, Shitara K, Moehler M, et al.: First-line nivolumab plus chemotherapy versus chemotherapy alone for advanced gastric, gastro-oesophageal junction, and oesophageal adenocarcinoma (CheckMate 649): A randomized, open-label, phase 3 trial. Lancet 398:27-40, 2021  Figure 3A |  |
|  | CPS≥1 | OS | The original figure may be found in the primary trial manuscript.  Janjigian YY, Shitara K, Moehler M, et al.: First-line nivolumab plus chemotherapy versus chemotherapy alone for advanced gastric, gastro-oesophageal junction, and oesophageal adenocarcinoma (CheckMate 649): A randomized, open-label, phase 3 trial. Lancet 398:27-40, 2021  Figure 2B |  |
|  | CPS≥1 | PFS | The original figure may be found in the primary trial manuscript.  Janjigian YY, Shitara K, Moehler M, et al.: First-line nivolumab plus chemotherapy versus chemotherapy alone for advanced gastric, gastro-oesophageal junction, and oesophageal adenocarcinoma (CheckMate 649): A randomized, open-label, phase 3 trial. Lancet 398:27-40, 2021  Figure 3B |  |
| CheckMate-649, Chinese subgroup | Overall cohort | OS | The original figure may be found in the primary trial manuscript.  Liu T, Bai Y, Lin X, Li W, et al.: First-line nivolumab plus chemotherapy vs. chemotherapy in patients with advanced gastric, gastroesophageal junction and esophageal adenocarcinoma: CheckMate 649 Chinese subgroup analysis. Int J Cancer. 2023 Feb 15;152(4):749-760.  Figure 2C |  |
|  | Overall cohort | PFS | The original figure may be found in the primary trial manuscript.  Liu T, Bai Y, Lin X, Li W, et al.: First-line nivolumab plus chemotherapy vs. chemotherapy in patients with advanced gastric, gastroesophageal junction and esophageal adenocarcinoma: CheckMate 649 Chinese subgroup analysis. Int J Cancer. 2023 Feb 15;152(4):749-760.  Figure 3C |  |
|  | CPS≥5 | OS | The original figure may be found in the primary trial manuscript.  Liu T, Bai Y, Lin X, Li W, et al.: First-line nivolumab plus chemotherapy vs. chemotherapy in patients with advanced gastric, gastroesophageal junction and esophageal adenocarcinoma: CheckMate 649 Chinese subgroup analysis. Int J Cancer. 2023 Feb 15;152(4):749-760.  Figure 2A |  |
|  | CPS≥5 | PFS | The original figure may be found in the primary trial manuscript.  Liu T, Bai Y, Lin X, Li W, et al.: First-line nivolumab plus chemotherapy vs. chemotherapy in patients with advanced gastric, gastroesophageal junction and esophageal adenocarcinoma: CheckMate 649 Chinese subgroup analysis. Int J Cancer. 2023 Feb 15;152(4):749-760.  Figure 3A |  |
|  | CPS≥1 | OS | The original figure may be found in the primary trial manuscript.  Liu T, Bai Y, Lin X, Li W, et al.: First-line nivolumab plus chemotherapy vs. chemotherapy in patients with advanced gastric, gastroesophageal junction and esophageal adenocarcinoma: CheckMate 649 Chinese subgroup analysis. Int J Cancer. 2023 Feb 15;152(4):749-760.  Figure 2B |  |
|  | CPS≥1 | PFS | The original figure may be found in the primary trial manuscript.  Liu T, Bai Y, Lin X, Li W, et al.: First-line nivolumab plus chemotherapy vs. chemotherapy in patients with advanced gastric, gastroesophageal junction and esophageal adenocarcinoma: CheckMate 649 Chinese subgroup analysis. Int J Cancer. 2023 Feb 15;152(4):749-760.  Figure 3B |  |
| ORIENT-16 | Overall cohort | OS | The original figure may be found in the primary trial manuscript.  Jianming Xu, H Jiang, Y Pan, K Gu, et al.: LBA53 - Sintilimab plus chemotherapy (chemo) versus chemo as first-line treatment for advanced gastric or gastroesophageal junction (G/GEJ) adenocarcinoma (ORIENT-16): First results of a randomized, double-blind, phase III study. Presented at the ESMO Congress 2021, 17 Sep, 2021 |  |
|  | Overall cohort | PFS | The original figure may be found in the primary trial manuscript.  Jianming Xu, H Jiang, Y Pan, K Gu, et al.: LBA53 - Sintilimab plus chemotherapy (chemo) versus chemo as first-line treatment for advanced gastric or gastroesophageal junction (G/GEJ) adenocarcinoma (ORIENT-16): First results of a randomized, double-blind, phase III study. Presented at the ESMO Congress 2021, 17 Sep, 2021 |  |
|  | CPS≥5 | OS | The original figure may be found in the primary trial manuscript.  Jianming Xu, H Jiang, Y Pan, K Gu, et al.: LBA53 - Sintilimab plus chemotherapy (chemo) versus chemo as first-line treatment for advanced gastric or gastroesophageal junction (G/GEJ) adenocarcinoma (ORIENT-16): First results of a randomized, double-blind, phase III study. Presented at the ESMO Congress 2021, 17 Sep, 2021 |  |
|  | CPS≥5 | PFS | The original figure may be found in the primary trial manuscript.  Jianming Xu, H Jiang, Y Pan, K Gu, et al.: LBA53 - Sintilimab plus chemotherapy (chemo) versus chemo as first-line treatment for advanced gastric or gastroesophageal junction (G/GEJ) adenocarcinoma (ORIENT-16): First results of a randomized, double-blind, phase III study. Presented at the ESMO Congress 2021, 17 Sep, 2021 |  |
| Keynote-062 | Overall cohort | OS | The original figure may be found in the primary trial manuscript and supplemental content.  Shitara K, Van Cutsem E, Bang YJ, et al: Efficacy and Safety of Pembrolizumab or Pembrolizumab Plus Chemotherapy vs Chemotherapy Alone for Patients With First-line, Advanced Gastric Cancer: The KEYNOTE-062 Phase 3 Randomized Clinical Trial. JAMA Oncol. 2020 Oct 1;6(10):1571-1580.  Figure 2C |  |
|  | Overall cohort | PFS | The original figure may be found in the primary trial manuscript and supplemental content.  Shitara K, Van Cutsem E, Bang YJ, et al: Efficacy and Safety of Pembrolizumab or Pembrolizumab Plus Chemotherapy vs Chemotherapy Alone for Patients With First-line, Advanced Gastric Cancer: The KEYNOTE-062 Phase 3 Randomized Clinical Trial. JAMA Oncol. 2020 Oct 1;6(10):1571-1580.  eFigure 4C |  |
|  | CPS≥10 | OS | The original figure may be found in the primary trial manuscript and supplemental content.  Shitara K, Van Cutsem E, Bang YJ, et al: Efficacy and Safety of Pembrolizumab or Pembrolizumab Plus Chemotherapy vs Chemotherapy Alone for Patients With First-line, Advanced Gastric Cancer: The KEYNOTE-062 Phase 3 Randomized Clinical Trial. JAMA Oncol. 2020 Oct 1;6(10):1571-1580.  Figure 2D |  |
|  | CPS≥10 | PFS | The original figure may be found in the primary trial manuscript and supplemental content.  Shitara K, Van Cutsem E, Bang YJ, et al: Efficacy and Safety of Pembrolizumab or Pembrolizumab Plus Chemotherapy vs Chemotherapy Alone for Patients With First-line, Advanced Gastric Cancer: The KEYNOTE-062 Phase 3 Randomized Clinical Trial. JAMA Oncol. 2020 Oct 1;6(10):1571-1580.  eFigure 4D |  |
| Keynote-062, Asian subgroup | Overall cohort | OS | The original figure may be found in the primary trial manuscript.  Satake H, Lee KW, Chung HC, et al.: Pembrolizumab or pembrolizumab plus chemotherapy versus standard of care chemotherapy in patients with advanced gastric or gastroesophageal junction adenocarcinoma: Asian subgroup analysis of KEYNOTE-062. Jpn J Clin Oncol. 2022 Dec 18:hyac188.  Figure 1C |  |
|  | Overall cohort | PFS | The original figure may be found in the primary trial manuscript.  Satake H, Lee KW, Chung HC, et al.: Pembrolizumab or pembrolizumab plus chemotherapy versus standard of care chemotherapy in patients with advanced gastric or gastroesophageal junction adenocarcinoma: Asian subgroup analysis of KEYNOTE-062. Jpn J Clin Oncol. 2022 Dec 18:hyac188.  Figure 2C |  |
|  | CPS≥10 | OS | The original figure may be found in the primary trial manuscript.  Satake H, Lee KW, Chung HC, et al.: Pembrolizumab or pembrolizumab plus chemotherapy versus standard of care chemotherapy in patients with advanced gastric or gastroesophageal junction adenocarcinoma: Asian subgroup analysis of KEYNOTE-062. Jpn J Clin Oncol. 2022 Dec 18:hyac188.  Figure 1D |  |
|  | CPS≥10 | PFS | The original figure may be found in the primary trial manuscript.  Satake H, Lee KW, Chung HC, et al.: Pembrolizumab or pembrolizumab plus chemotherapy versus standard of care chemotherapy in patients with advanced gastric or gastroesophageal junction adenocarcinoma: Asian subgroup analysis of KEYNOTE-062. Jpn J Clin Oncol. 2022 Dec 18:hyac188.  Figure 2D |  |
| Keynote-590 | Overall cohort | OS | The original figure may be found in the primary trial manuscript.  Sun JM, Shen L, Shah MA, et al. Pembrolizumab plus chemotherapy versus chemotherapy alone for first-line treatment of advanced oesophageal cancer (KEYNOTE-590): a randomized, placebo-controlled, phase 3 study. Lancet. 2021;398(10302):759-71.  Figure 2D |  |
|  | CPS≥10 | OS | The original figure may be found in the primary trial manuscript.  Sun JM, Shen L, Shah MA, et al. Pembrolizumab plus chemotherapy versus chemotherapy alone for first-line treatment of advanced oesophageal cancer (KEYNOTE-590): a randomized, placebo-controlled, phase 3 study. Lancet. 2021;398(10302):759-71.  Figure 2C |  |
|  | Overall cohort | PFS | The original figure may be found in the primary trial manuscript.  Sun JM, Shen L, Shah MA, et al. Pembrolizumab plus chemotherapy versus chemotherapy alone for first-line treatment of advanced oesophageal cancer (KEYNOTE-590): a randomized, placebo-controlled, phase 3 study. Lancet. 2021;398(10302):759-71.  Figure 4C |  |
| Keynote-859 | Overall cohort | OS | S. Y. Rha, LSW, P. E. Y. Weber, et al. Pembrolizumab plus chemotherapy as first-line therapy for advanced HER2-negative gastric or gastroesophageal junction cancer: Phase III KEYNOTE-859 study. ESMO VIRTUAL PLENARY ABSTRACT; February 16: Annals of Oncology; 2023; <https://doi>.org/10.1016/j.annonc.2023.01.006 |  |
|  | Overall cohort | PFS | S. Y. Rha, LSW, P. E. Y. Weber, et al. Pembrolizumab plus chemotherapy as first-line therapy for advanced HER2-negative gastric or gastroesophageal junction cancer: Phase III KEYNOTE-859 study. ESMO VIRTUAL PLENARY ABSTRACT; February 16: Annals of Oncology; 2023; <https://doi>.org/10.1016/j.annonc.2023.01.006 |  |

# Table S6. Comparisons of KMSubtraction outcomes with reported values for PD-L1 low subgroups

| Comparison | PD-L1 expression subgroup | Outcome | Reported | | KMSubtraction with bipartite matching |
| --- | --- | --- | --- | --- | --- |
|  |  |  | Hazard ratio (95%CI) | P value | Curve, hazard ratio (95% CI) |
| CheckMate-649, Global | | | | | |
| Nivo+Chemo vs. Chemo | CPS<5 | OS | 0.94 (0.78-1.13) | NR |  |
| Nivo+Chemo vs. Chemo | CPS<1 | OS | 0.92 (0.70-1.23) | NR |  |
| Nivo+Chemo vs. Chemo | 1≤CPS<5 | OS | NR | NR |  |
| Nivo+Chemo vs. Chemo | CPS<5 | PFS | 0.93 (0.76-1.12) | NR |  |
| Nivo+Chemo vs. Chemo | CPS<1 | PFS | 0.93 (0.69-1.26) | NR |  |
| Nivo+Chemo vs. Chemo | 1≤CPS<5 | PFS | NR | NR |  |
| CheckMate-649, Chinese subgroup | | | | | |
| Nivo+Chemo vs. Chemo | CPS<5 | OS | 0.81 (0.44-1.48) | NR |  |
| Nivo+Chemo vs. Chemo | CPS<1 | OS | 0.69 (0.27-1.75) | NR |  |
| Nivo+Chemo vs. Chemo | 1≤CPS<5 | OS | NR | NR |  |
| Nivo+Chemo vs. Chemo | CPS<5 | PFS | 0.70 (0.36-1.34) | NR |  |
| Nivo+Chemo vs. Chemo | CPS<1 | PFS | 0.51 (0.18-1.45) | NR |  |
| Nivo+Chemo vs. Chemo | 1≤CPS<5 | PFS | NA | NR |  |
| ORIENT-16 | | | | | |
| Sintili+Chemo vs. Chemo | CPS<5 | OS | NR | NR |  |
| Sintili+Chemo vs. Chemo | CPS<5 | PFS | NR | NR |  |
| Keynote-062, global | | | | | |
| Pembro+Chemo vs. Chemo | 1≤CPS<10 | OS | NR | NR |  |
| Pembro+Chemo vs. Chemo | 1≤CPS<10 | PFS | NR | NR |  |
| Keynote-062, Asian subgroup | | | | | |
| Pembro+Chemo vs. Chemo | 1≤CPS<10 | OS | NR | NR |  |
| Pembro+Chemo vs. Chemo | 1≤CPS<10 | PFS | NR | NR |  |
| Keynote-590 |  |  |  |  |  |
| Pembro+Chemo vs. Chemo | CPS<10 | OS | 0.66 (0.42-1.04) | NR |  |

# Table S7. Evaluation of KMSubtraction Bipartite Matching

| Empirical cumulative distribution between follow-up time of matched pairs and BlandAltman plots to explore discrepancies between matched pairs | Kaplan Meier Curves and Cox-proportional hazard’s model comparing matched pairs |
| --- | --- |
| CheckMate-649: global patients | |
| Overall survival: CPS<5 for ICI plus chemotherapy  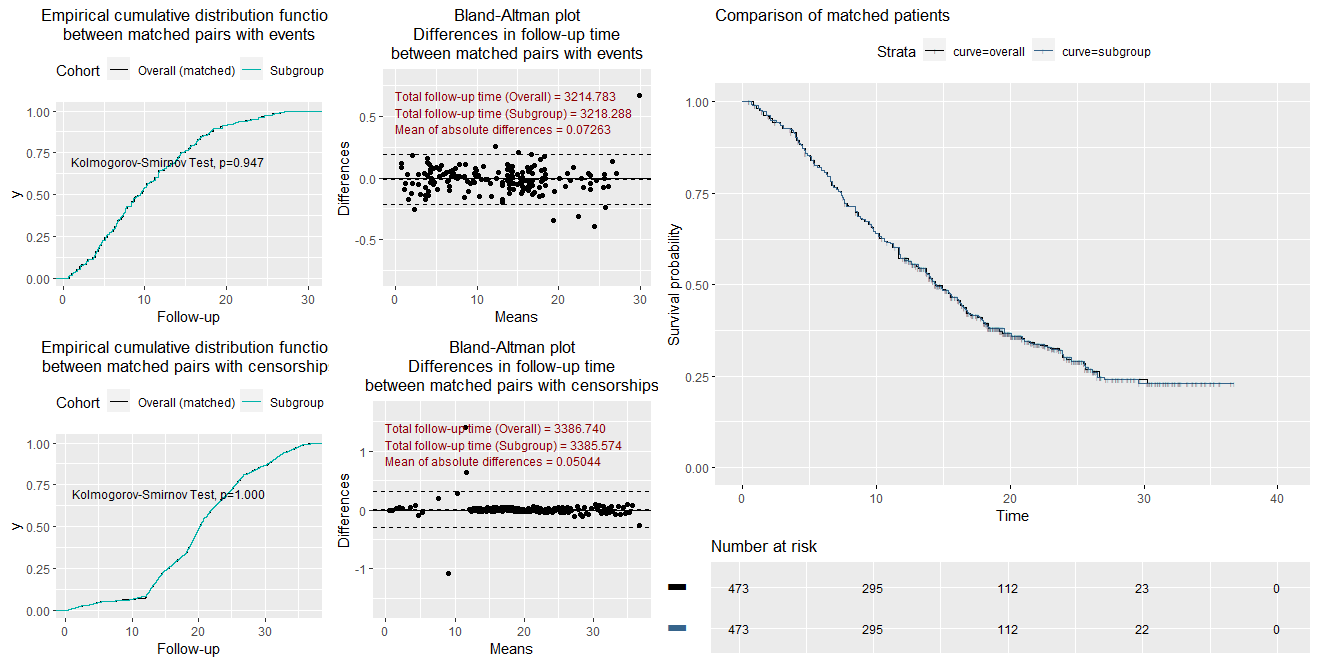 | |
| Overall survival: CPS<1 for ICI plus chemotherapy  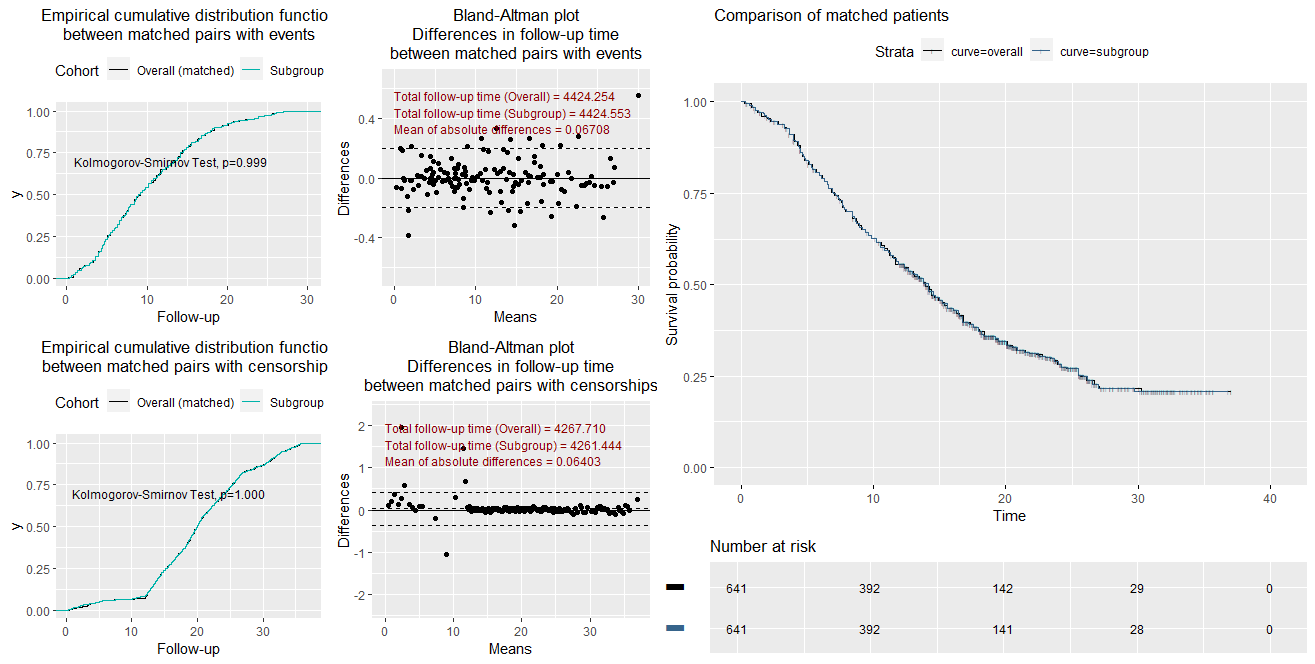 | |
| Overall survival: CPS<5 for chemotherapy  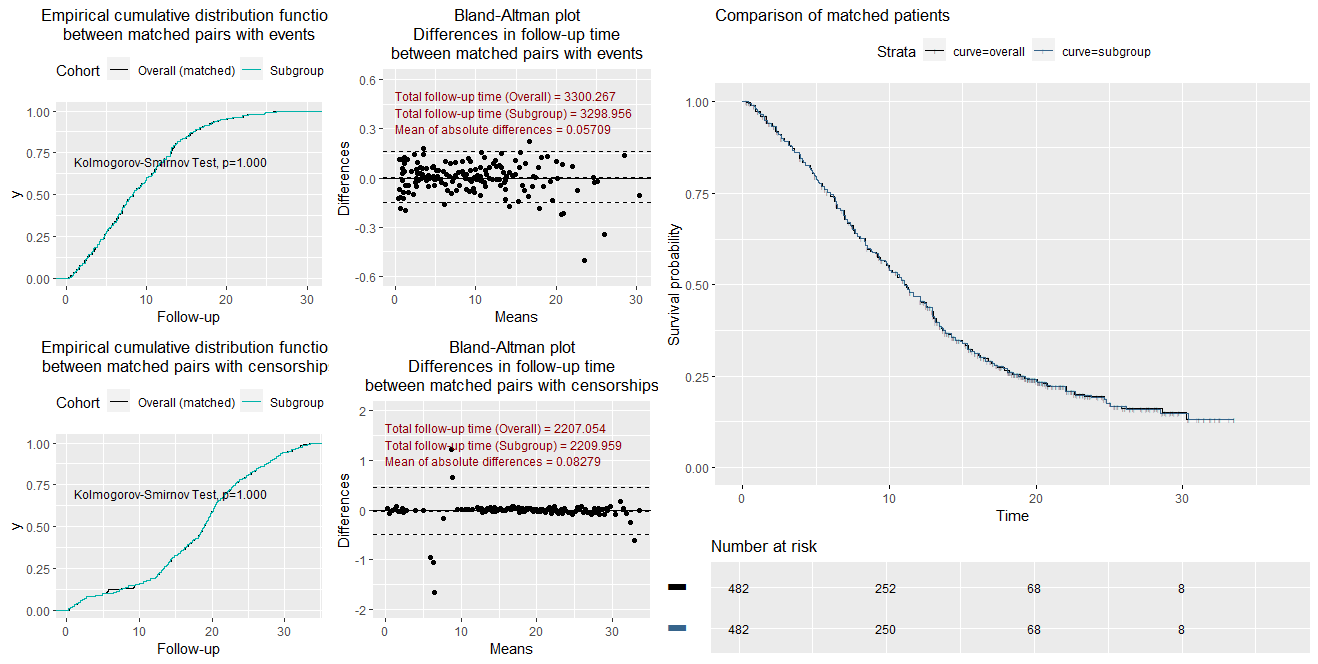 | |
| Overall survival: CPS<1 for chemotherapy  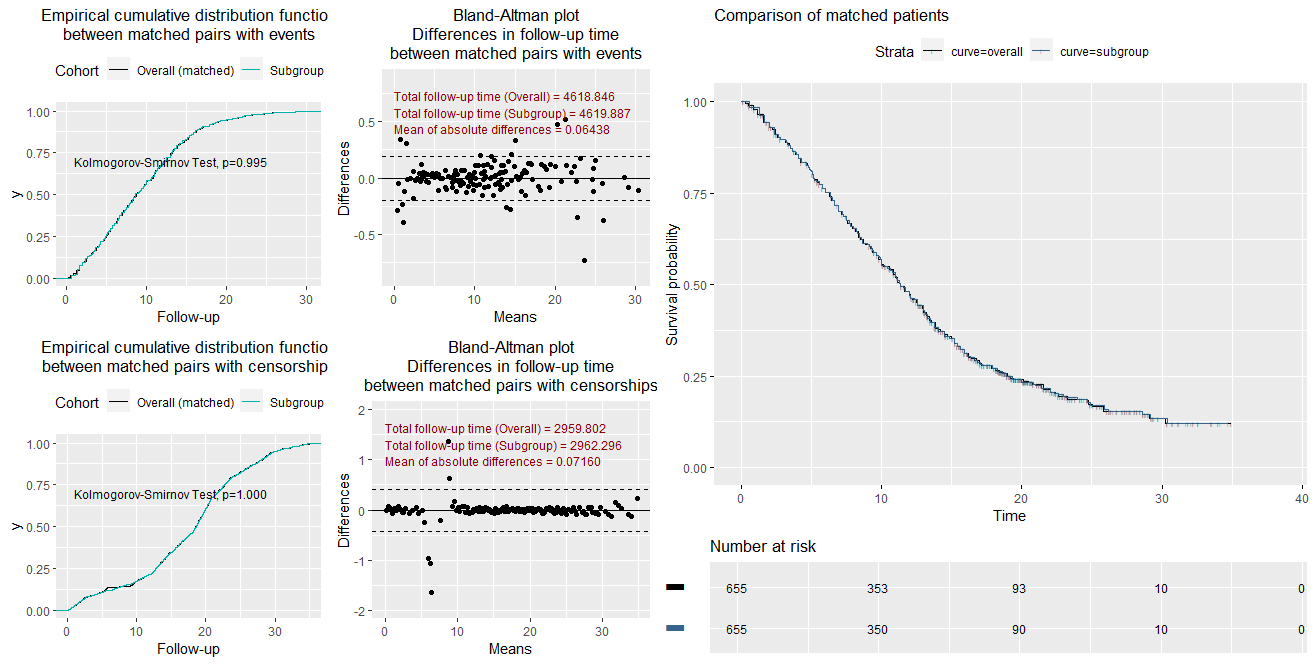 | |
| PFS: CPS<5 for ICI plus chemotherapy  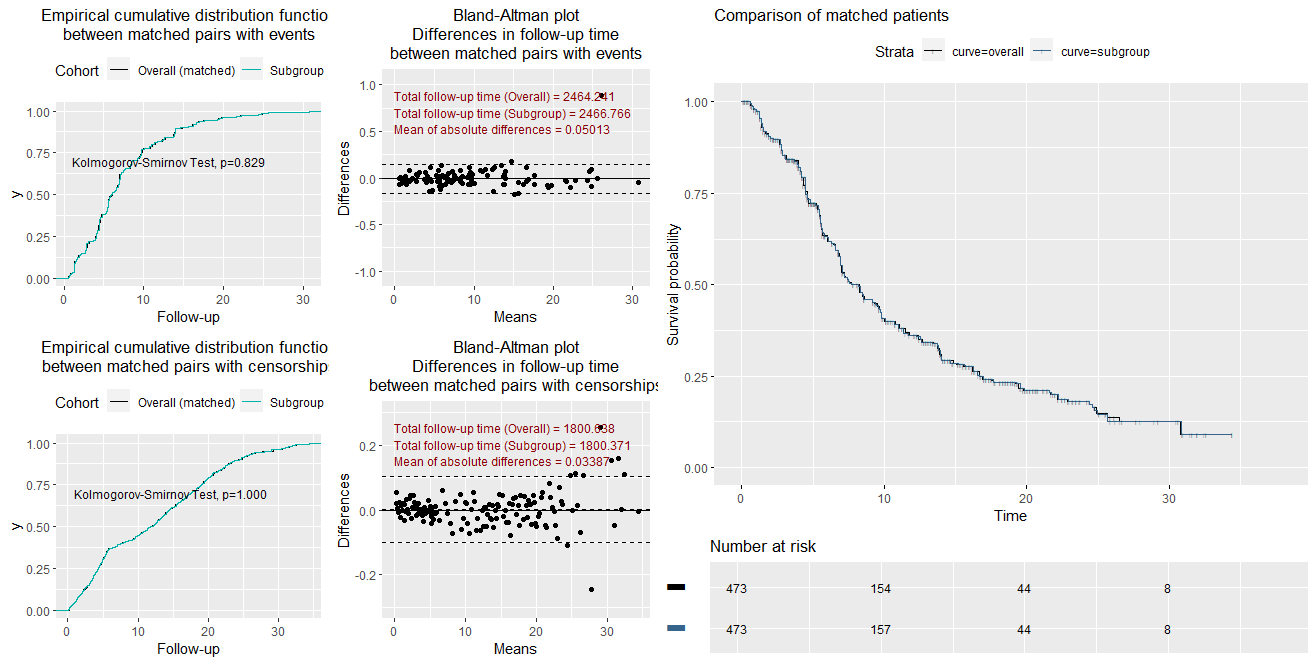 | |
| PFS: CPS<1 for ICI plus chemotherapy  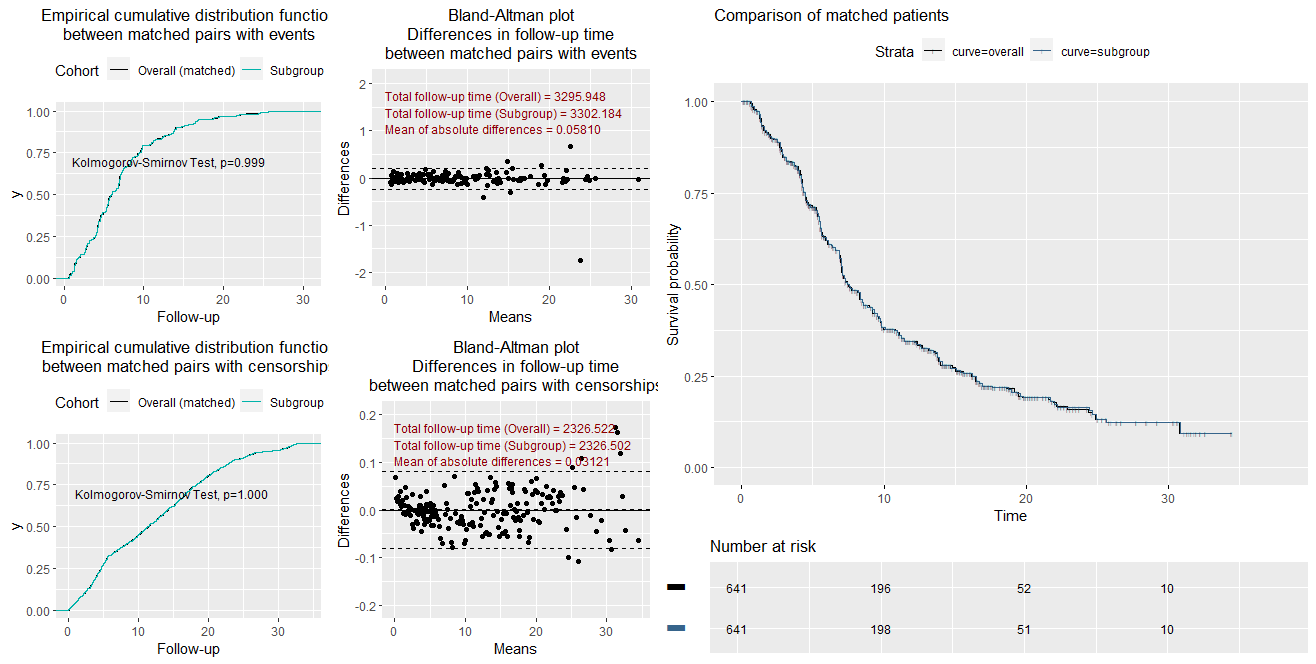 | |
| PFS: CPS<5 for chemotherapy  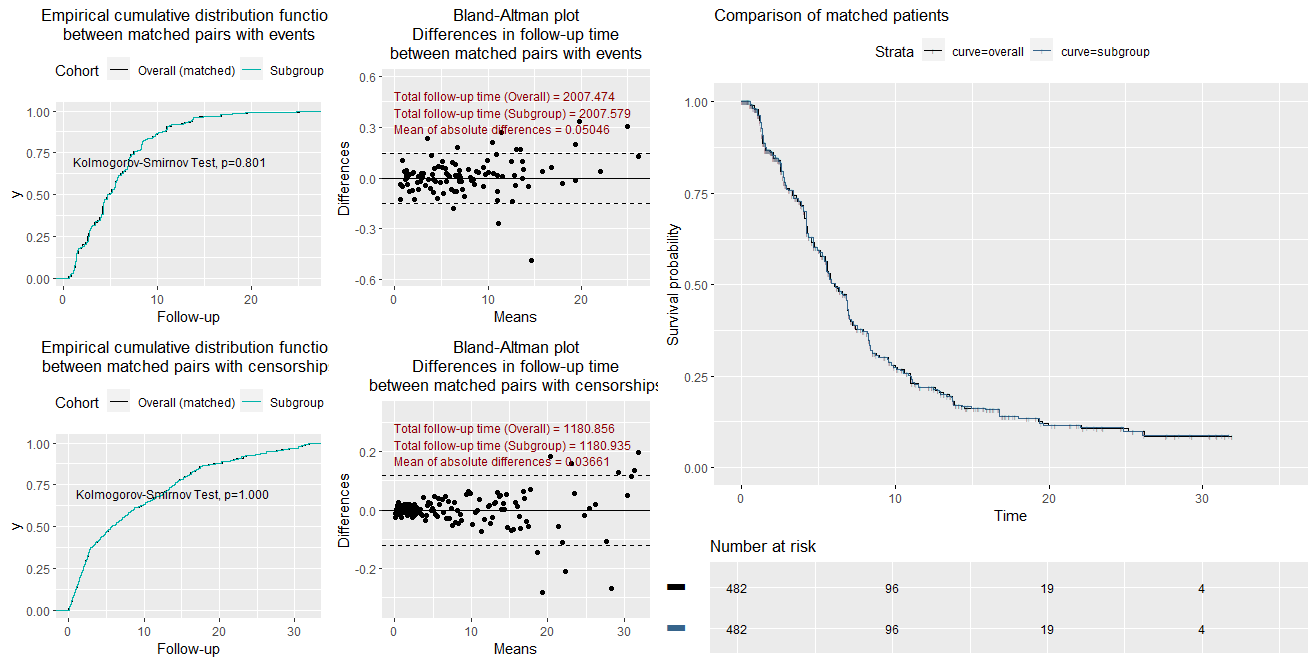 | |
| PFS: CPS<1 for chemotherapy  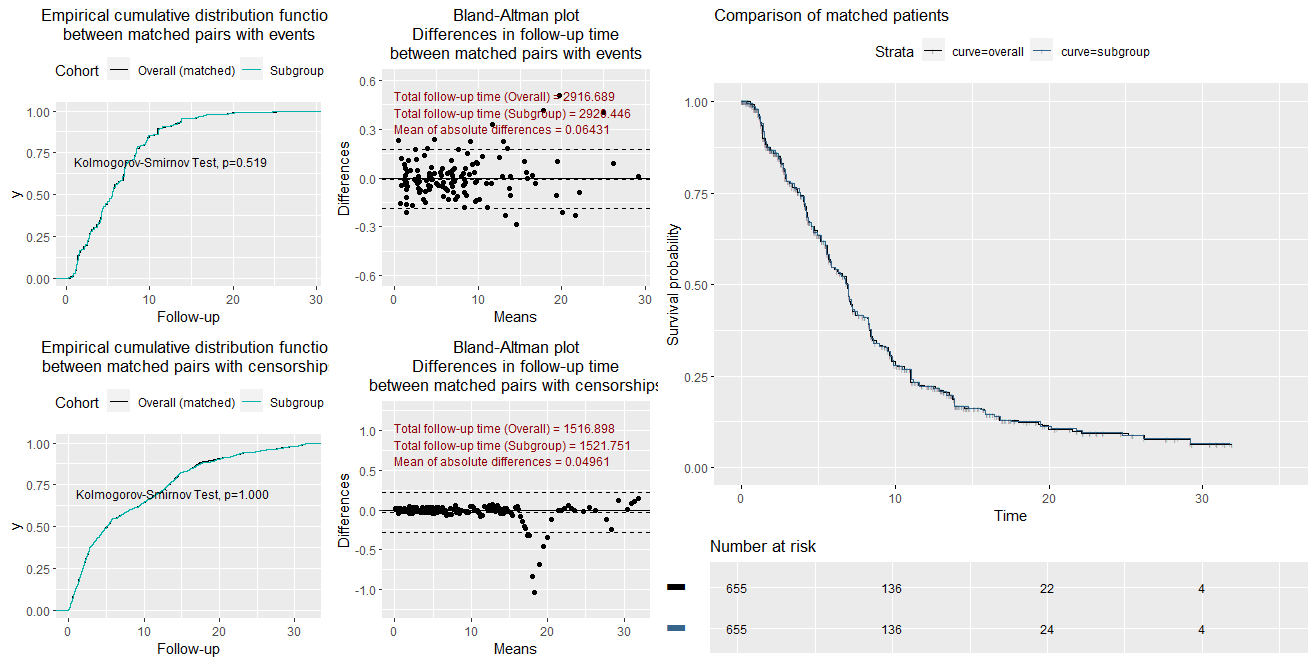 | |
| CheckMate-649: Asian patients | |
| Overall survival: CPS<5 for ICI plus chemotherapy  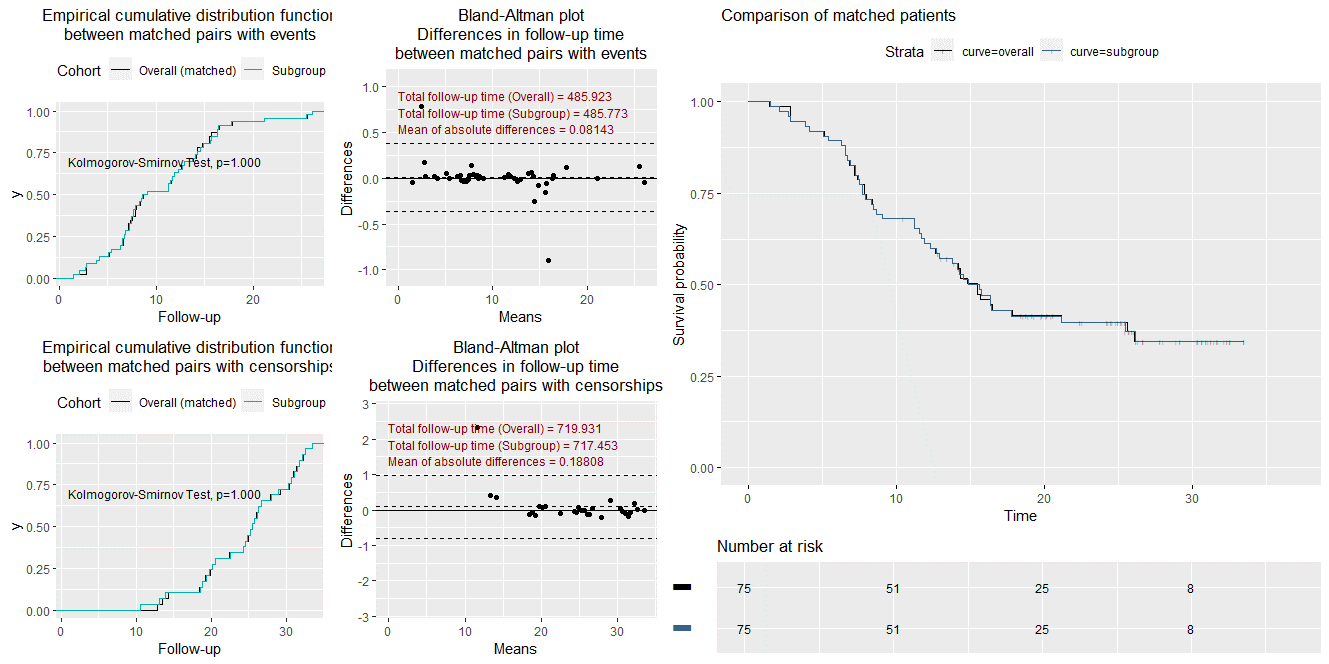 | |
| Overall survival: CPS<1 for ICI plus chemotherapy  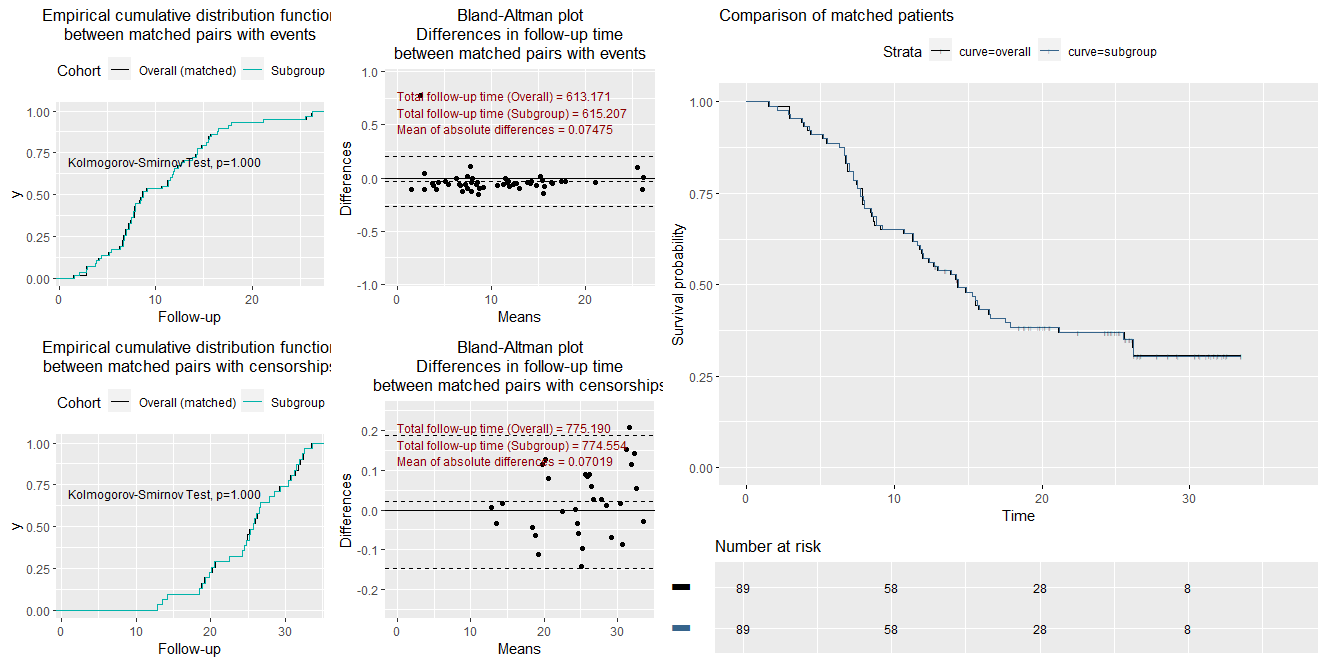 | |
| Overall survival: CPS<5 for chemotherapy  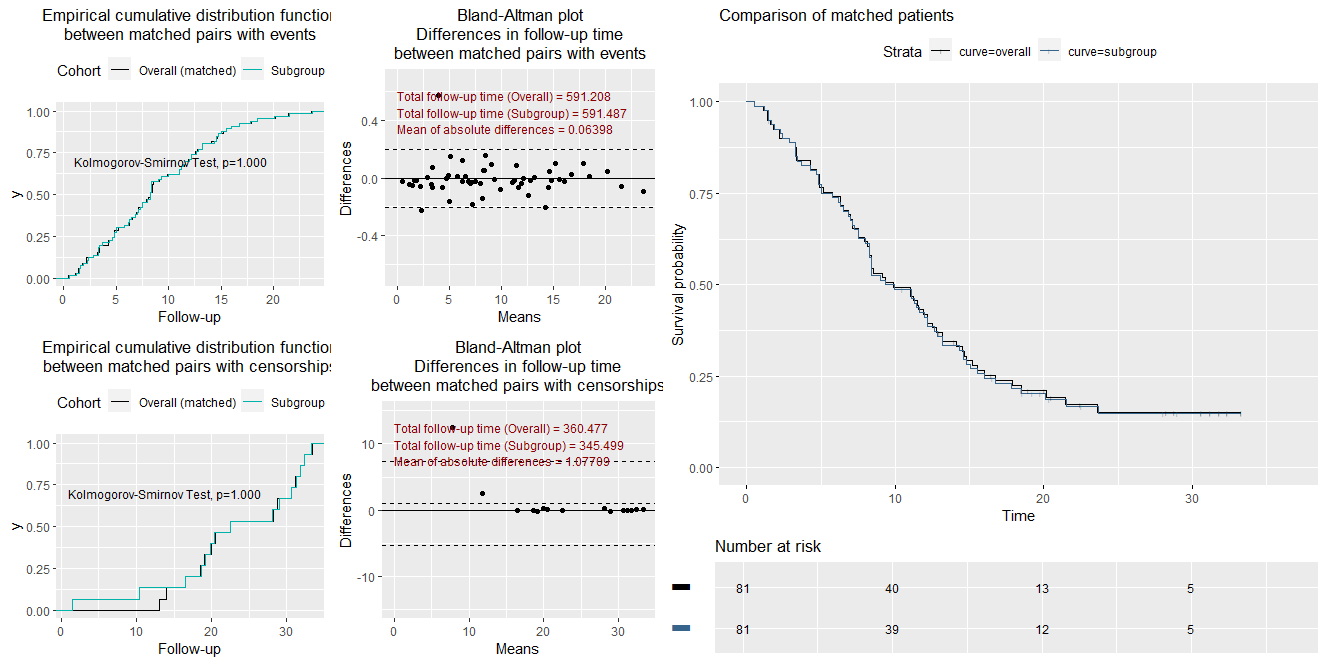 | |
| Overall survival: CPS<1 for chemotherapy  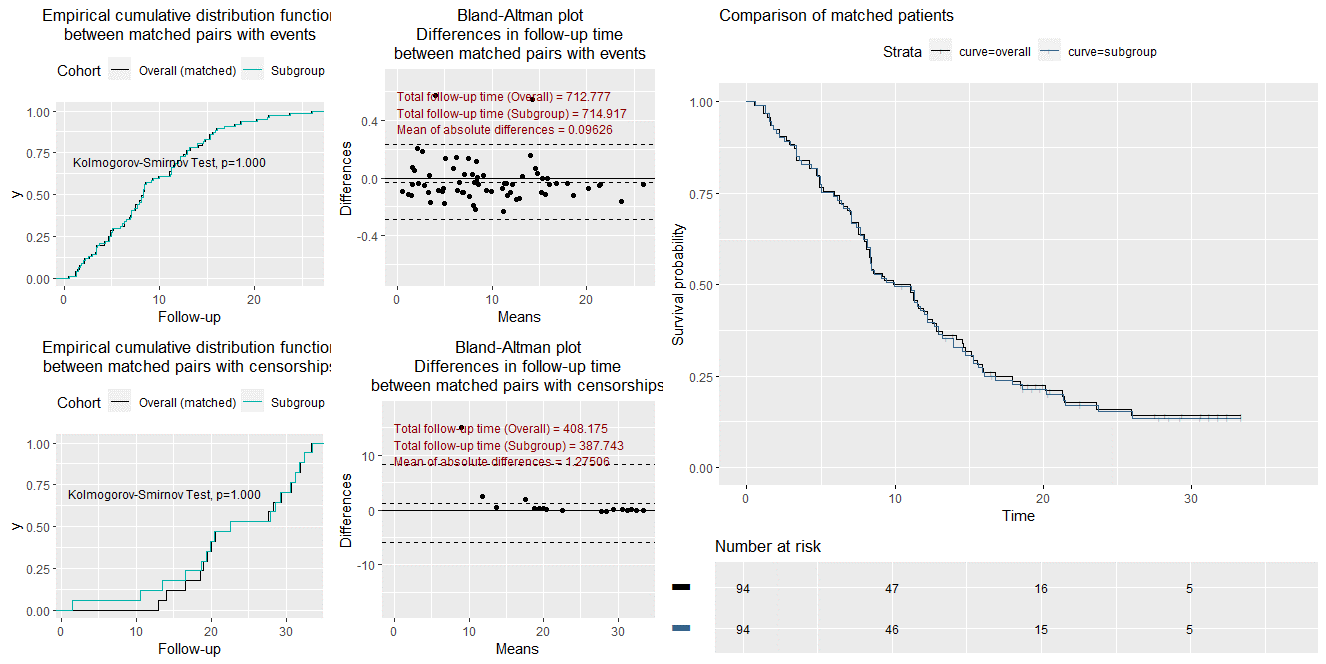 | |
| PFS: CPS<5 for ICI plus chemotherapy  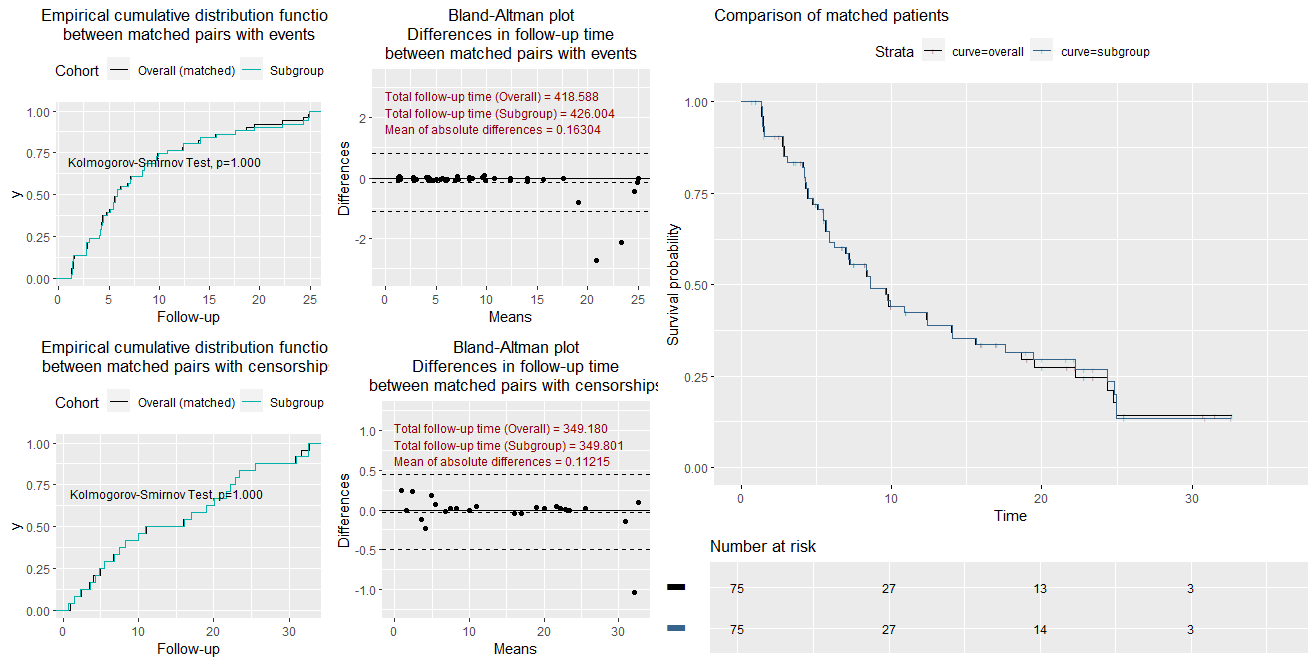 | |
| PFS: CPS<1 for ICI plus chemotherapy  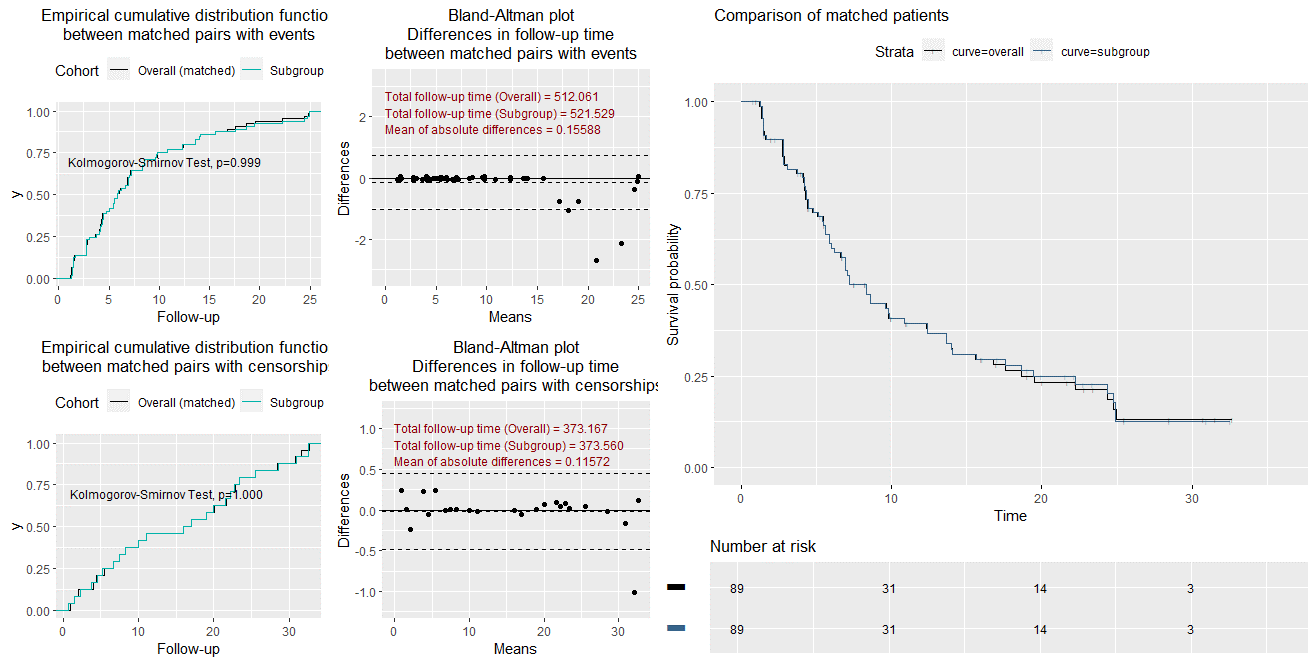 | |
| PFS: CPS<5 in the chemotherapy group  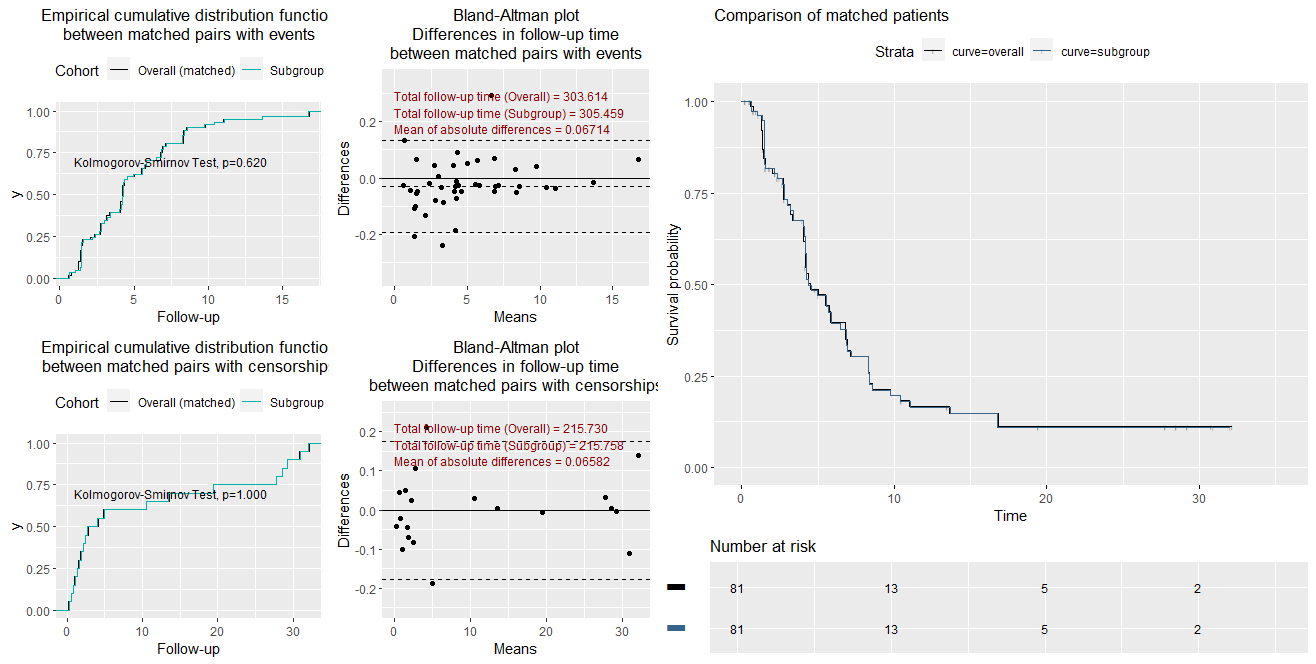 | |
| PFS: CPS<1 in the chemotherapy group  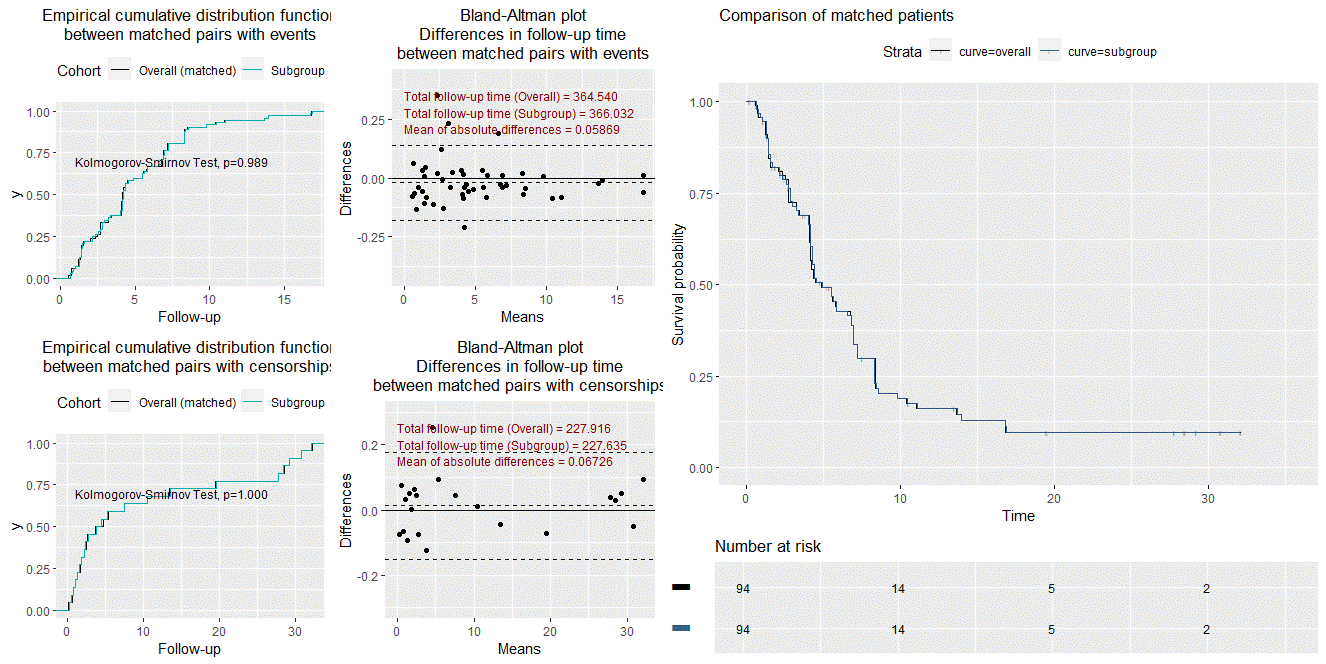 | |
| ORIENT-16 | |
| OS: CPS<5 for ICI plus chemotherapy  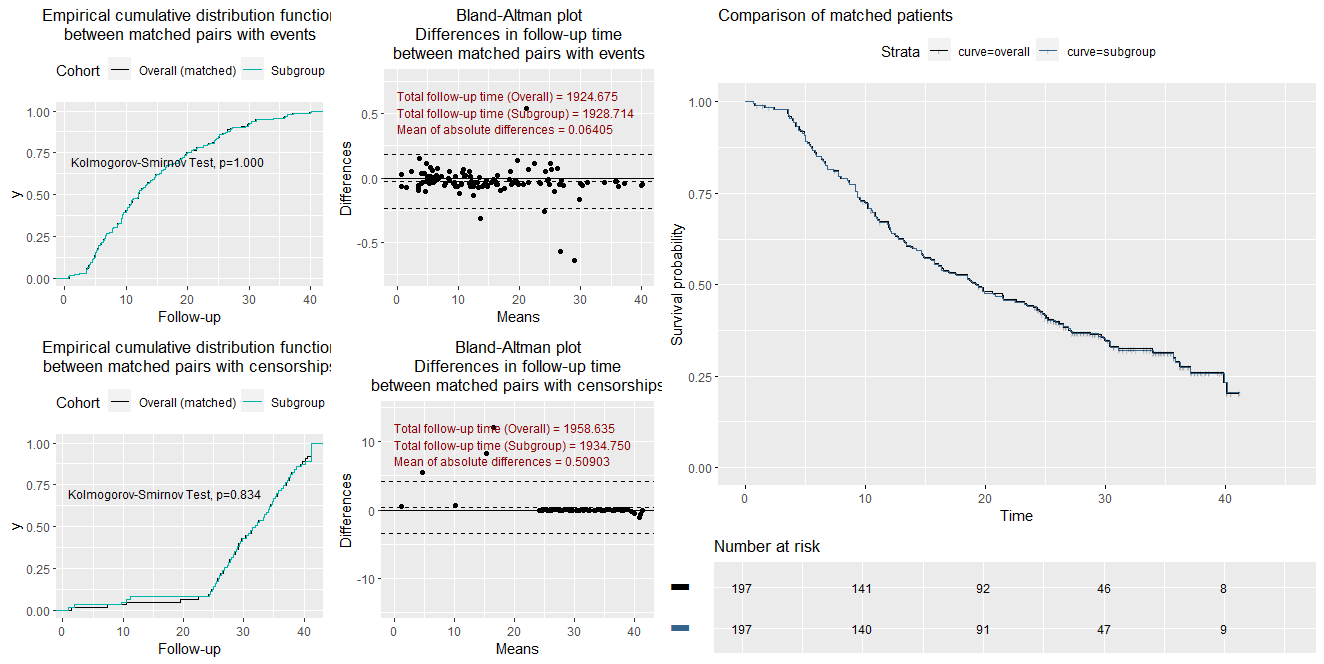 | |
| OS: CPS<5 for chemotherapy  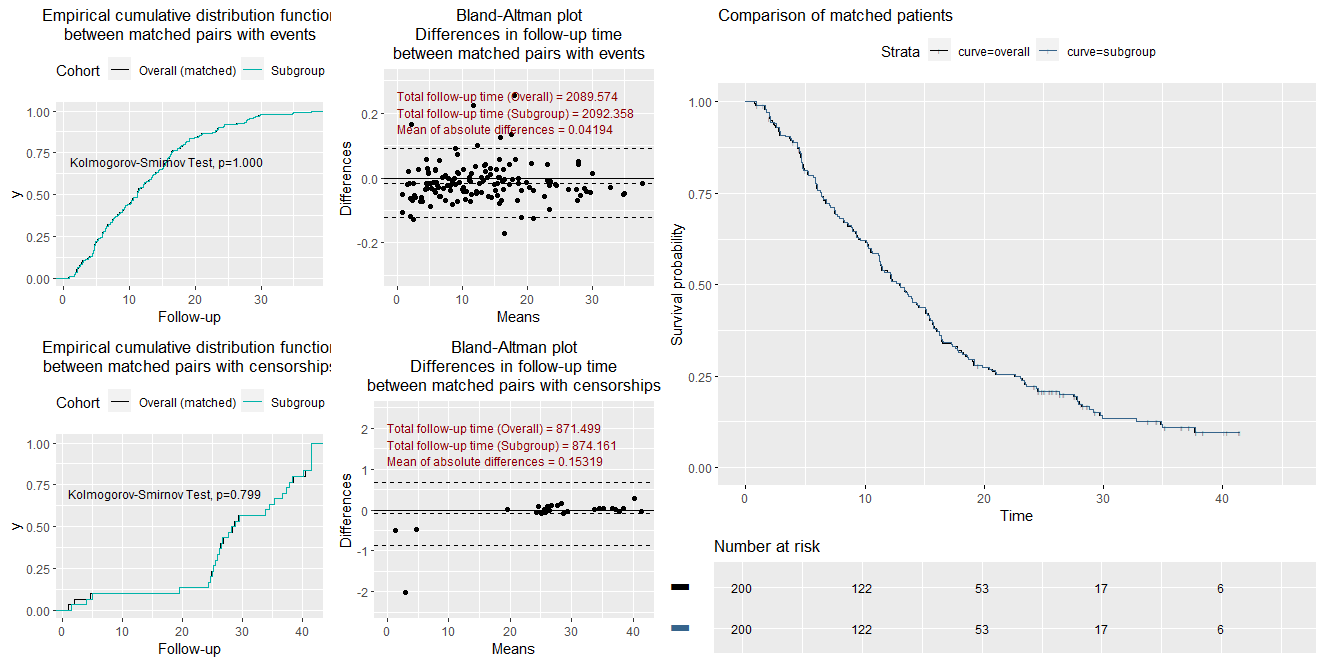 | |
| PFS: CPS<5 for ICI plus chemotherapy  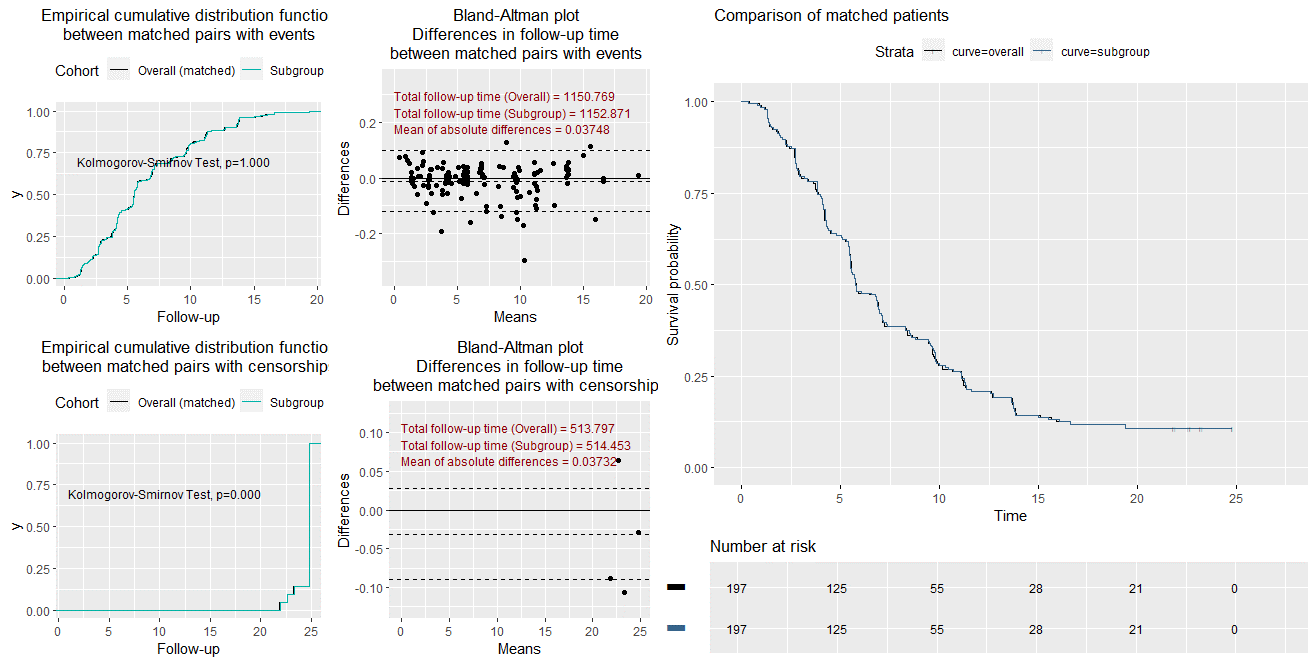 | |
| PFS: CPS<5 for chemotherapy  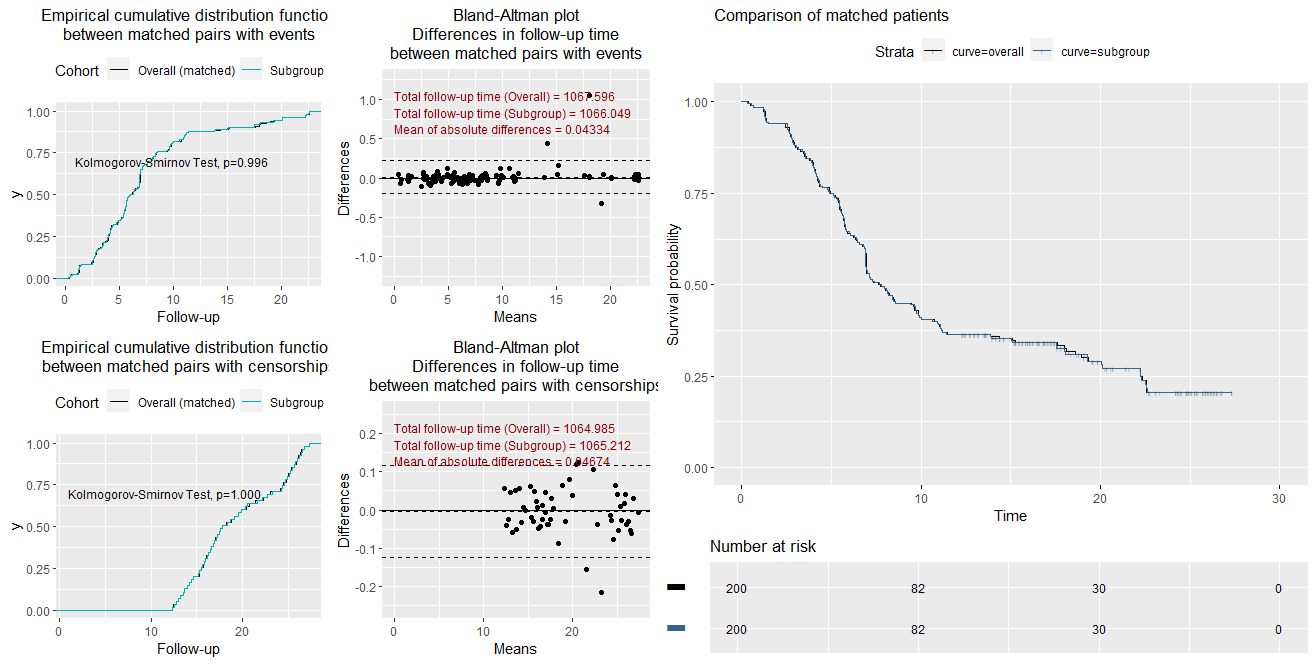 | |
| Keynote-062: global patients | |
| OS: CPS<10 for ICI plus chemotherapy  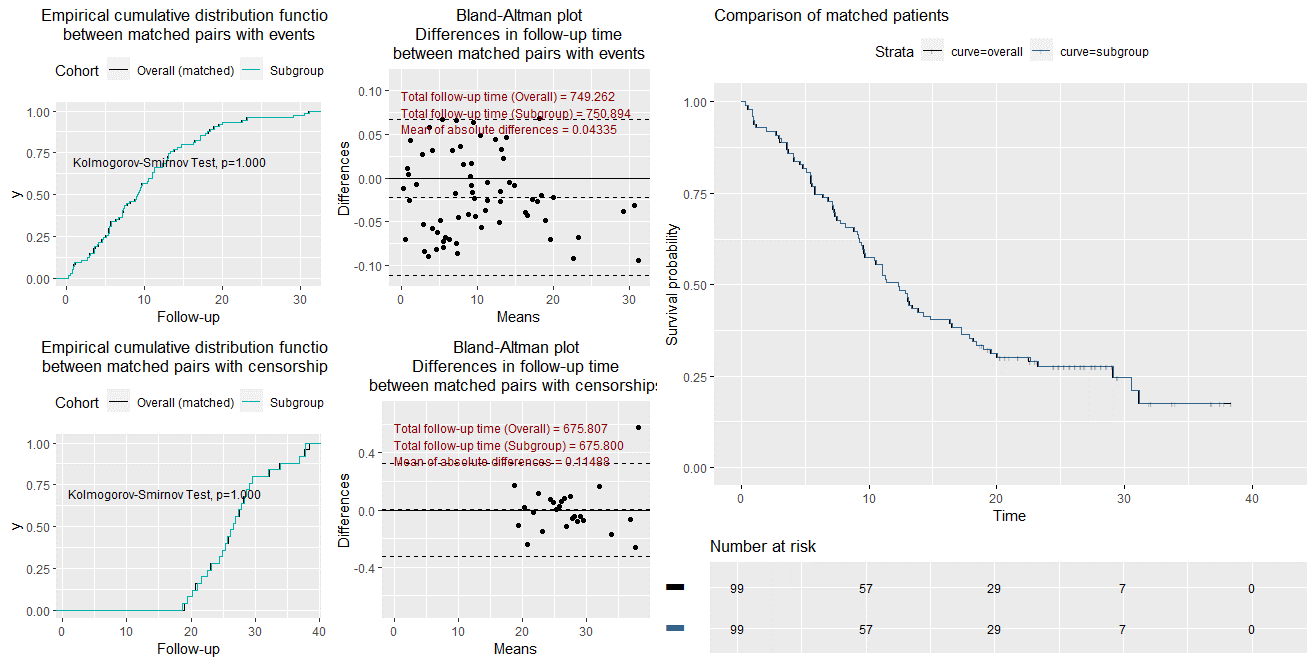 | |
| OS: CPS<10 for chemotherapy  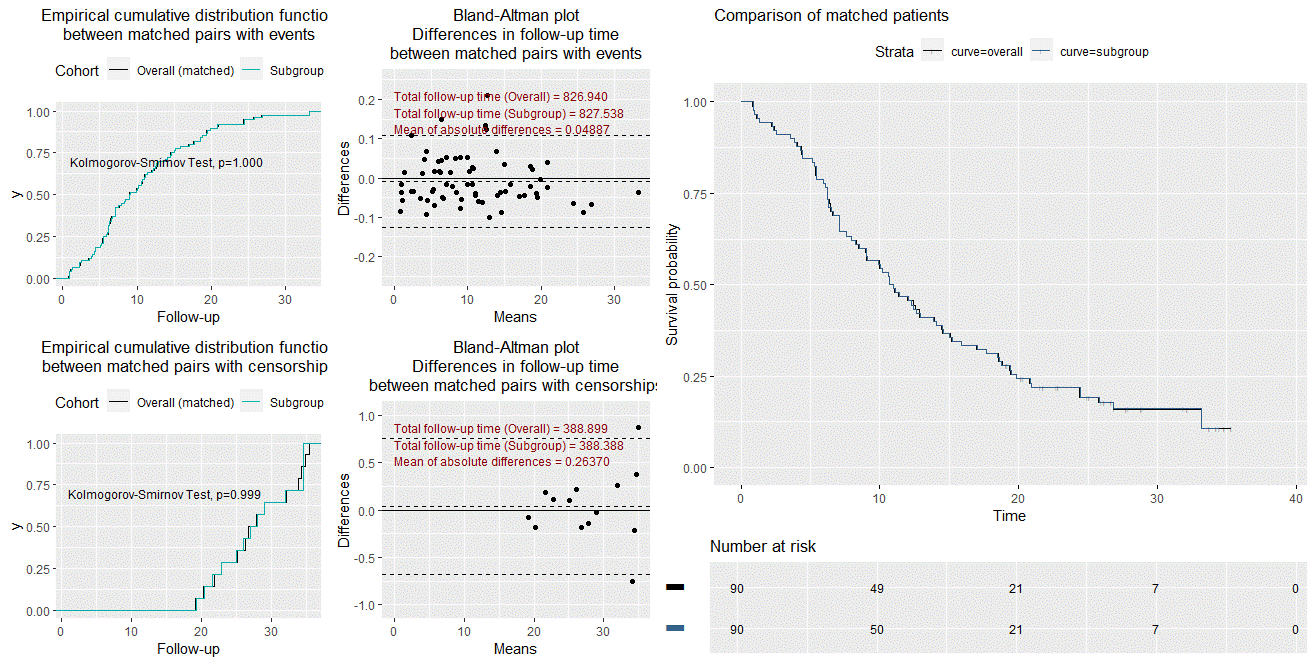 | |
| PFS: CPS<10 for ICI plus chemotherapy  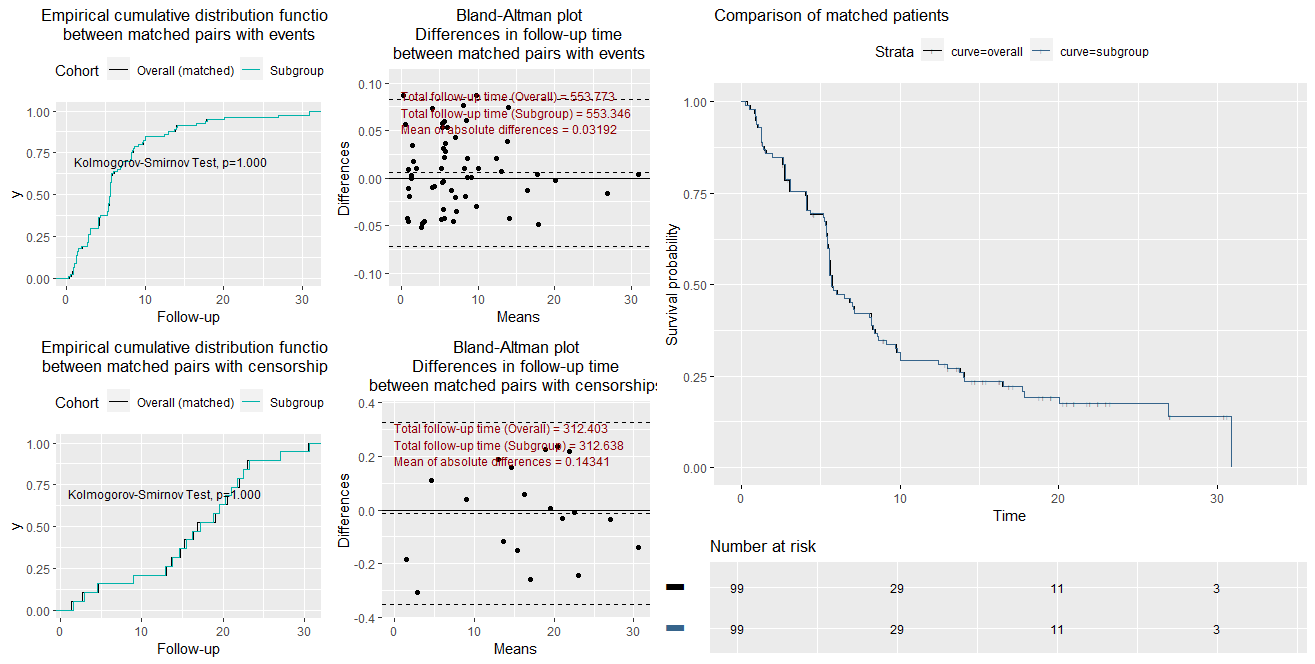 | |
| PFS: CPS<10 for chemotherapy  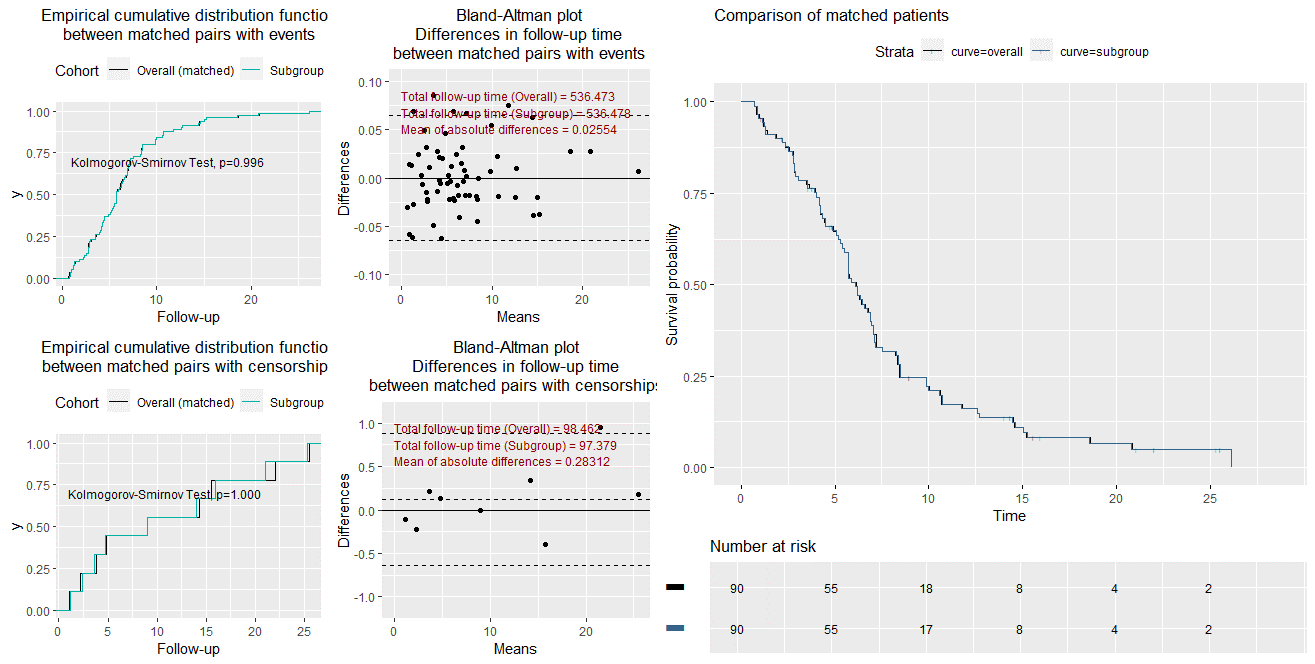 | |
| Keynote-062: Asian patients | |
| OS: CPS<10 for ICI plus chemotherapy  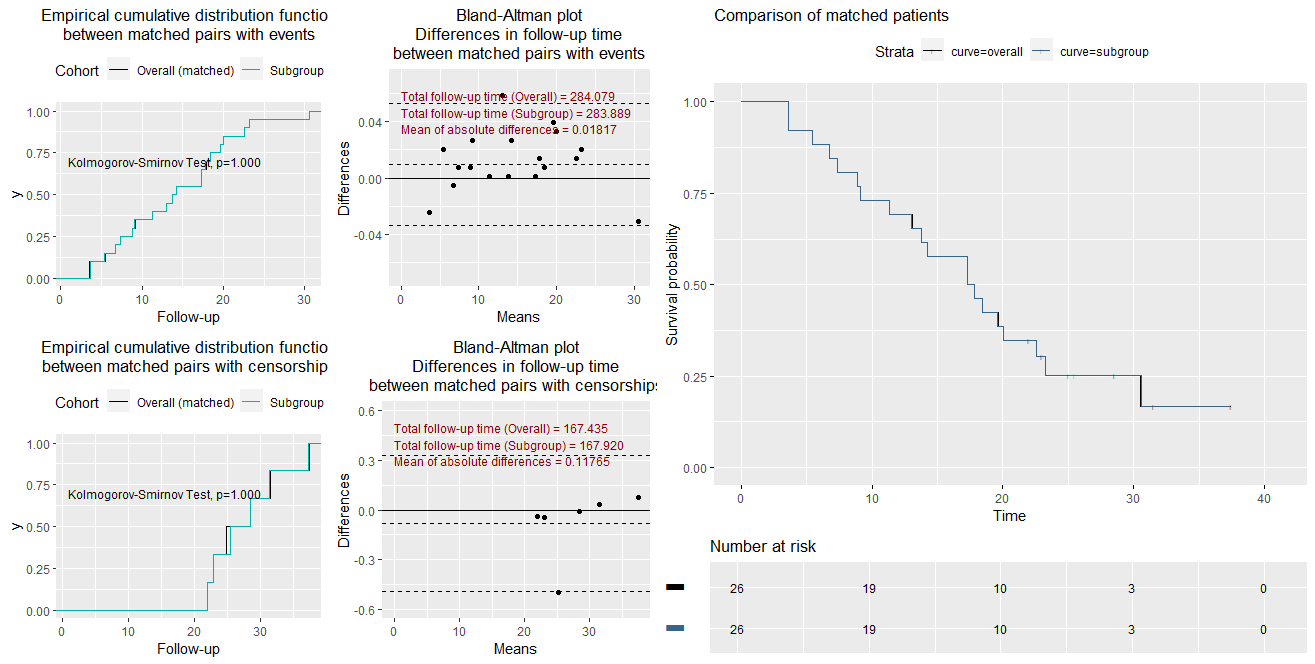 | |
| OS: CPS<10 for chemotherapy  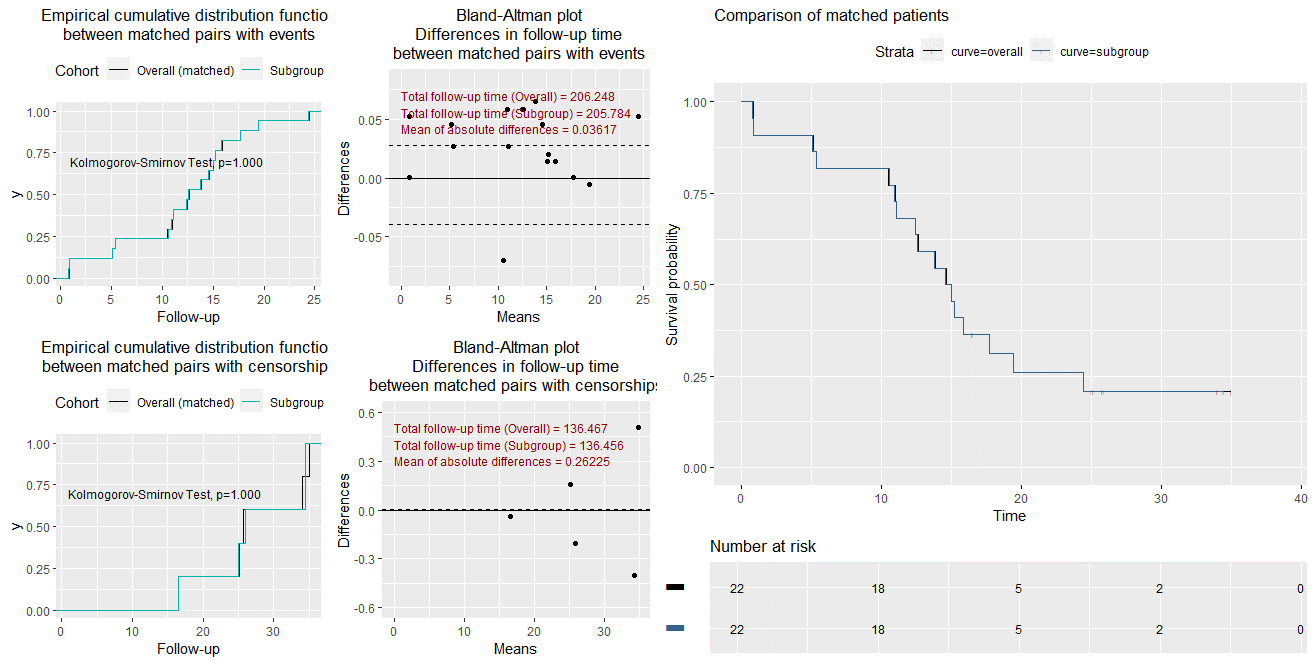 | |
| PFS: CPS<10 for ICI plus chemotherapy  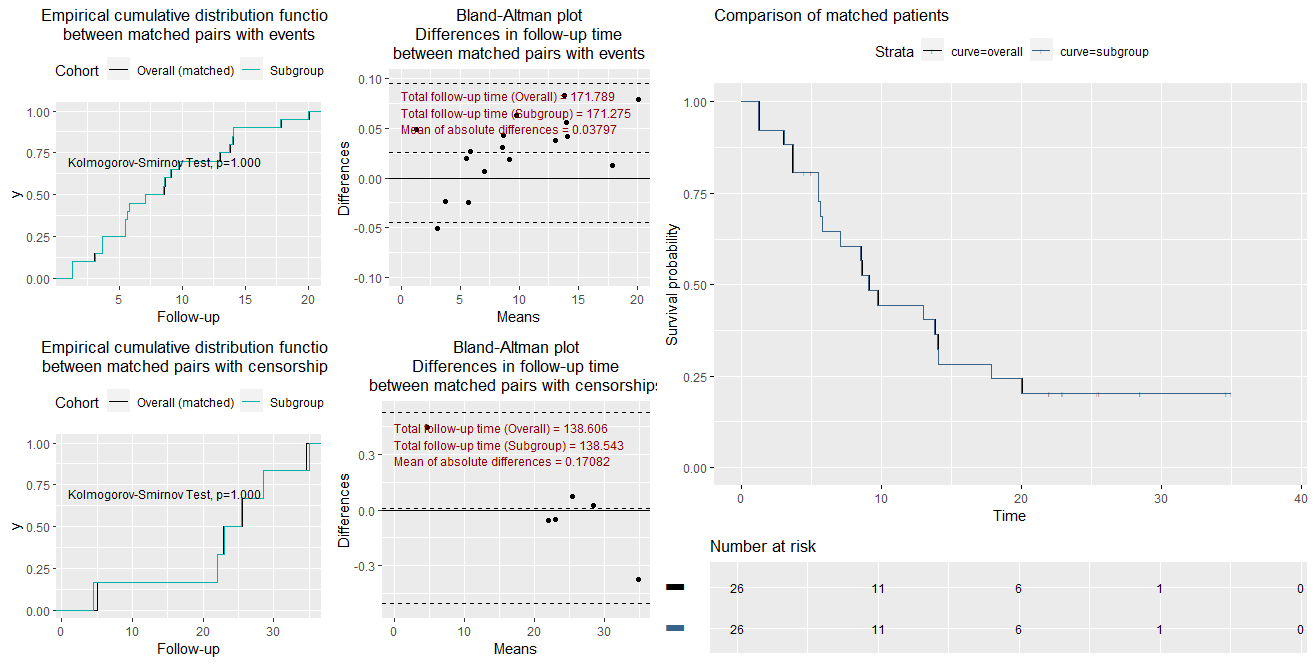 | |
| PFS: CPS<10 for chemotherapy  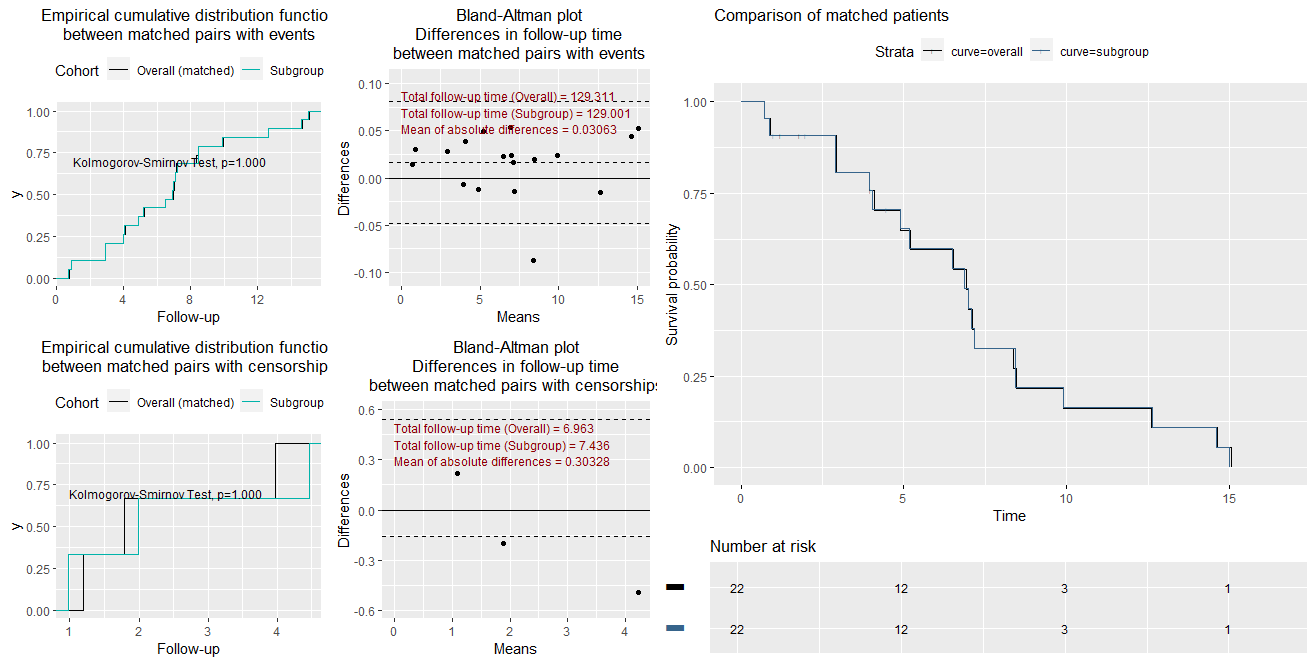 | |
| Keynote-590 | |
| OS: all patients with [adenocarcinoma](javascript:;) in the ICI plus chemotherapy group  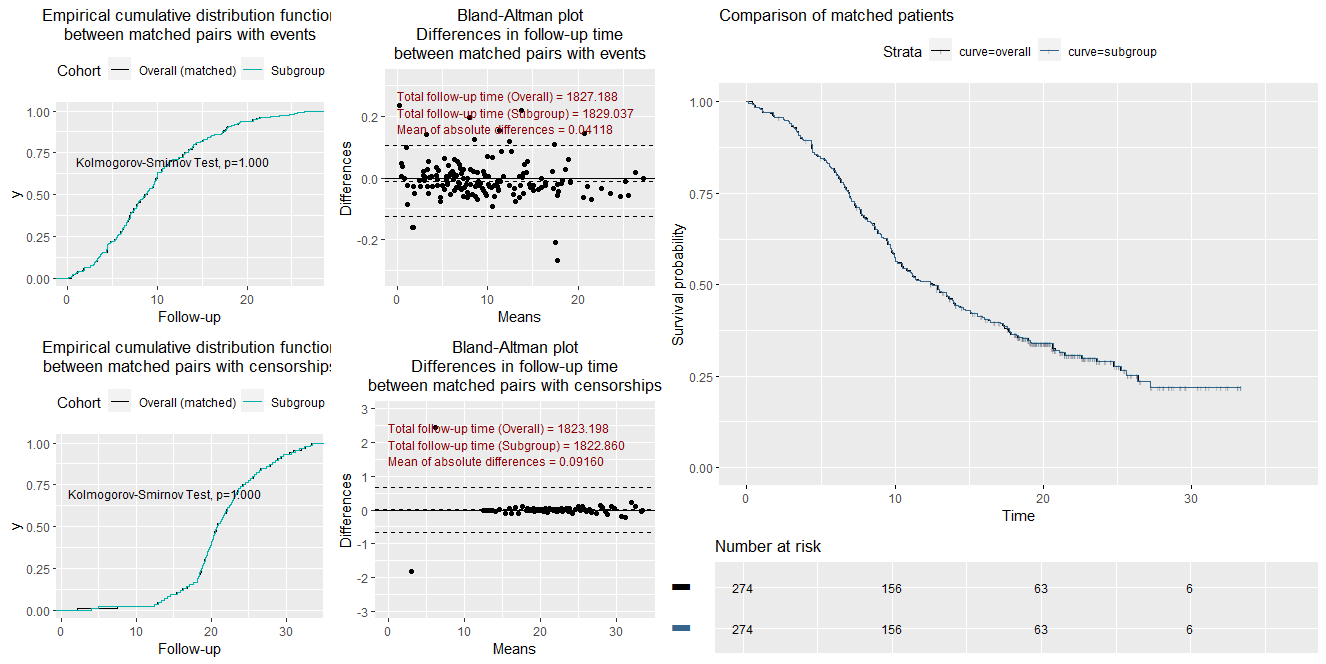 | |
| OS: CPS≥10 in the ICI plus chemotherapy group  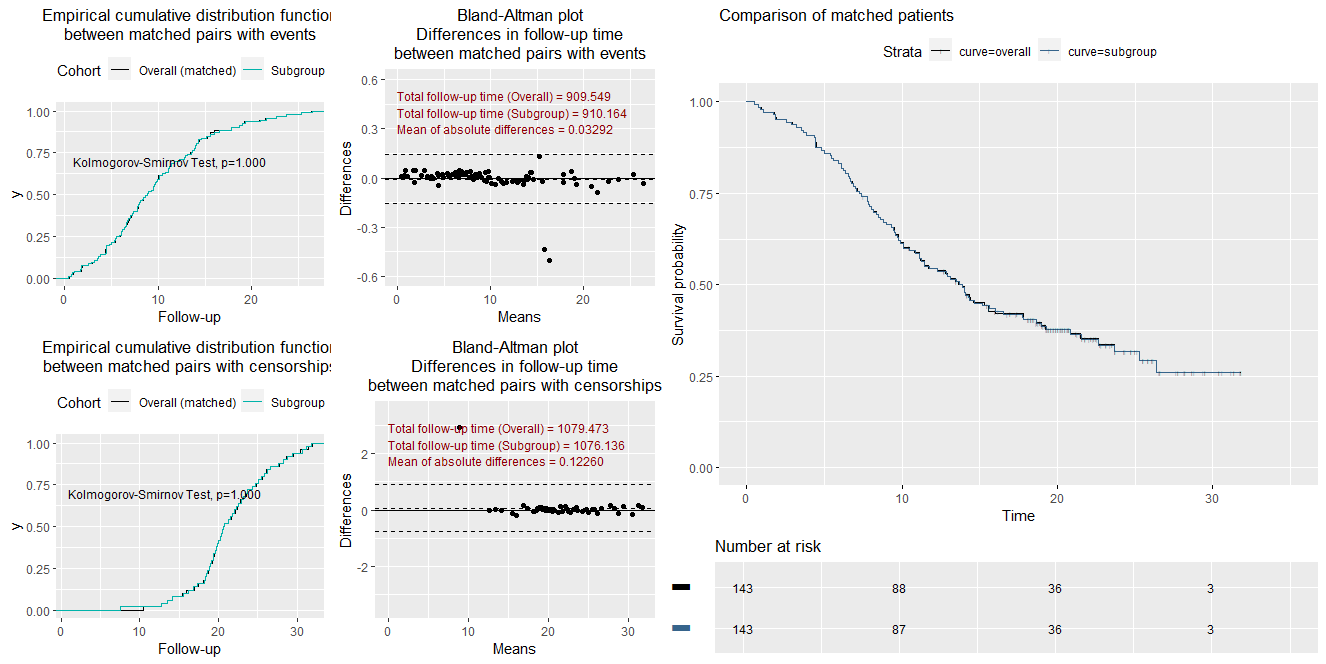 | |
| OS: CPS<10 in the ICI plus chemotherapy group  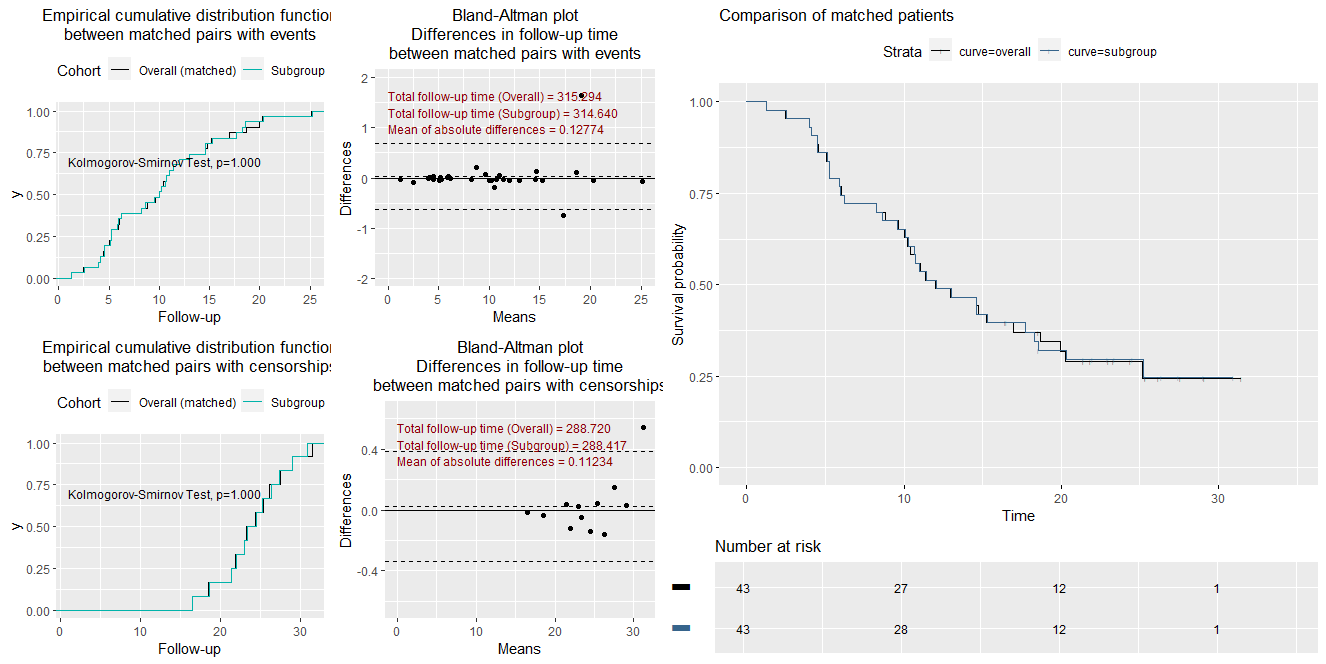 | |
| OS: all patients with [adenocarcinoma](javascript:;) in the chemotherapy group  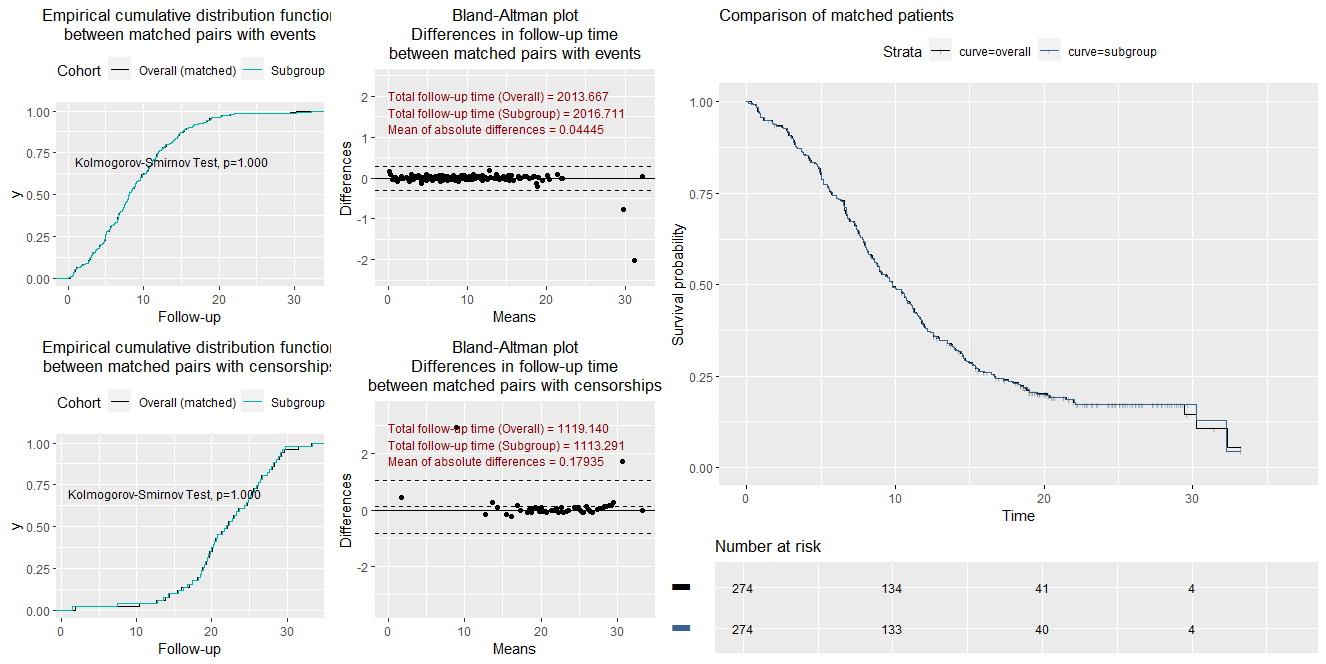 | |
| OS: CPS≥10 in the chemotherapy group  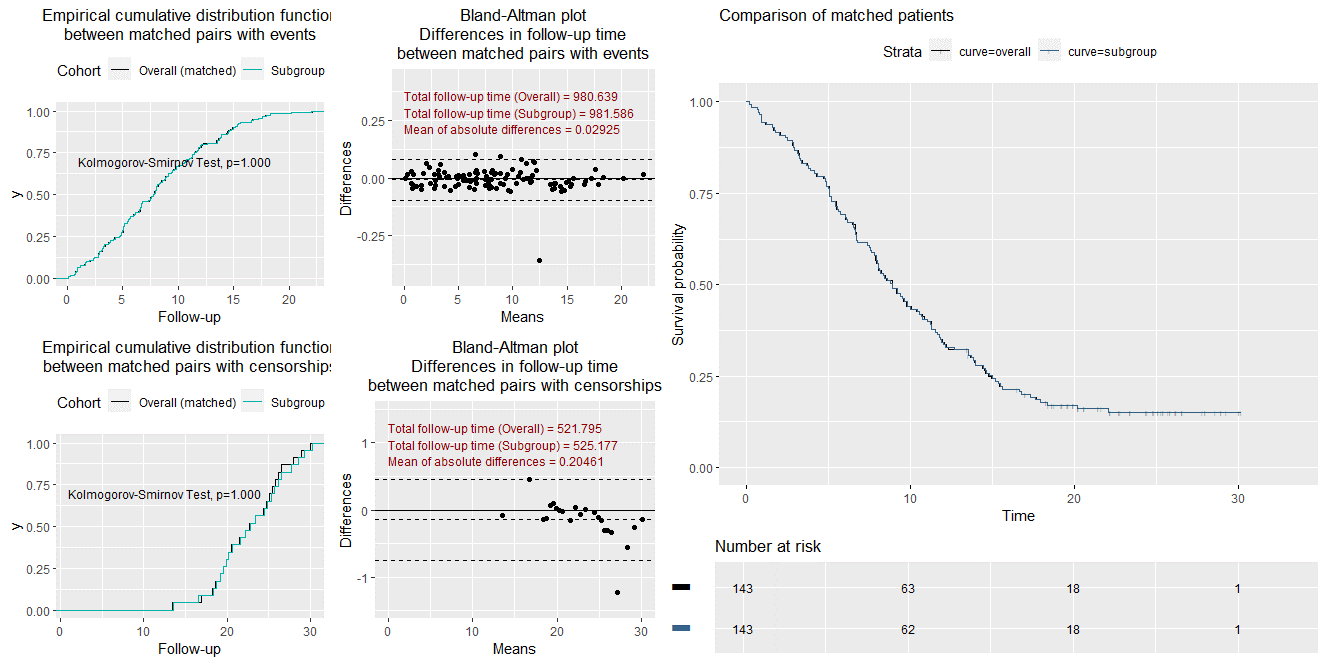 | |
| OS: CPS<10 in the chemotherapy group  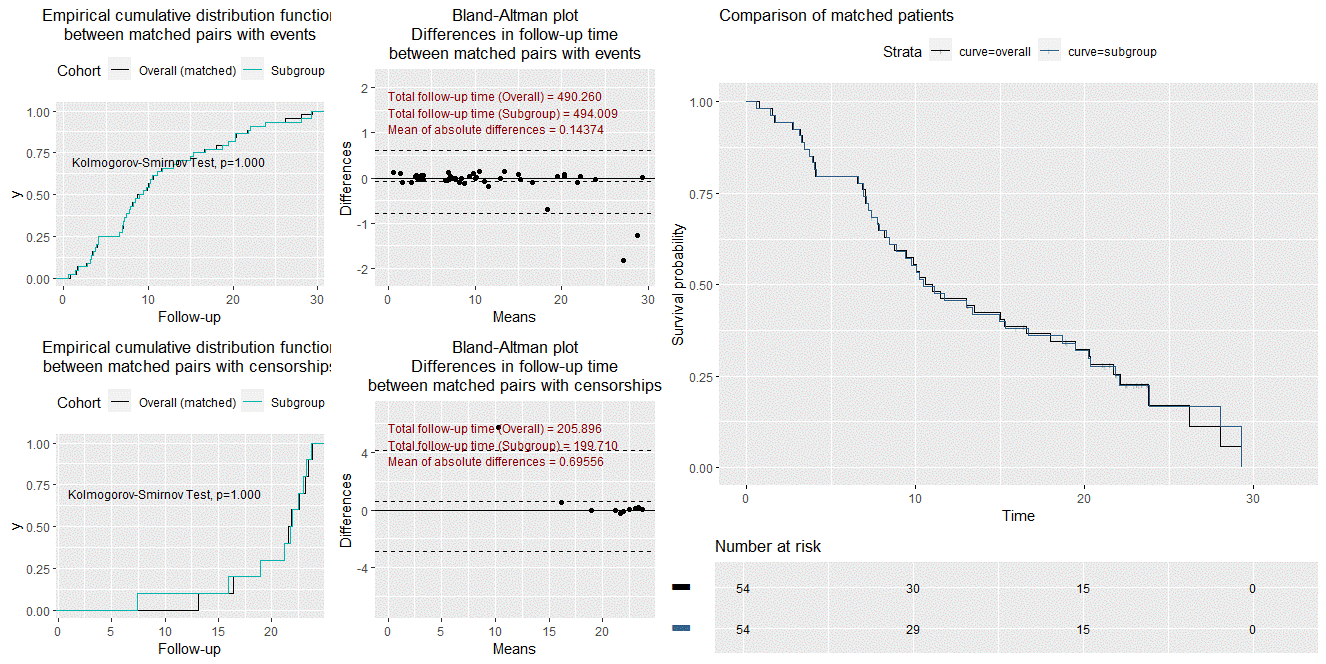 | |
| PFS: all patients with [adenocarcinoma](javascript:;) in the ICI plus chemotherapy group  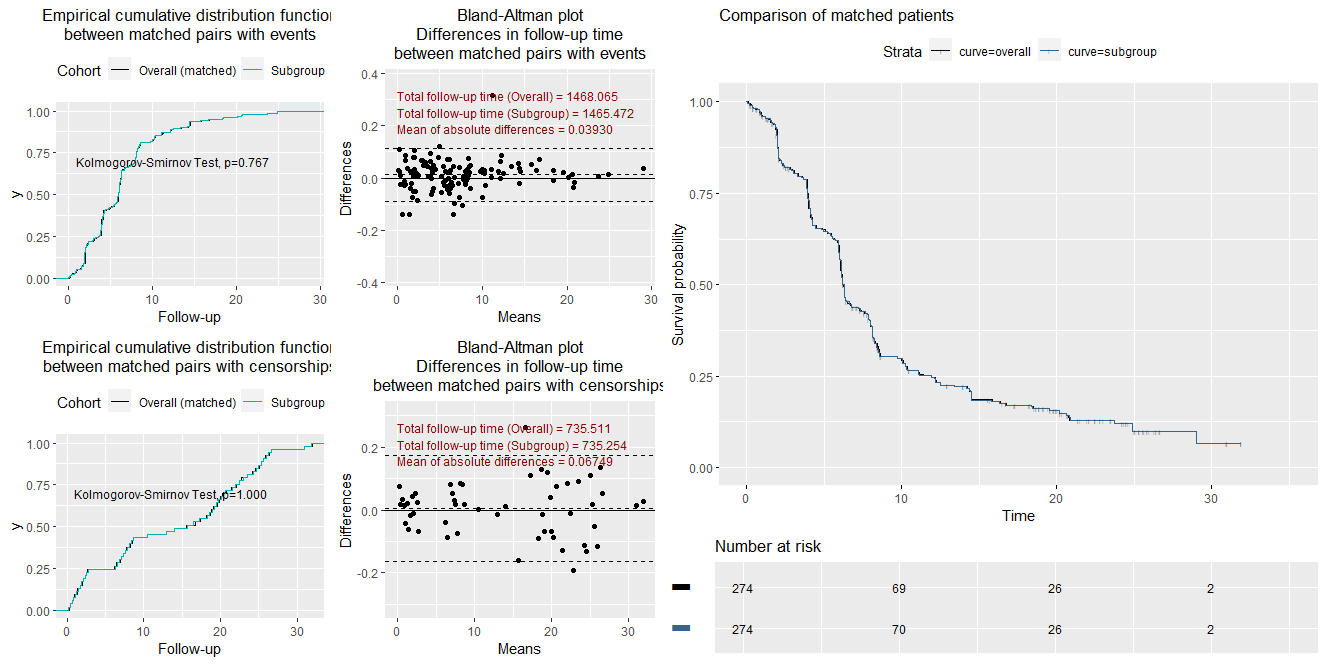 | |
| PFS: all patients with [adenocarcinoma](javascript:;) in the chemotherapy group  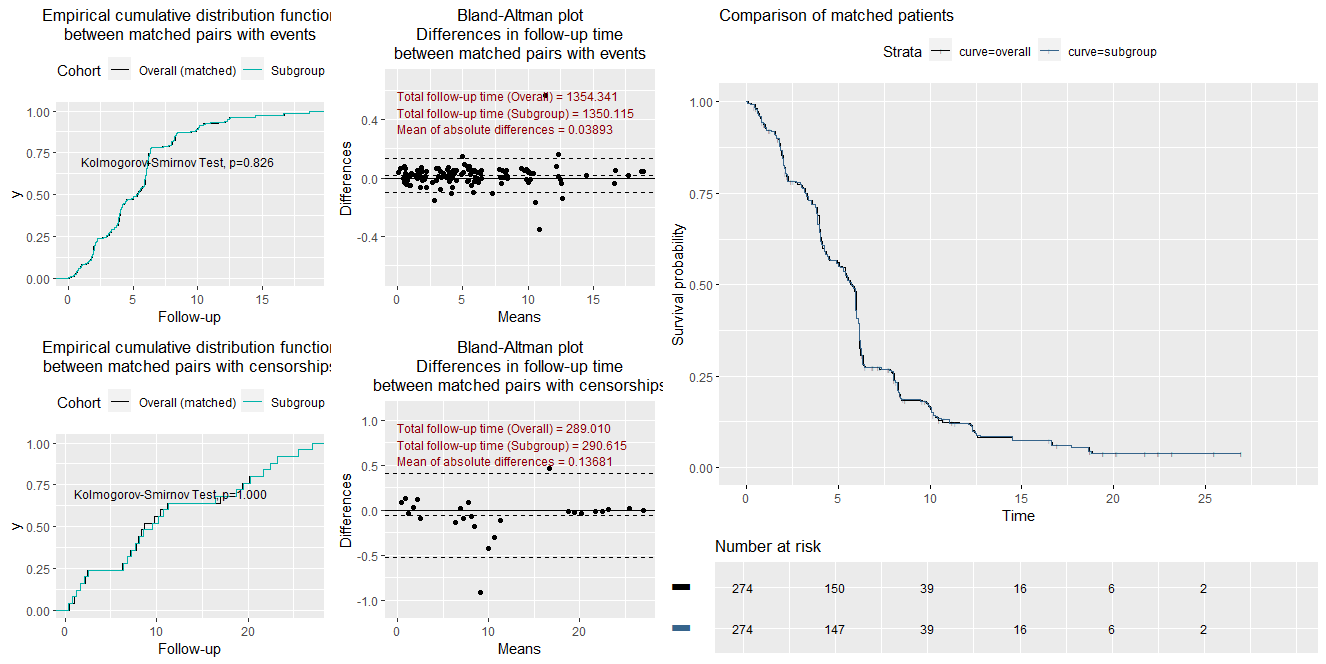 | |

# Table S8. Convergence plots and histograms of simulations

| Histogram of ln(HR)  CM-649 global patients, OS, ICI+CHEMO, CPS<5  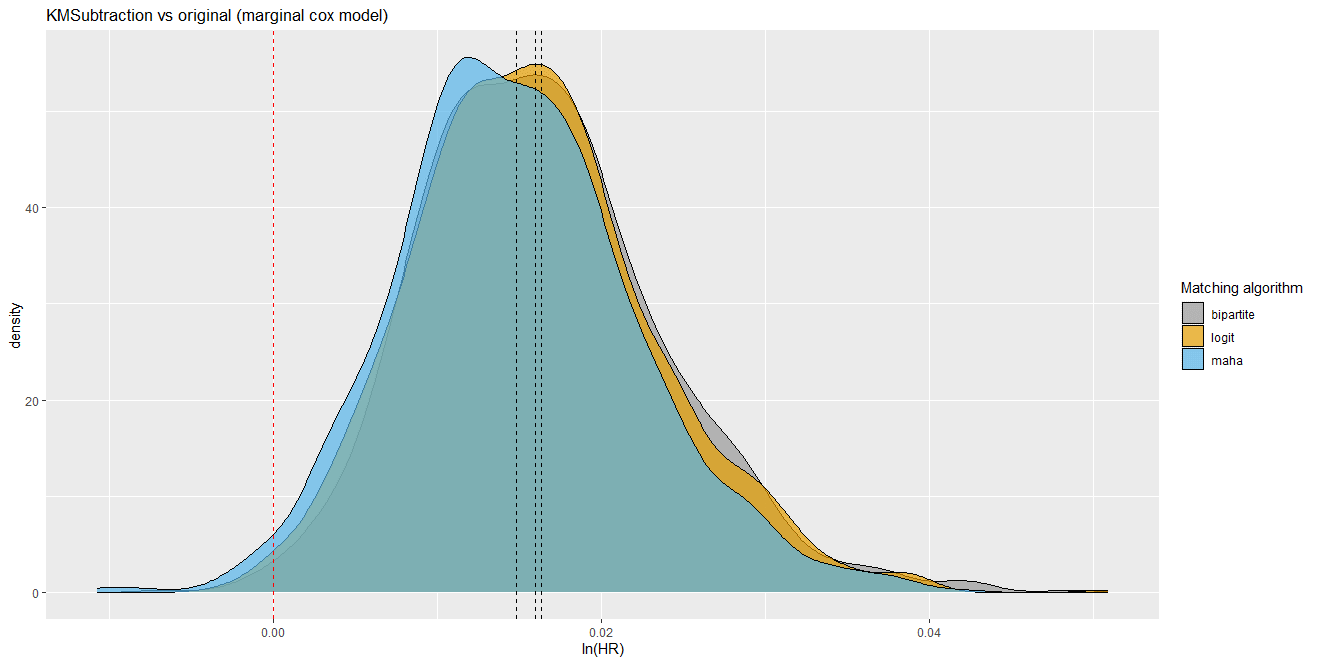 | Histogram of \|ln(HR)\|  CM-649 global patients, OS, ICI+CHEMO, CPS<5  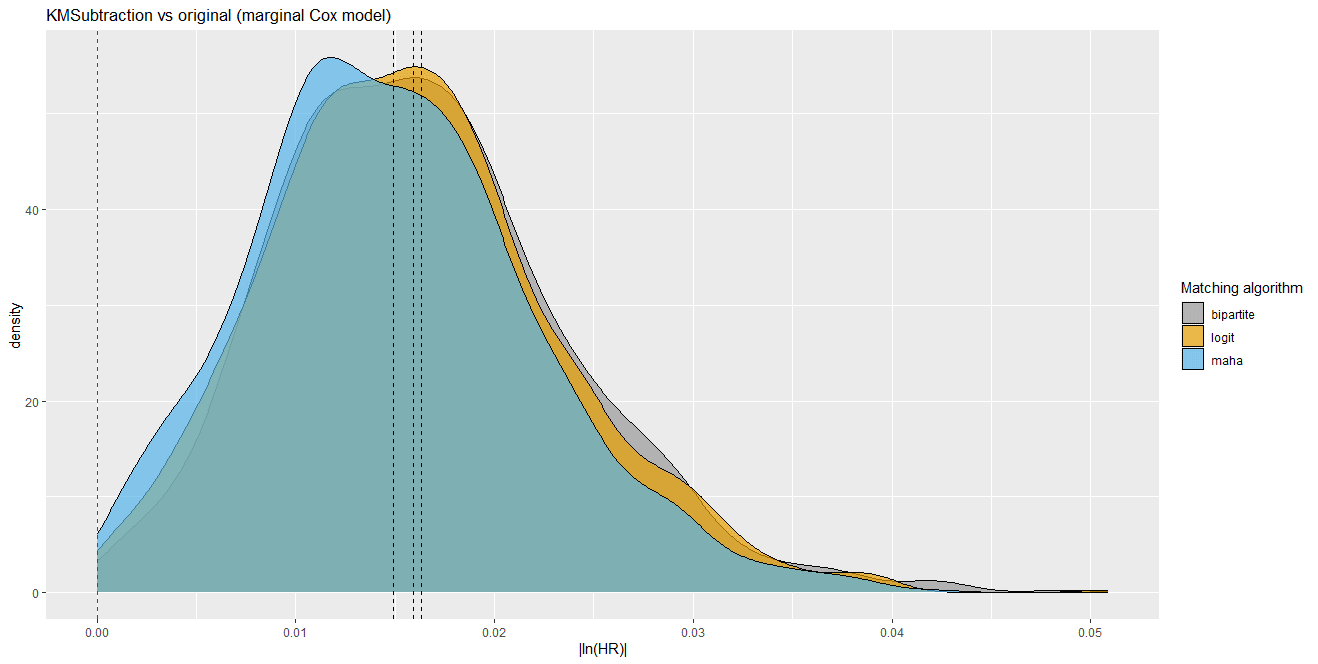 | | Convergence plot  CM-649 global patients, OS, ICI+CHEMO, CPS<5  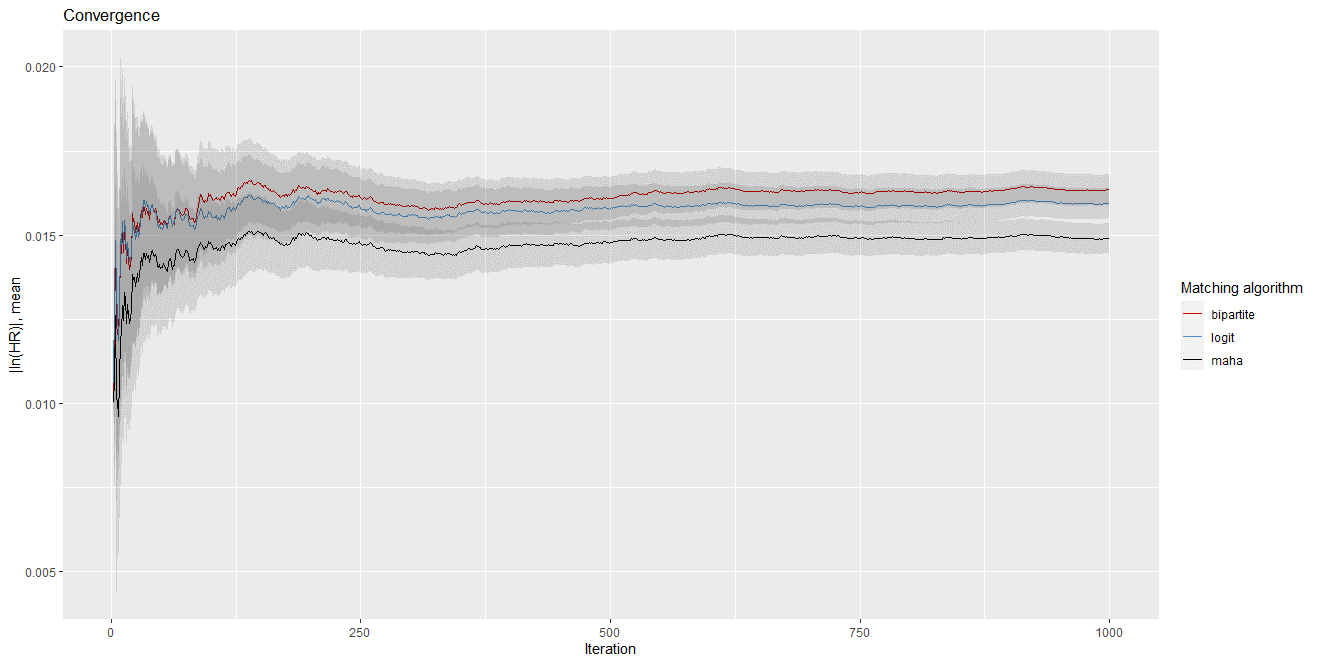 |
| --- | --- | --- | --- |
| Histogram of ln(HR)  CM-649 global patients, OS, CHEMO, CPS<5  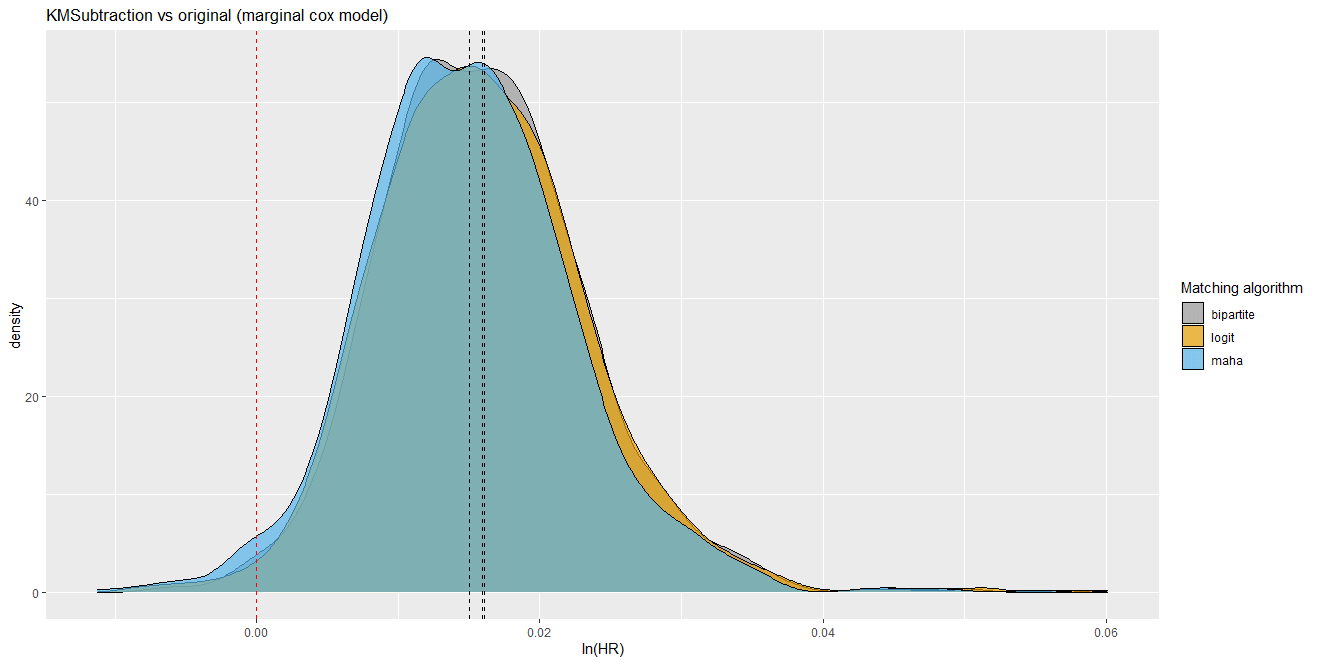 | Histogram of \|ln(HR)\|  CM-649 global patients, OS, CHEMO, CPS<5  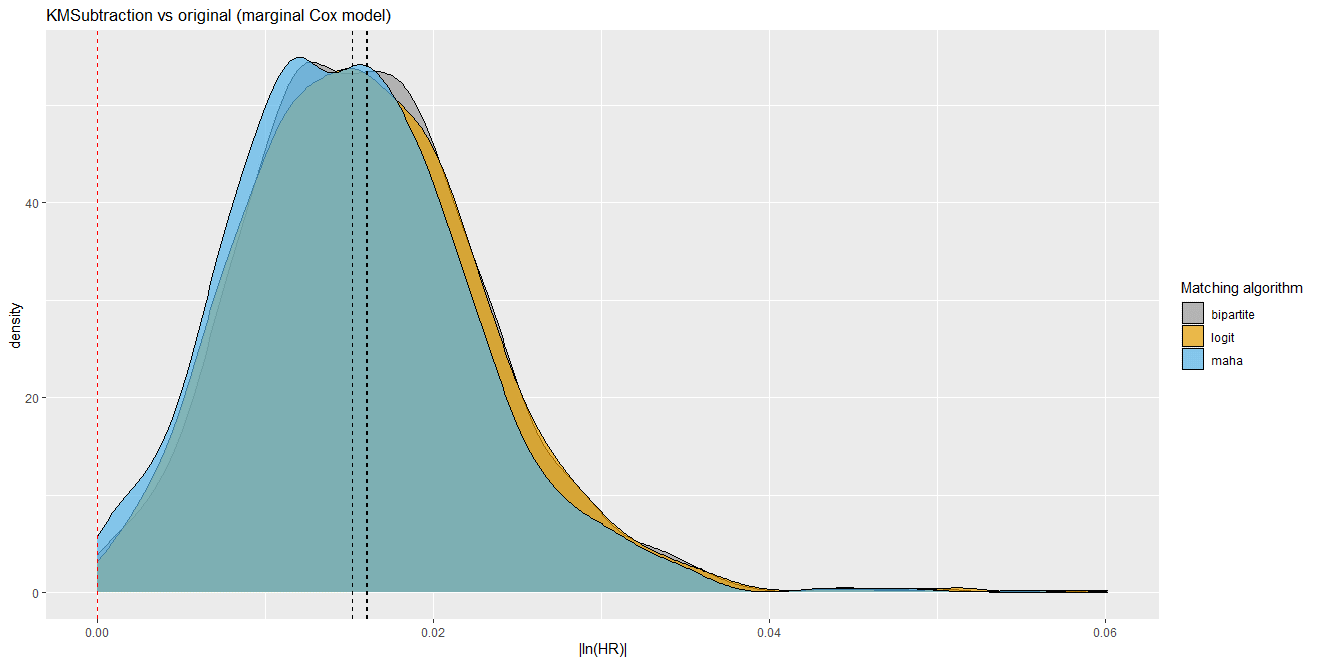 | | Convergence plot  CM-649 global patients, OS, CHEMO, CPS<5  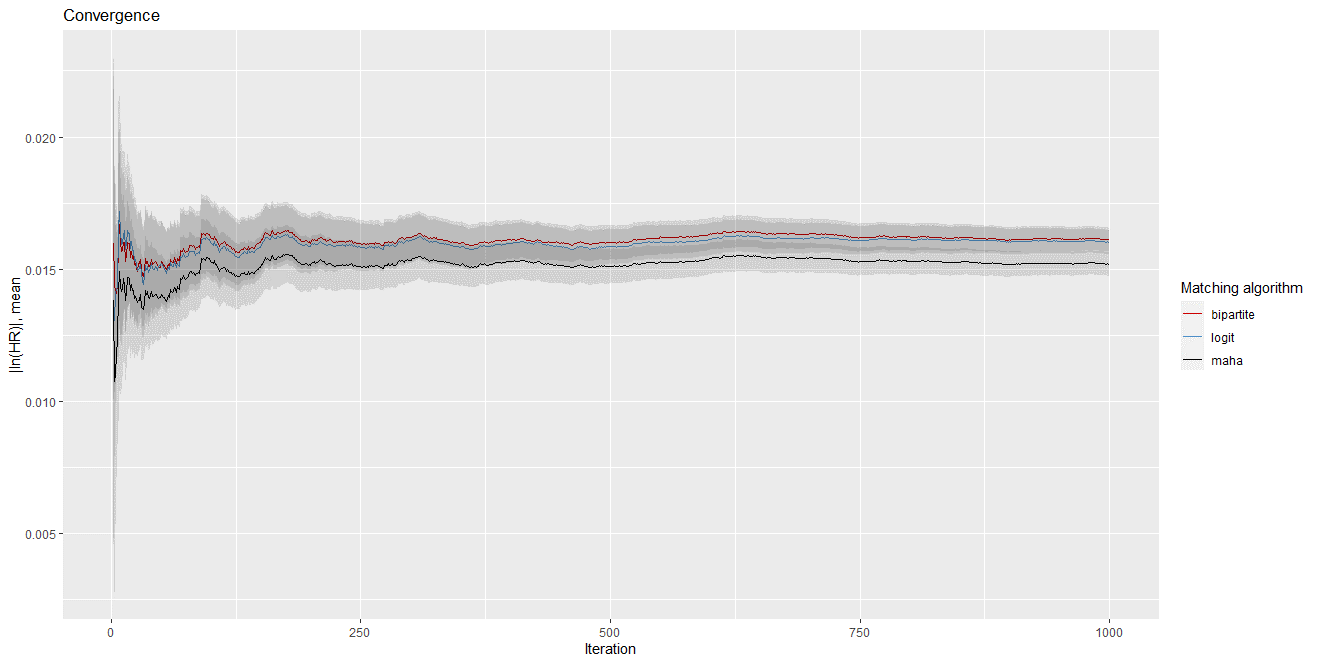 |
| Histogram of ln(HR)  CM-649 global patients, OS, ICI+CHEMO, CPS<1  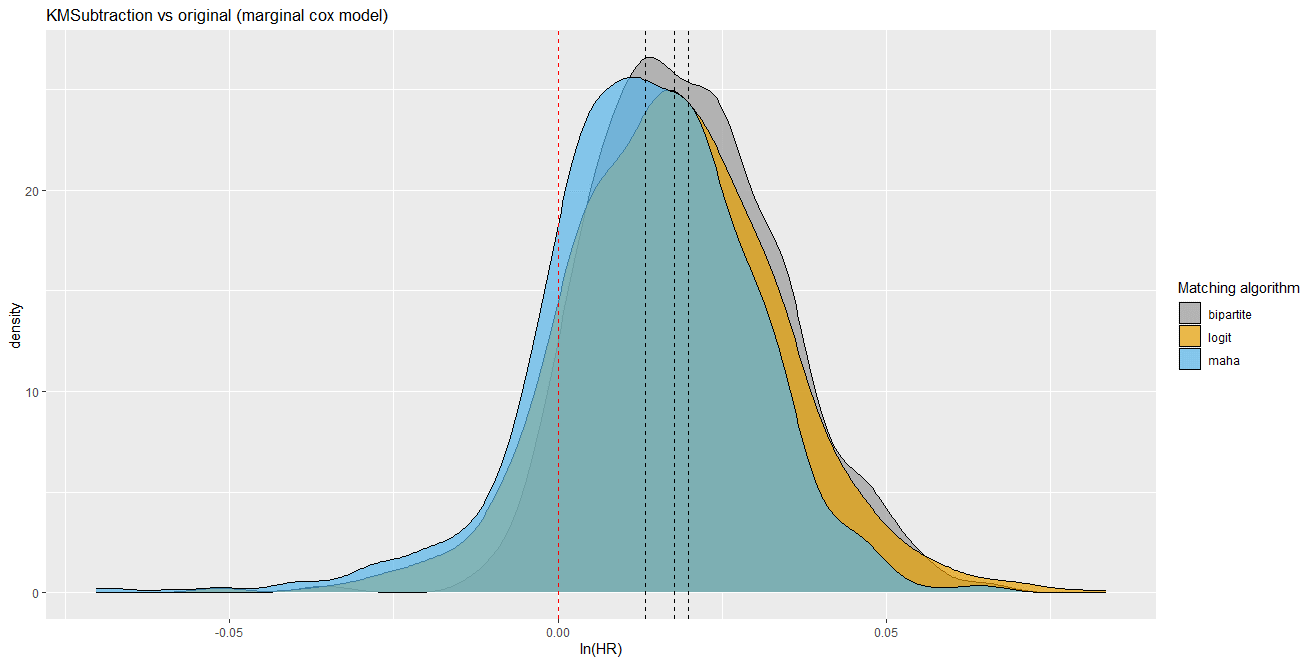 | Histogram of \|ln(HR)\|  CM-649 global patients, OS, ICI+CHEMO, CPS<1  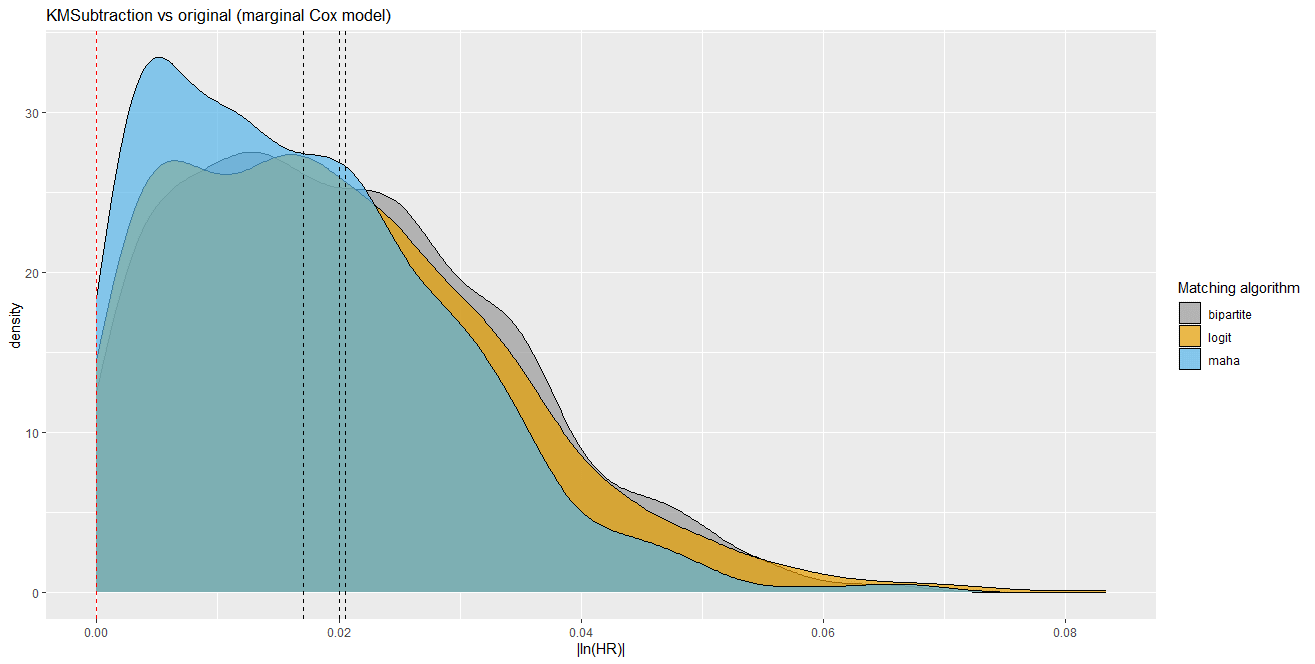 | | Convergence plot  CM-649 global patients, OS, ICI+CHEMO, CPS<1  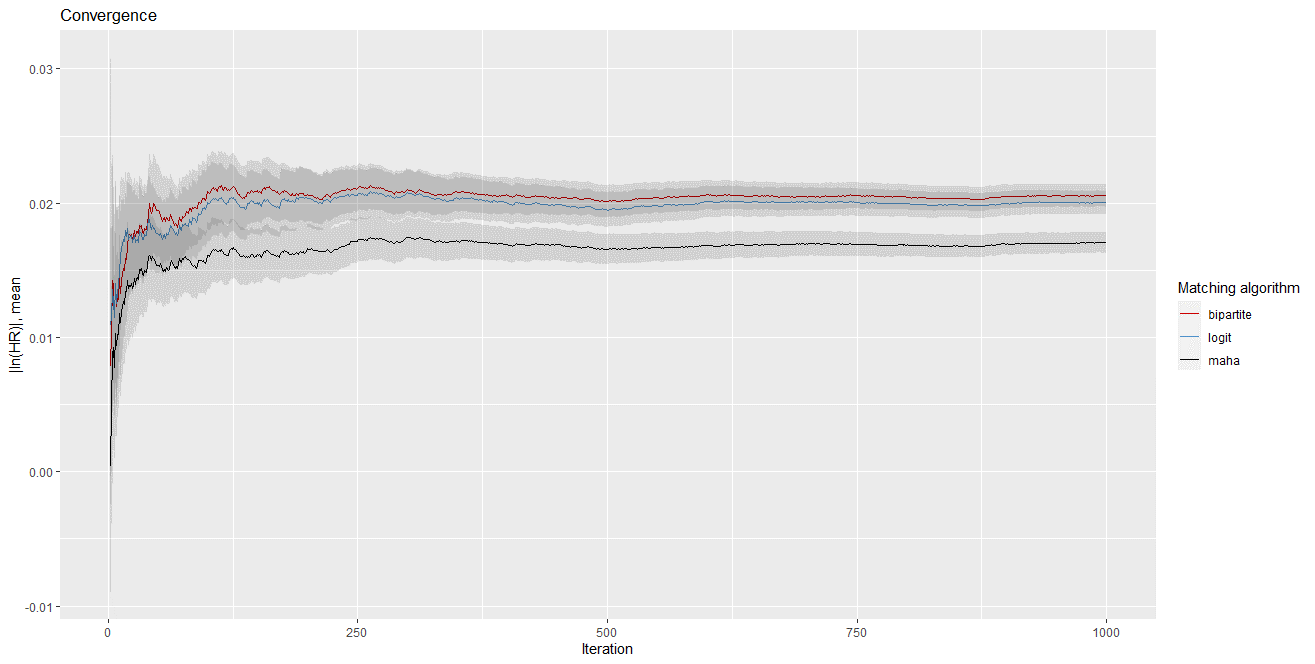 |
| Histogram of ln(HR)  CM-649 global patients, OS, CHEMO, CPS<1  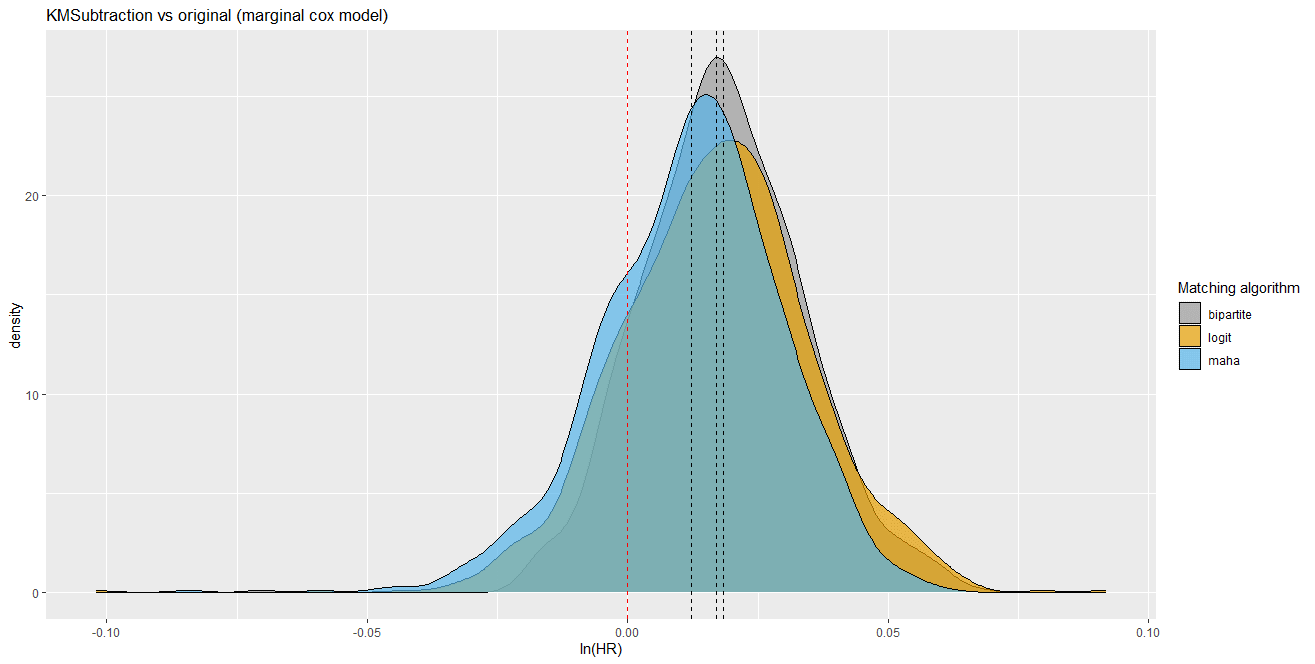 | Histogram of \|ln(HR)\|  CM-649 global patients, OS, CHEMO, CPS<1  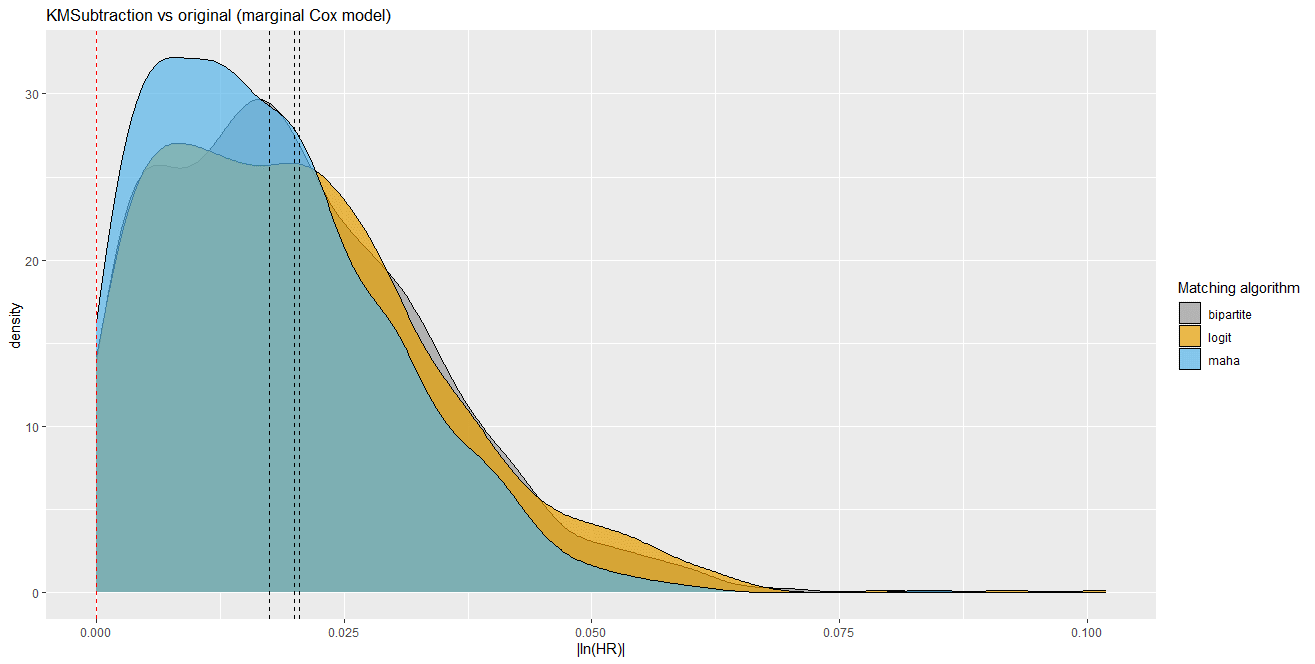 | | Convergence plot  CM-649 global patients, OS, CHEMO, CPS<1  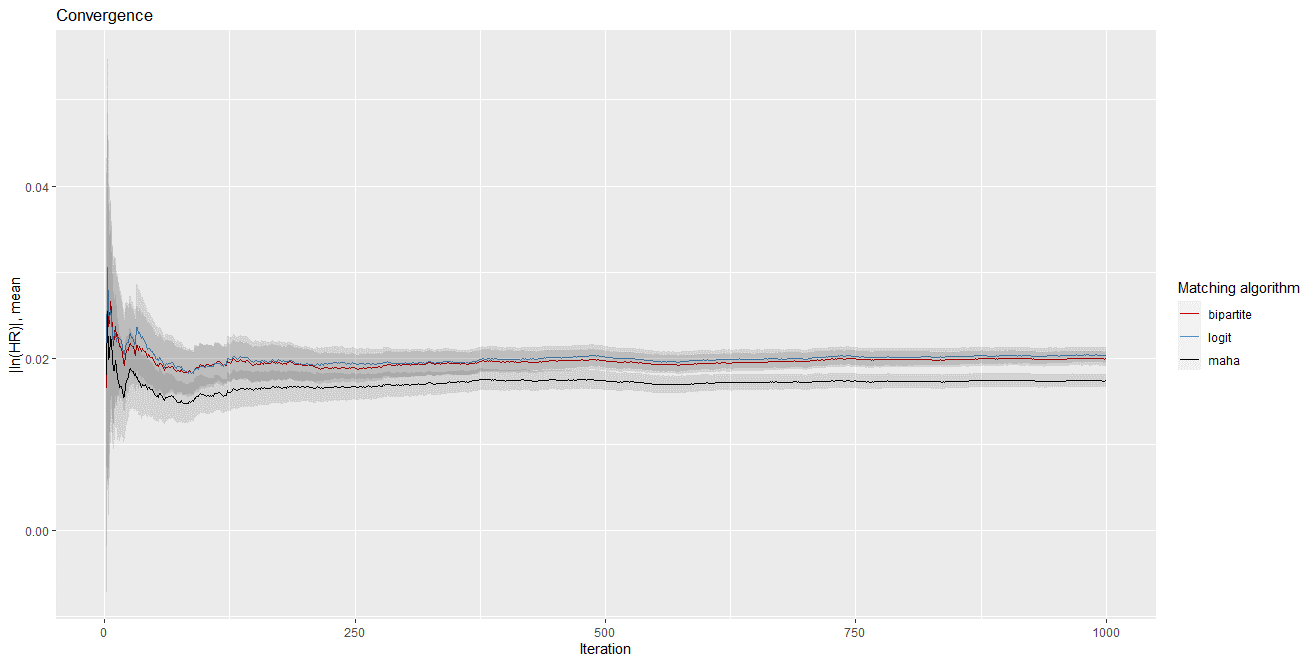 |
| Histogram of ln(HR)  CM-649 global patients, PFS, ICI+CHEMO, CPS<5  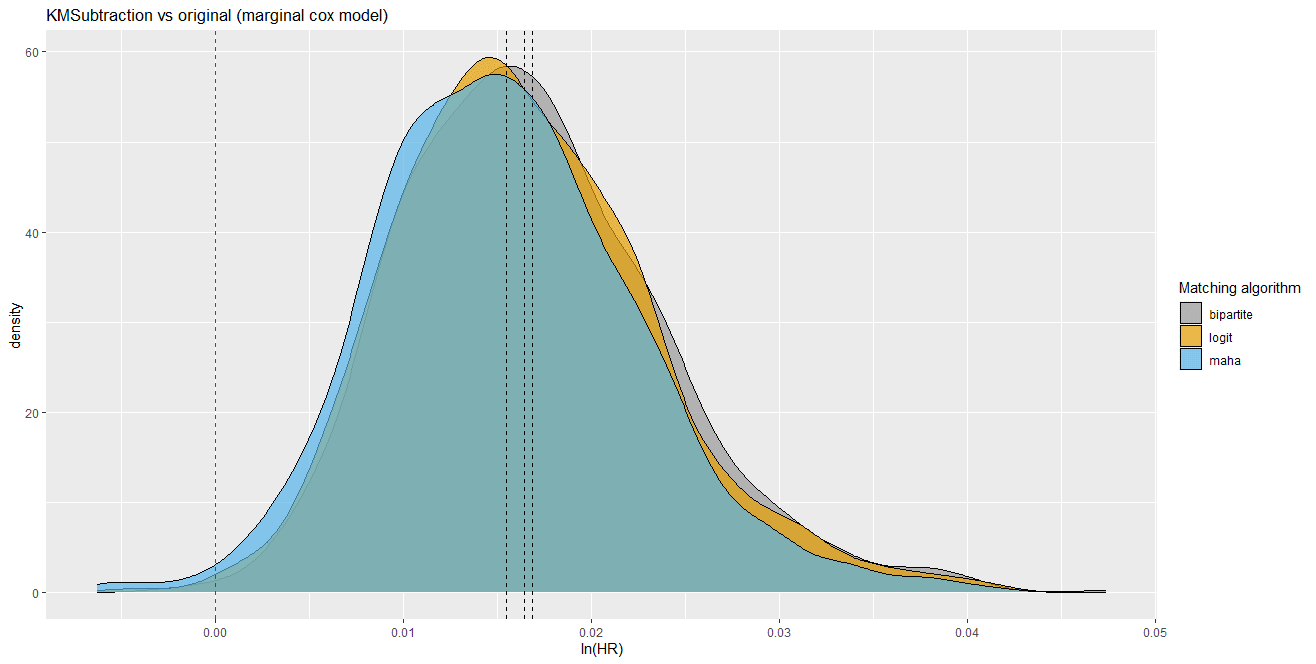 | Histogram of \|ln(HR)\|  CM-649 global patients, PFS, ICI+CHEMO, CPS<5  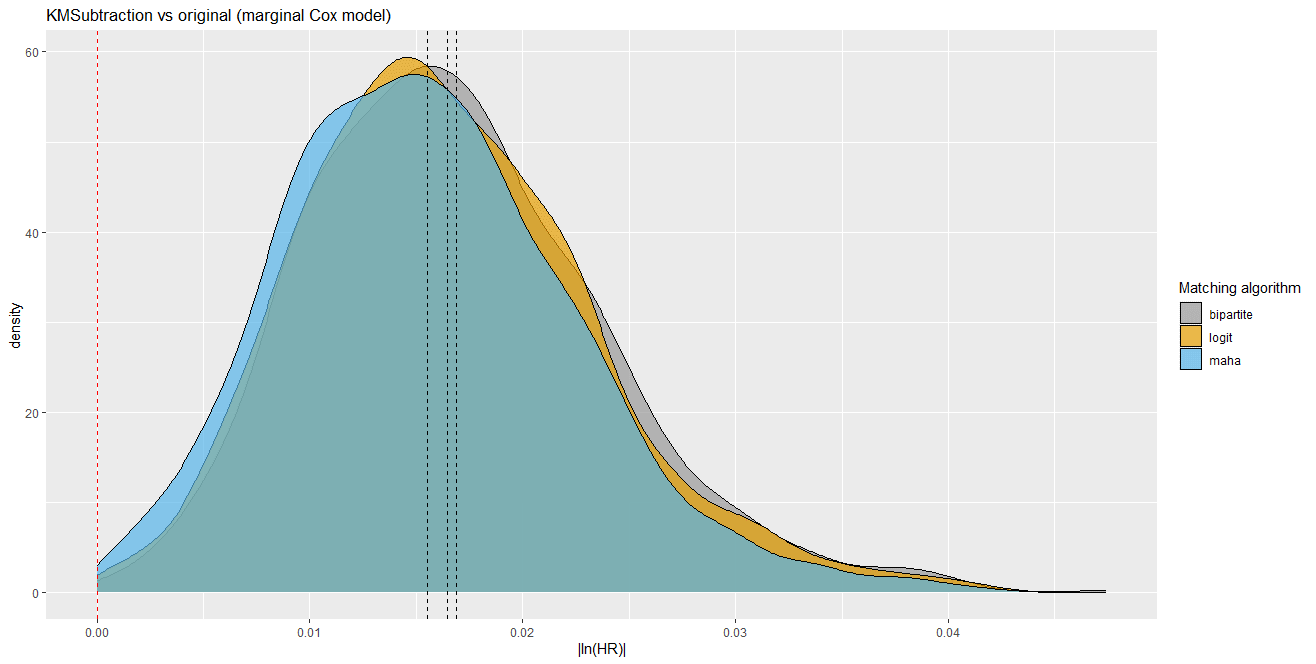 | | Convergence plot  CM-649 global patients, PFS, ICI+CHEMO, CPS<5  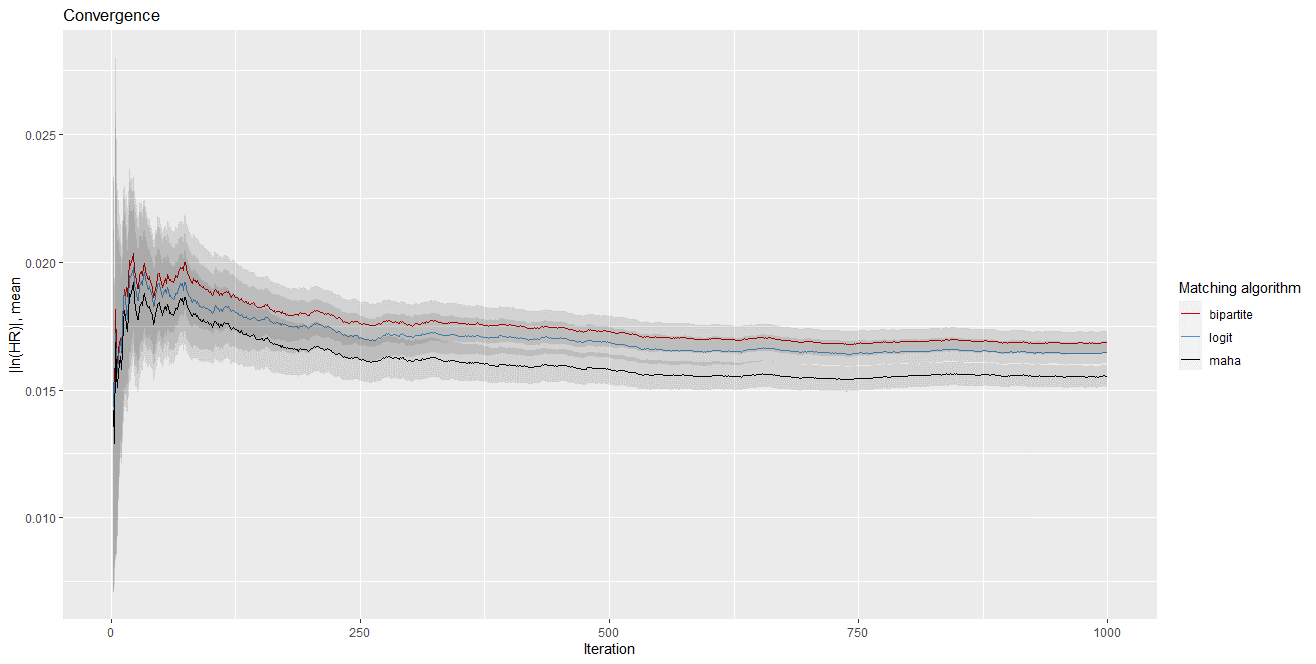 |
| Histogram of ln(HR)  CM-649 global patients, PFS, CHEMO, CPS<5  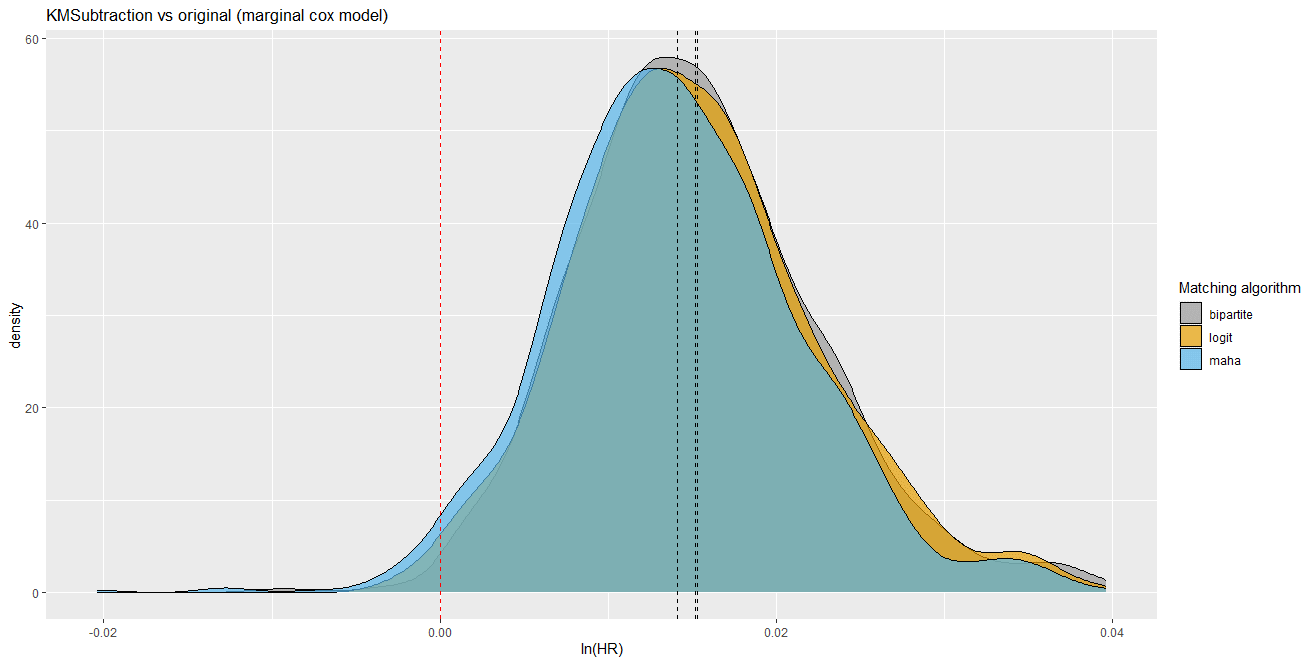 | Histogram of \|ln(HR)\|  CM-649 global patients, PFS, CHEMO, CPS<5  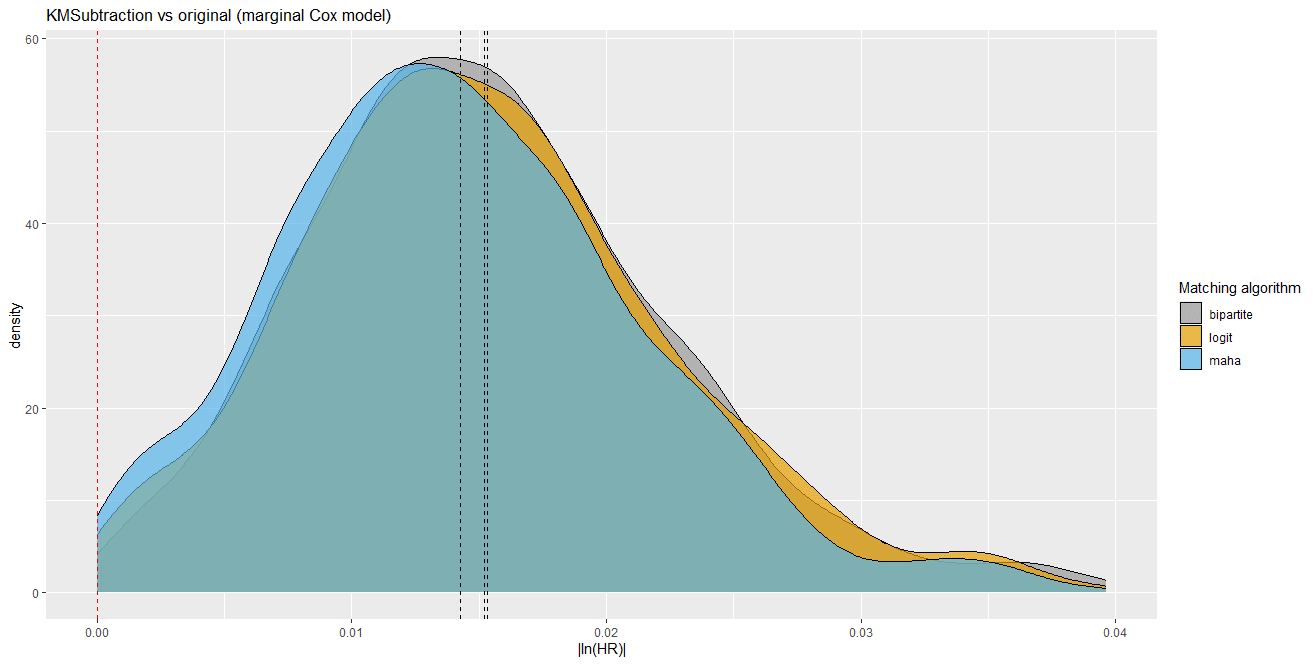 | | Convergence plot  CM-649 global patients, PFS, CHEMO, CPS<5  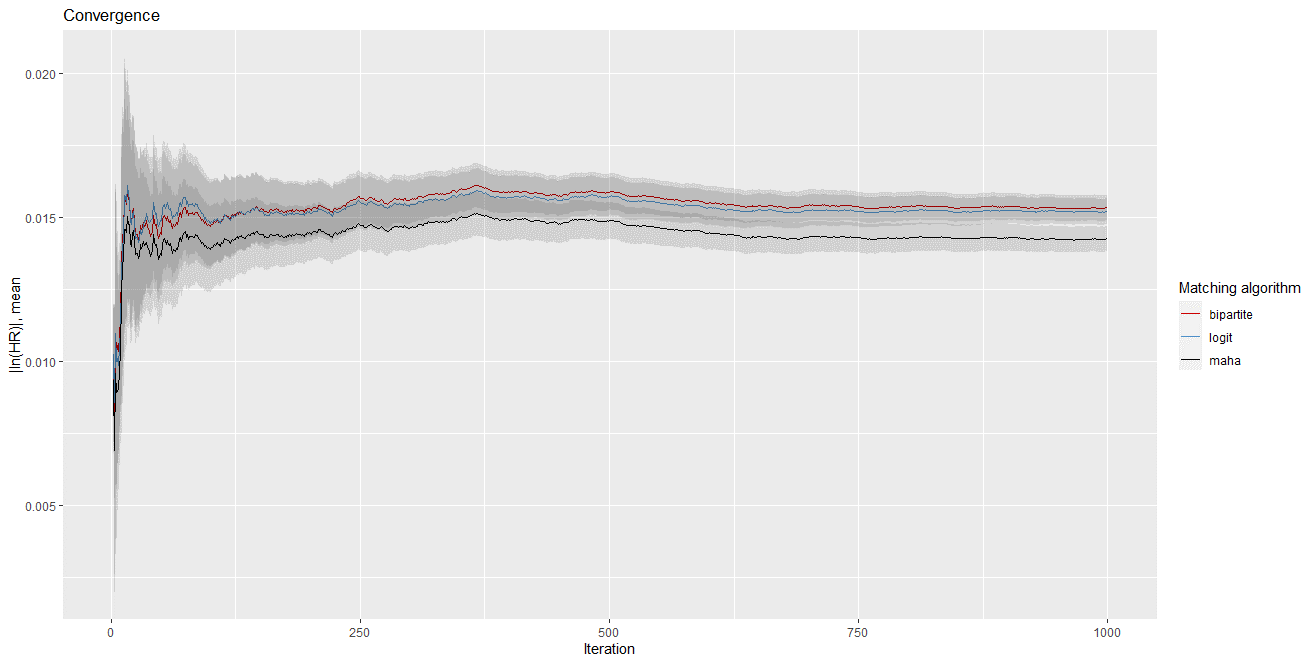 |
| Histogram of ln(HR)  CM-649 global patients, PFS, ICI+CHEMO, CPS<1  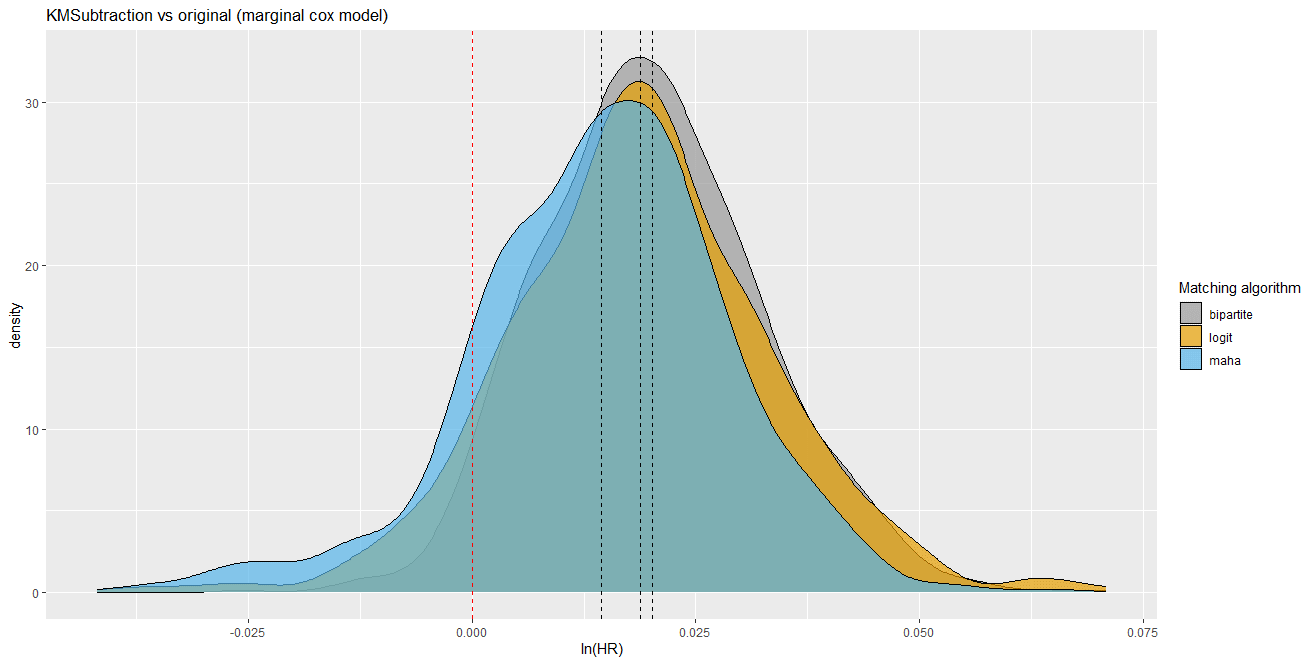 | Histogram of \|ln(HR)\|  CM-649 global patients, PFS, ICI+CHEMO, CPS<1  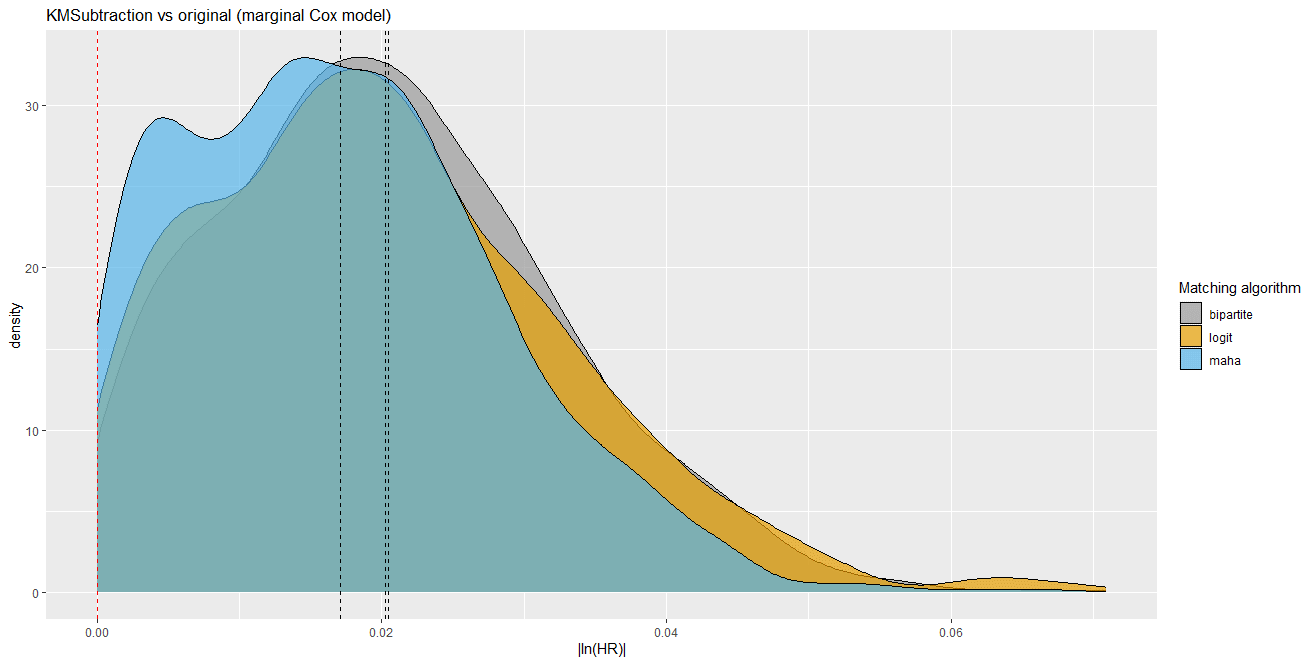 | | Convergence plot  CM-649 global patients, PFS, ICI+CHEMO, CPS<1  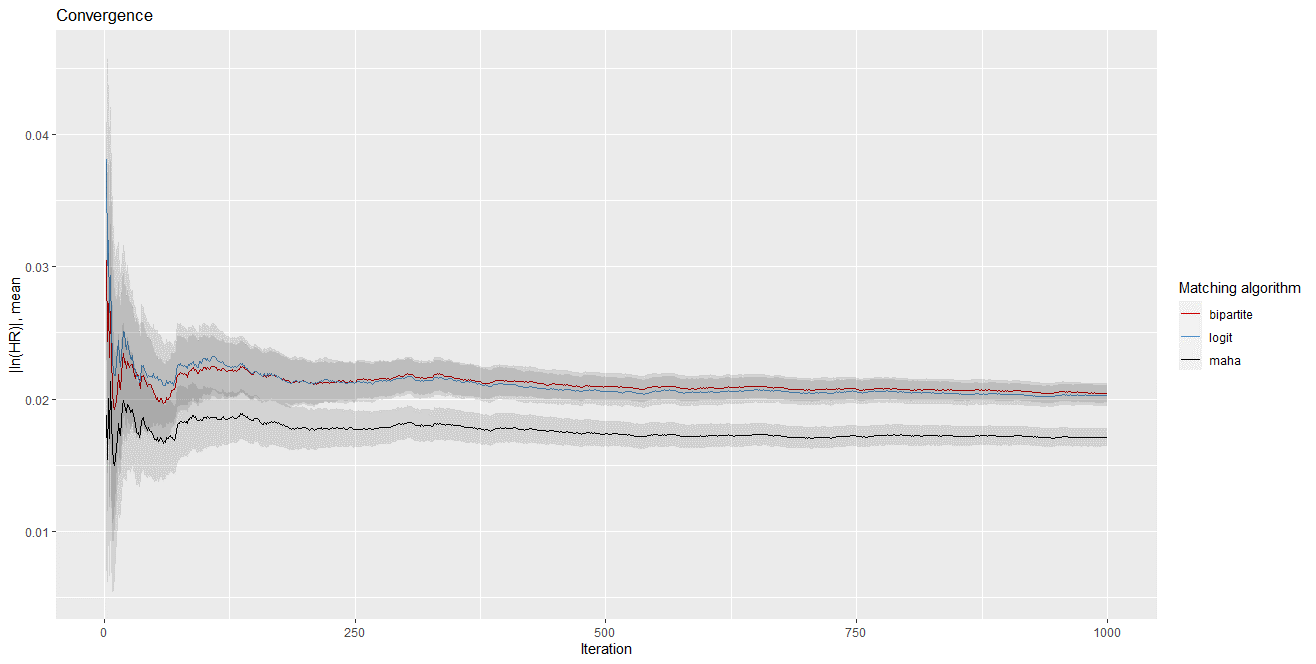 |
| Histogram of ln(HR)  CM-649 global patients, PFS, CHEMO, CPS<1  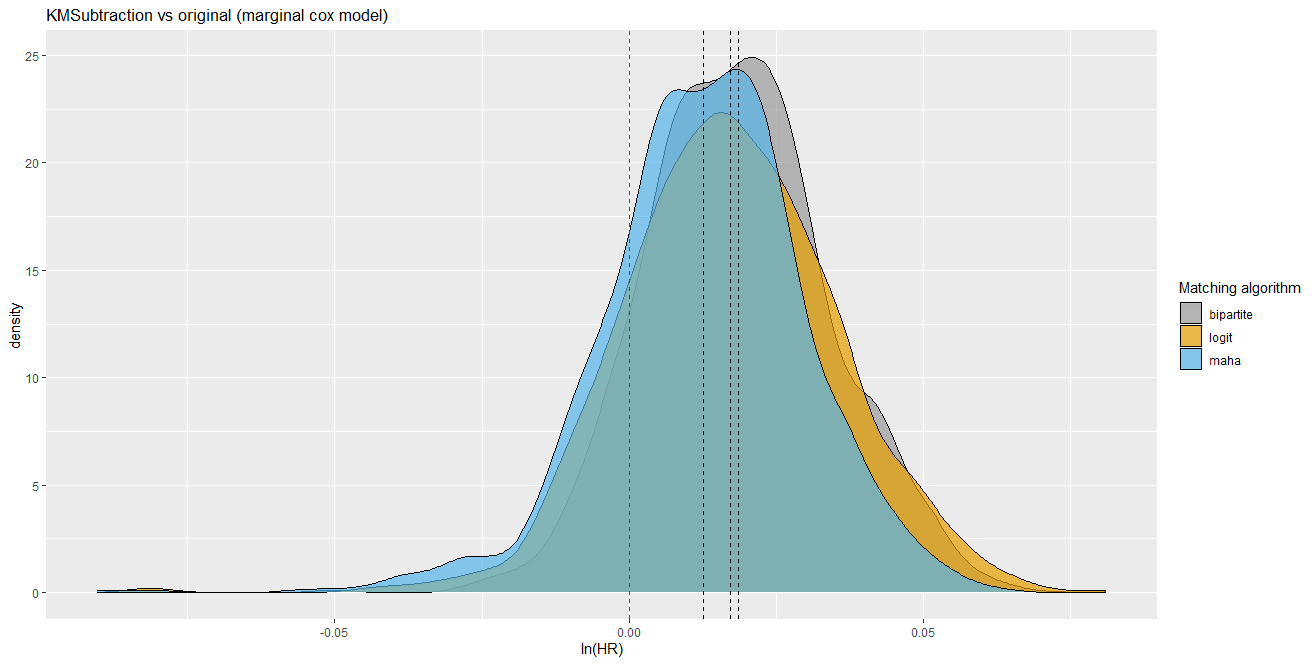 | Histogram of \|ln(HR)\|  CM-649 global patients, PFS, CHEMO, CPS<1  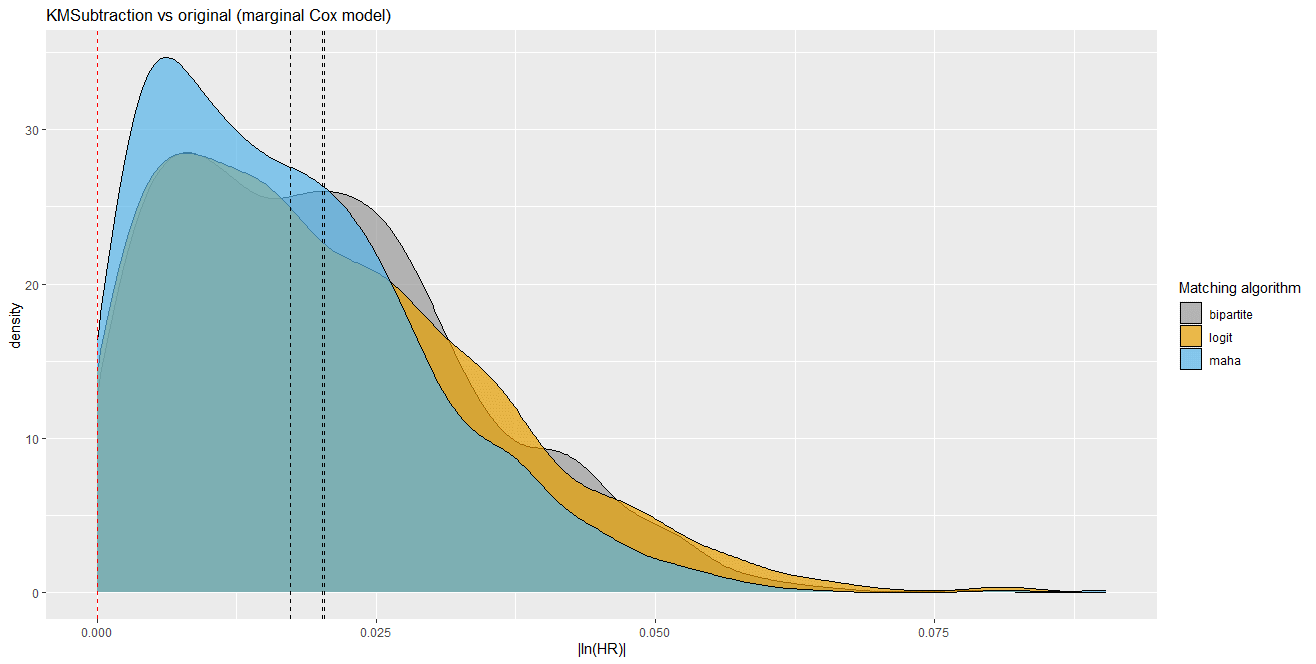 | | Convergence plot  CM-649 global patients, PFS, CHEMO, CPS<1  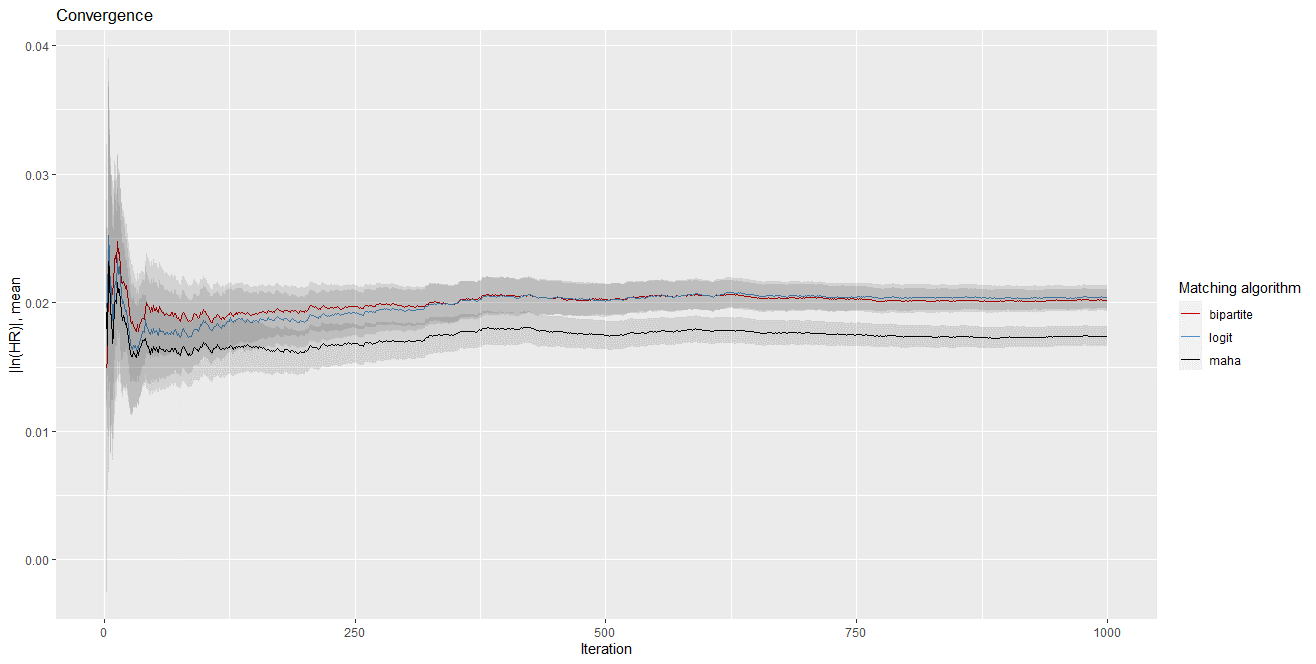 |
| Histogram of ln(HR)  CM-649 Asian patients, OS, ICI+CHEMO, CPS<5  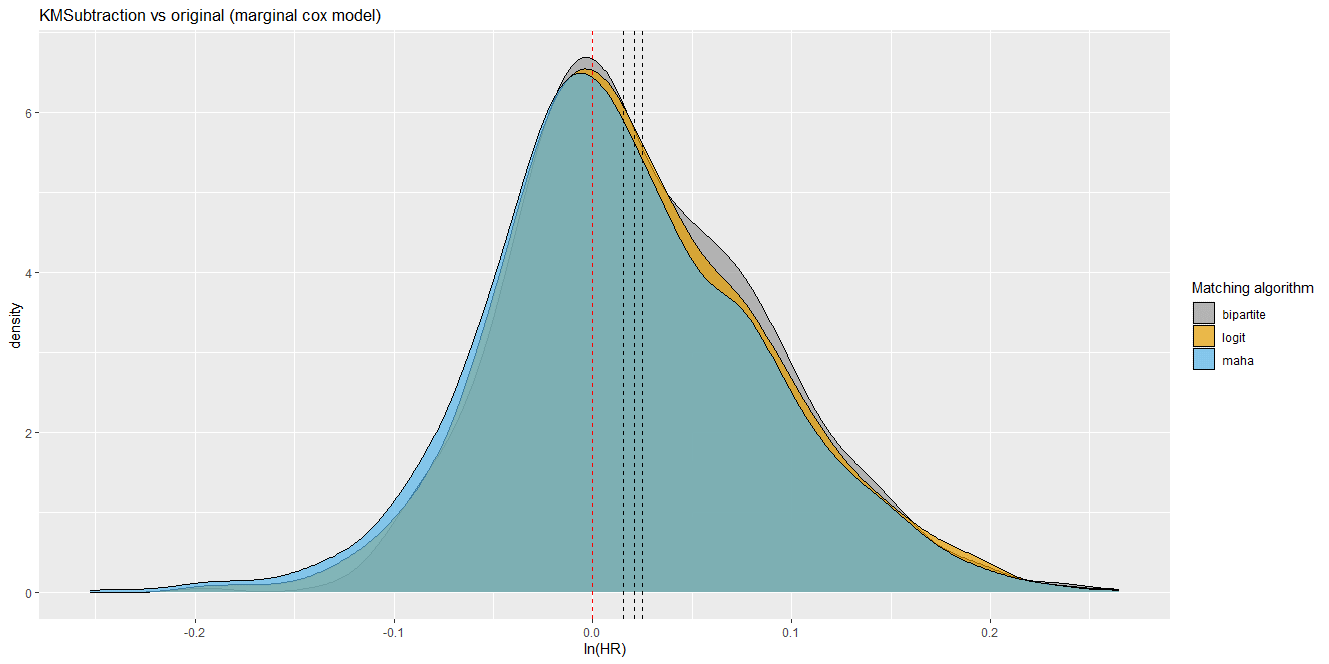 | Histogram of \|ln(HR)\|  CM-649 Asian patients, OS, ICI+CHEMO, CPS<5  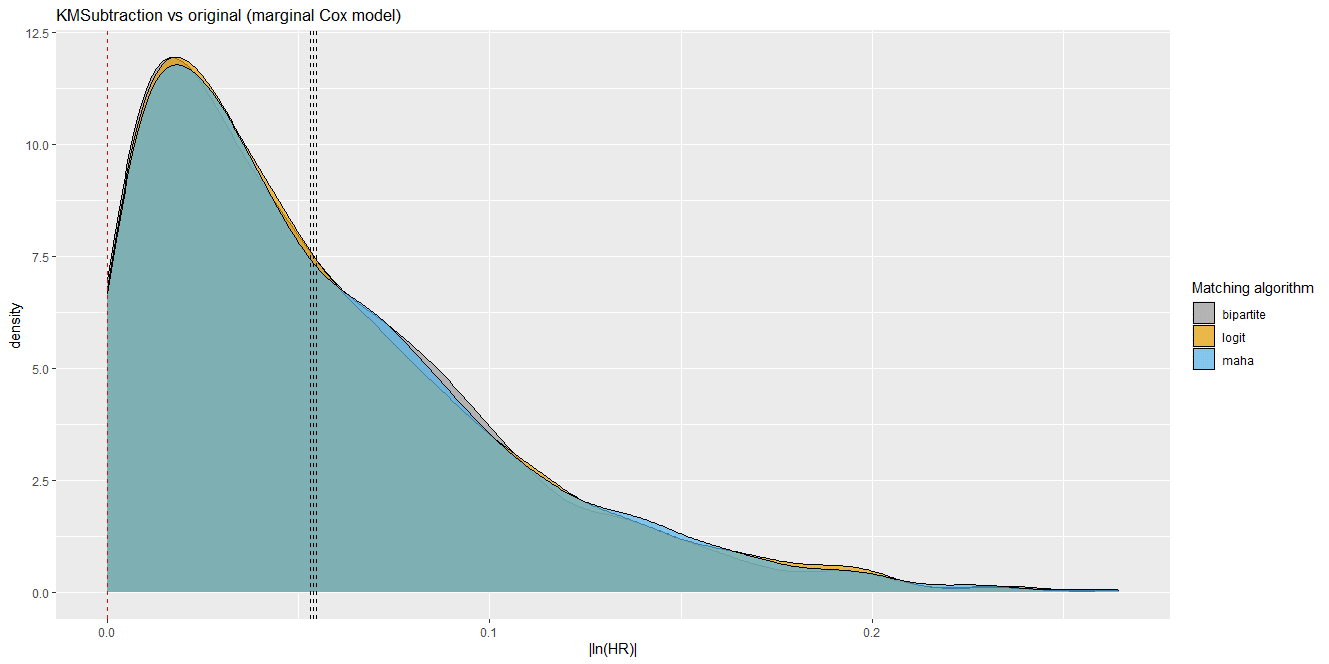 | | Convergence plot  CM-649 Asian patients, OS, ICI+CHEMO, CPS<5  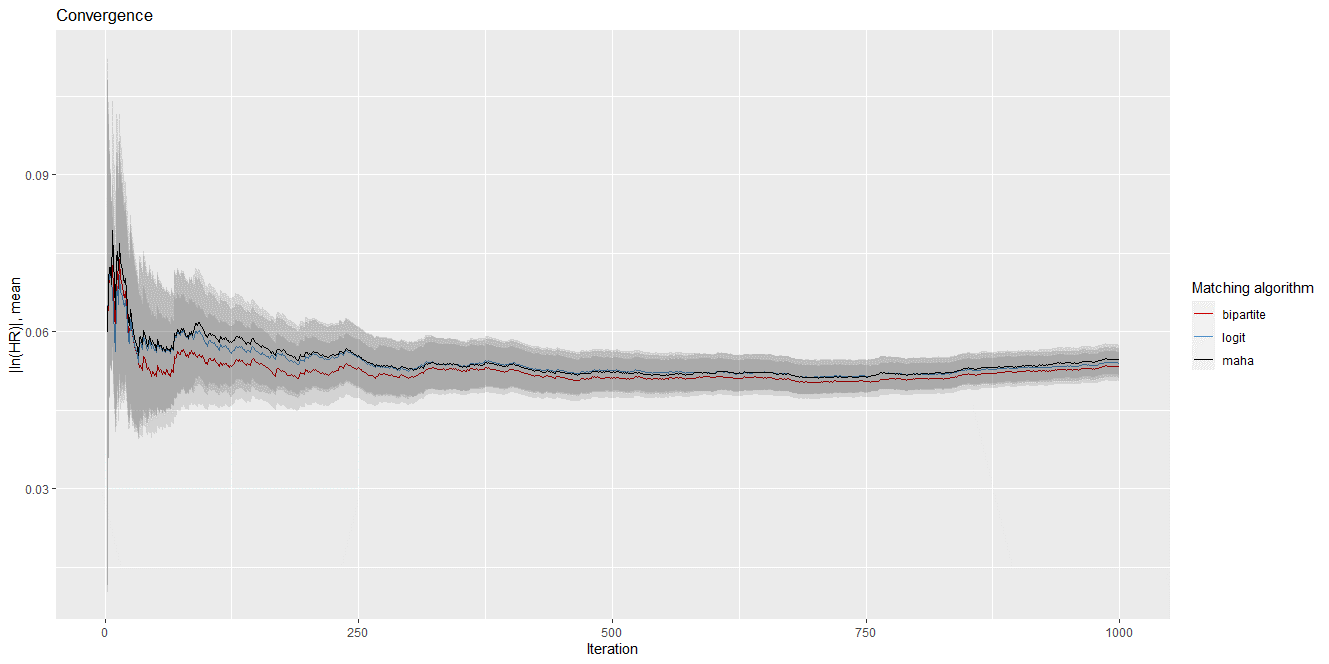 |
| Histogram of ln(HR)  CM-649 Asian patients, OS, CHEMO, CPS<5  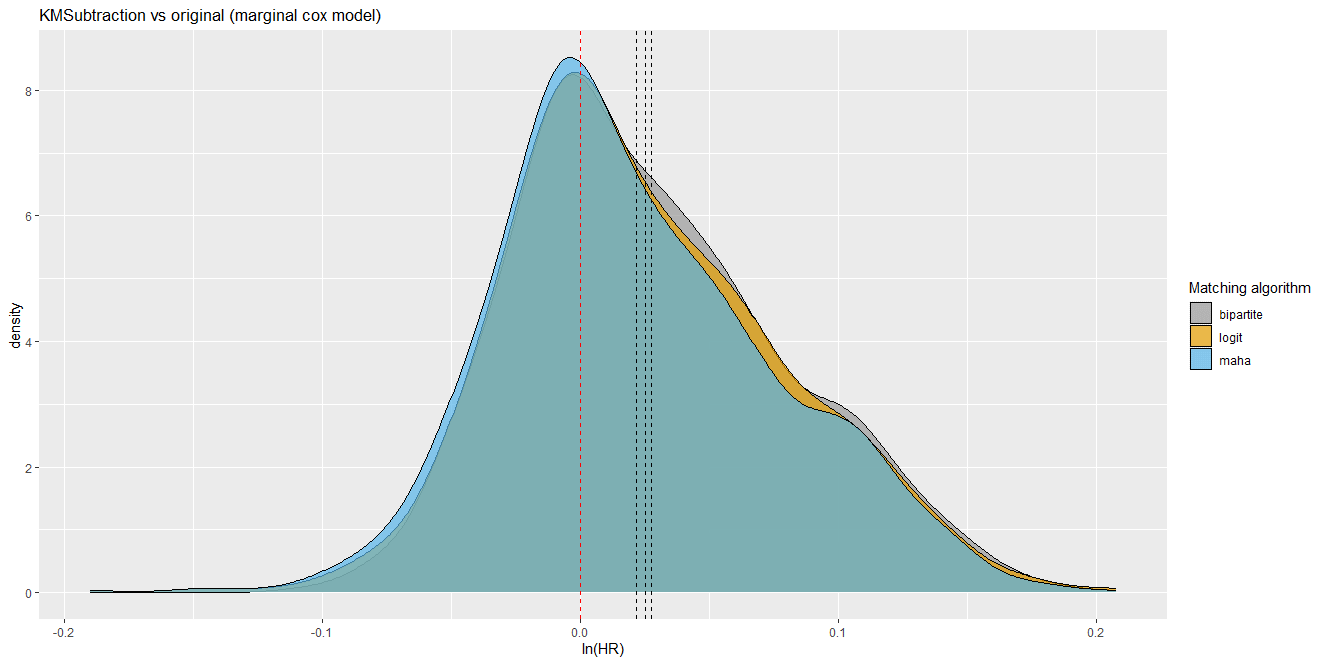 | Histogram of \|ln(HR)\|  CM-649 Asian patients, OS, CHEMO, CPS<5  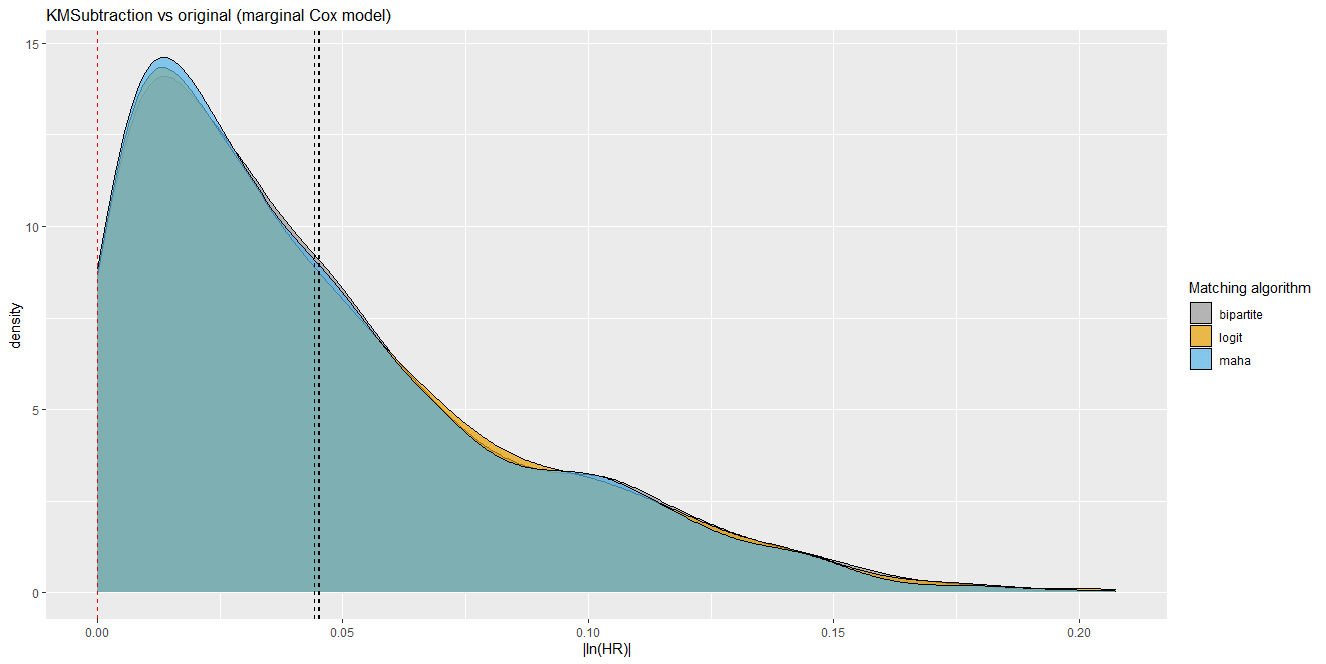 | | Convergence plot  CM-649 Asian patients, OS, CHEMO, CPS<5  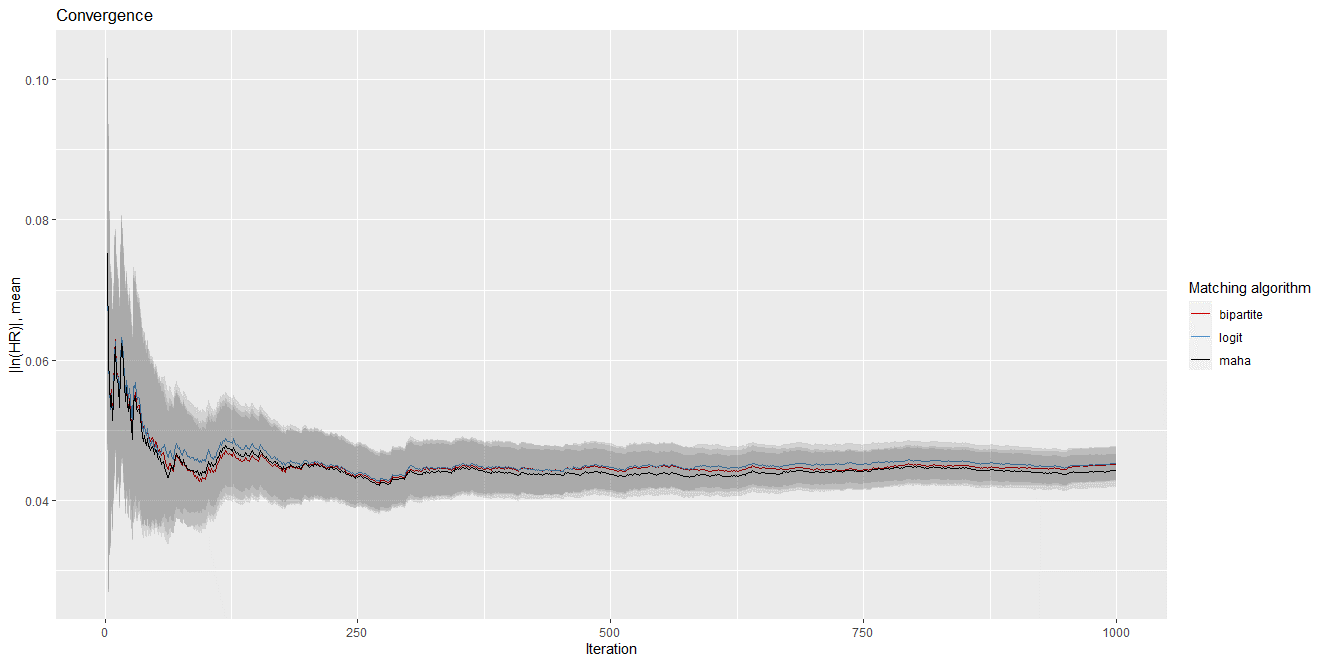 |
| Histogram of ln(HR)  CM-649 Asian patients, OS, ICI+CHEMO, CPS<1  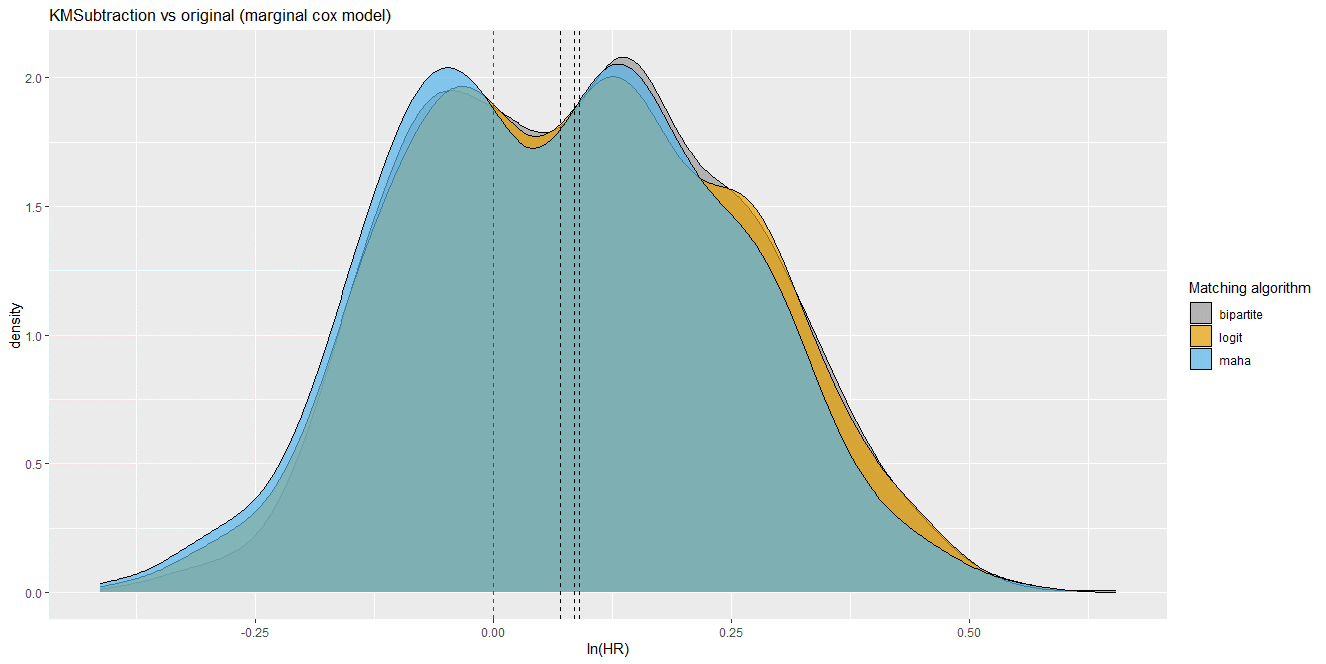 | Histogram of \|ln(HR)\|  CM-649 Asian patients, OS, ICI+CHEMO, CPS<1  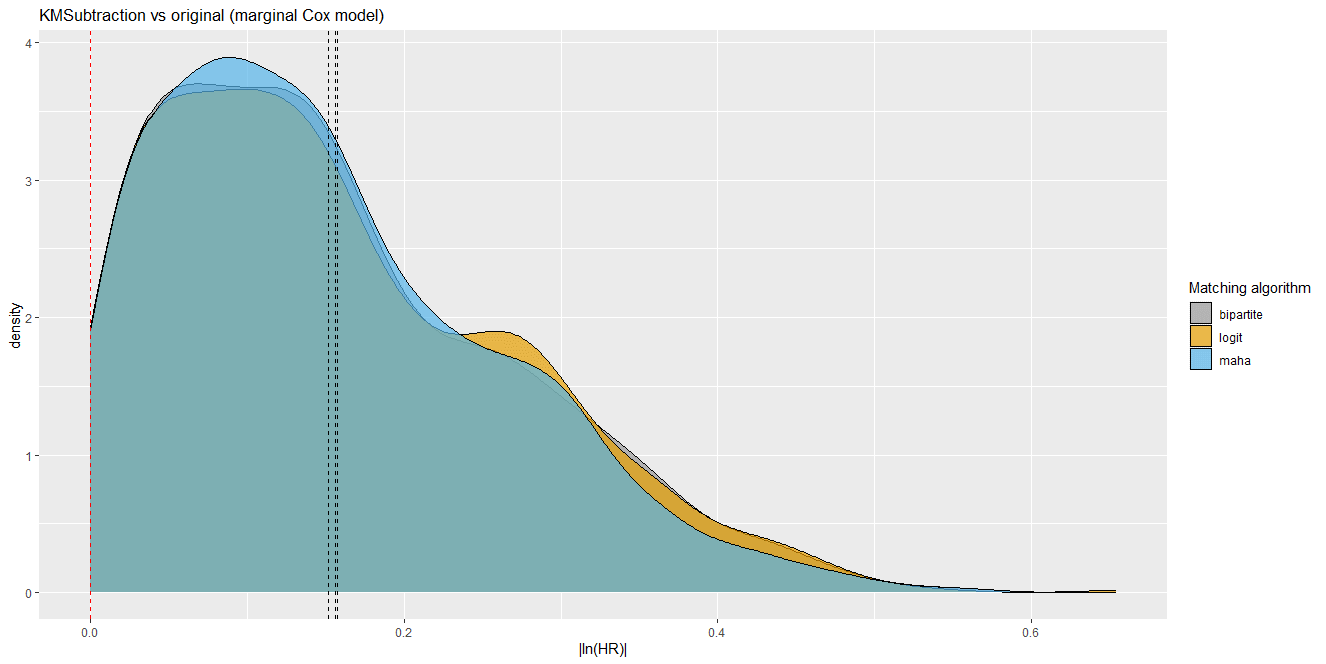 | | Convergence plot  CM-649 Asian patients, OS, ICI+CHEMO, CPS<1  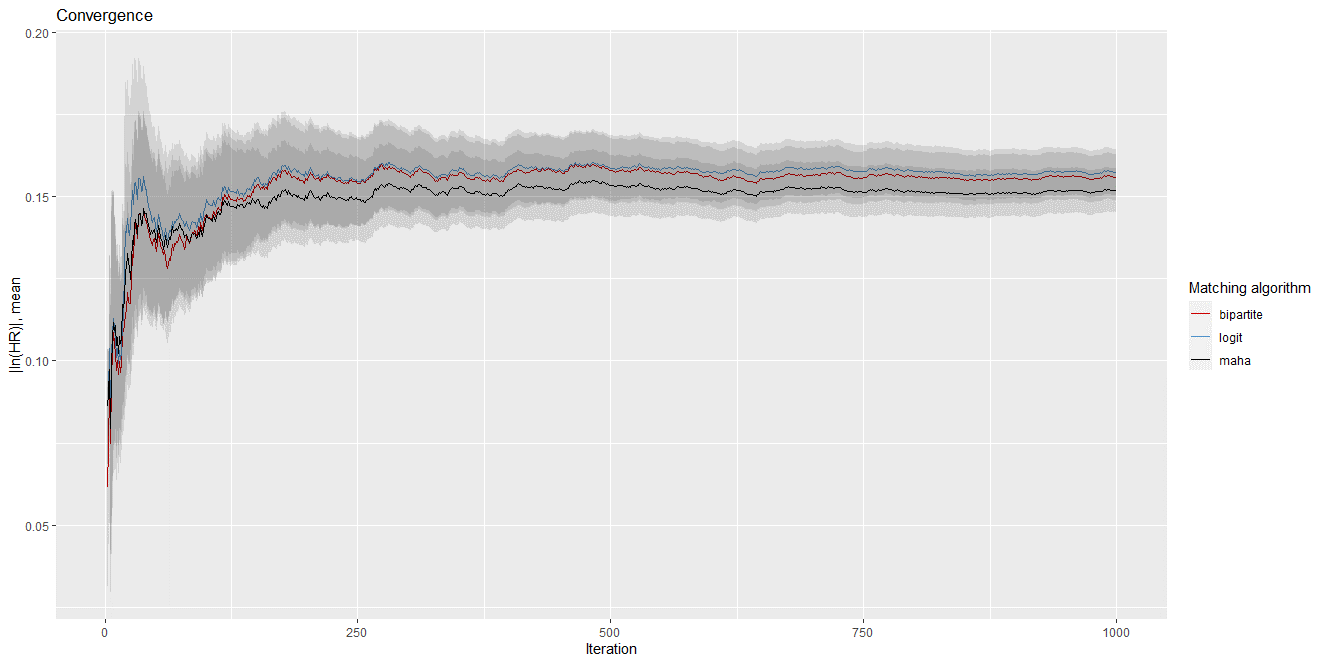 |
| Histogram of ln(HR)  CM-649 Asian patients, OS, CHEMO, CPS<1  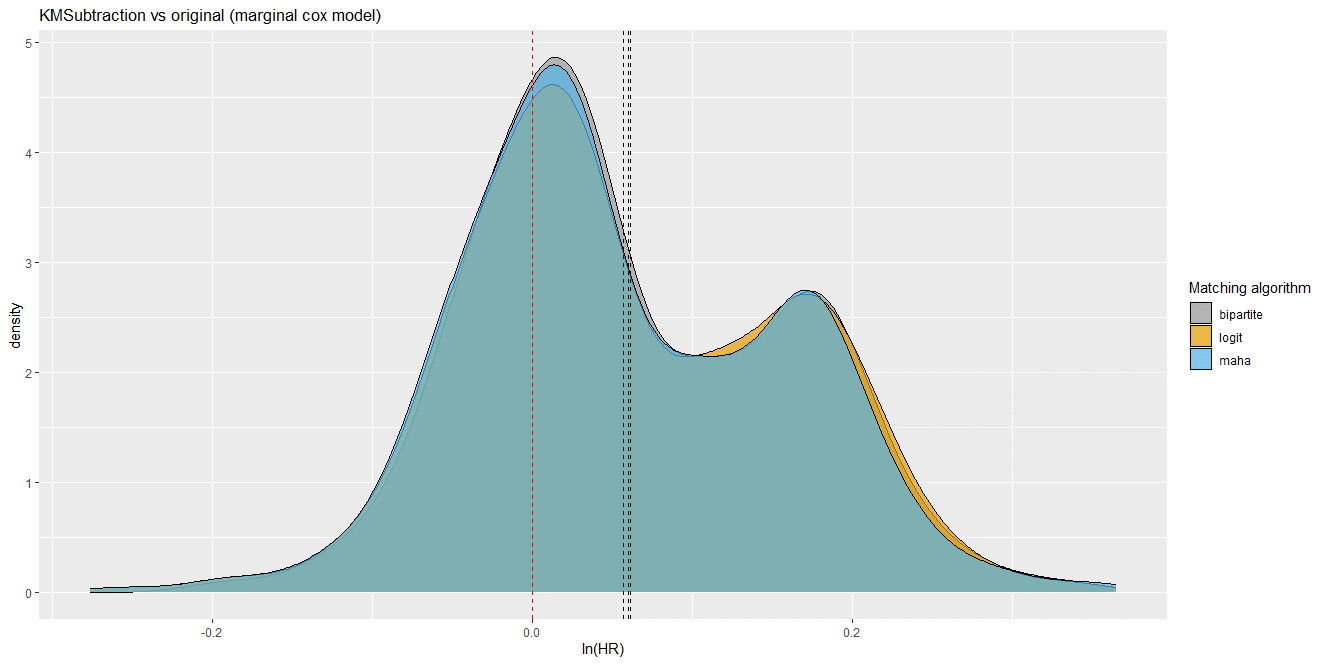 | Histogram of \|ln(HR)\|  CM-649 Asian patients, OS, CHEMO, CPS<1  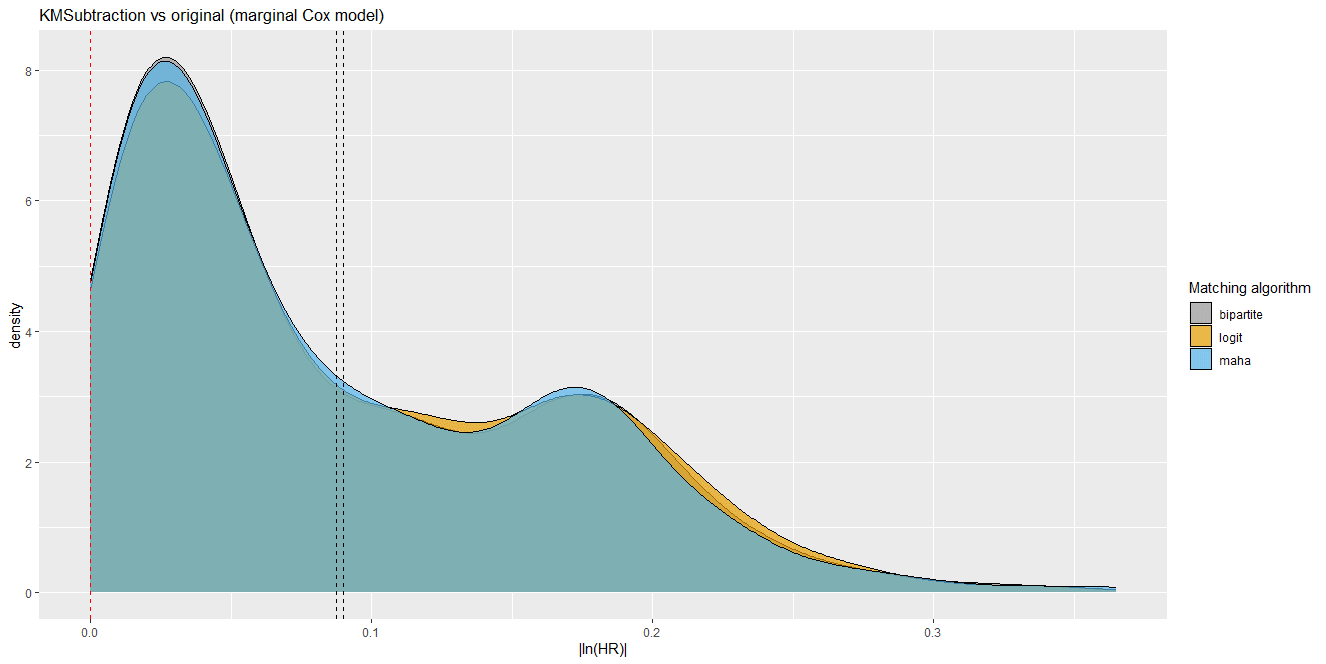 | | Convergence plot  CM-649 Asian patients, OS, CHEMO, CPS<1  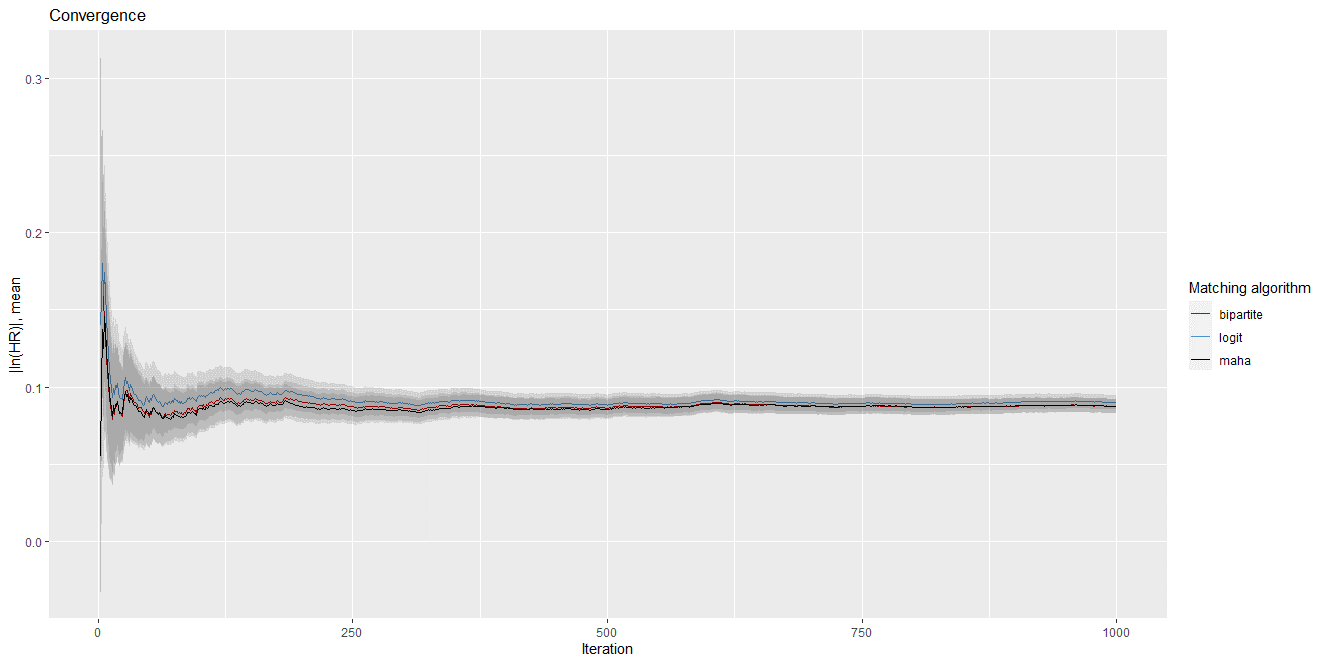 |
| Histogram of ln(HR)  CM-649 Asian patients, PFS, ICI+CHEMO, CPS<5  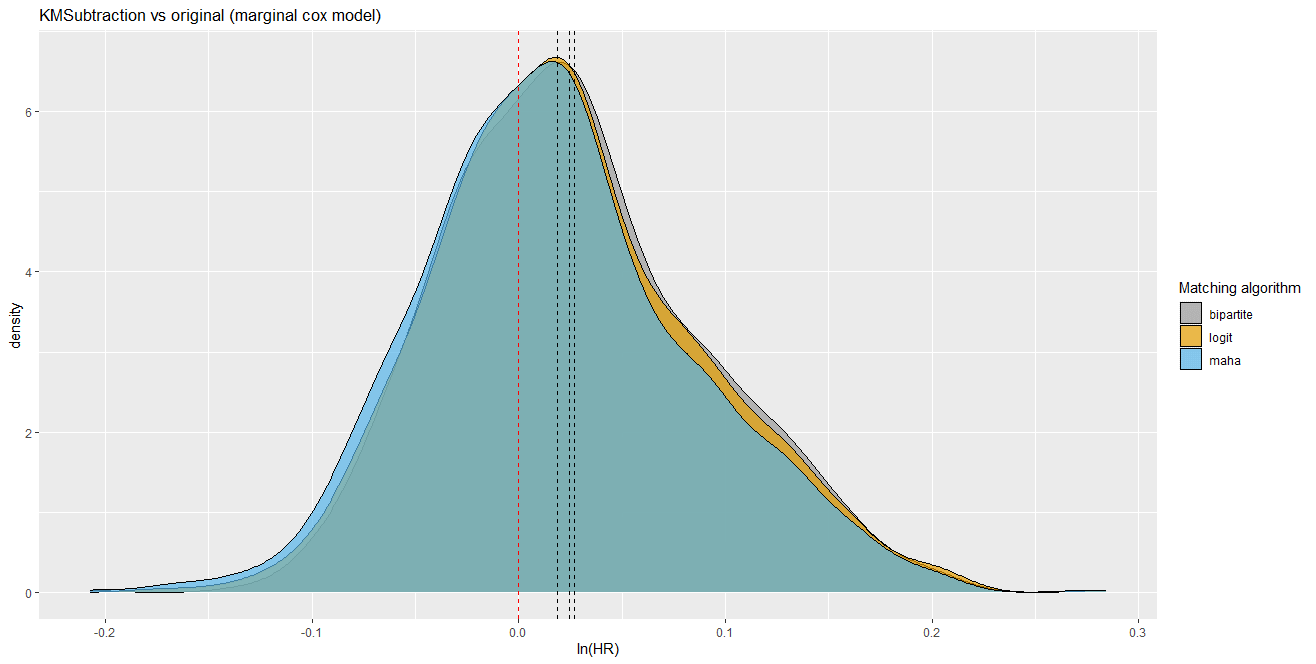 | | Histogram of \|ln(HR)\|  CM-649 Asian patients, PFS, ICI+CHEMO, CPS<5  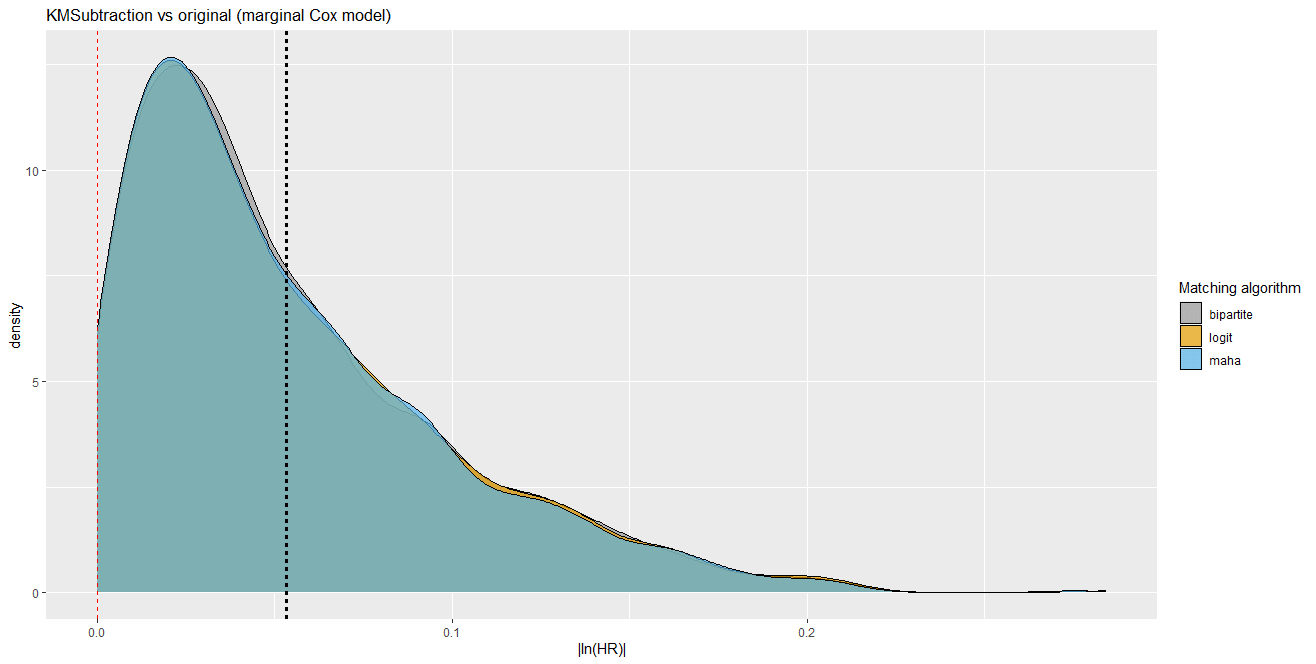 | Convergence plot  CM-649 Asian patients, PFS, ICI+CHEMO, CPS<5  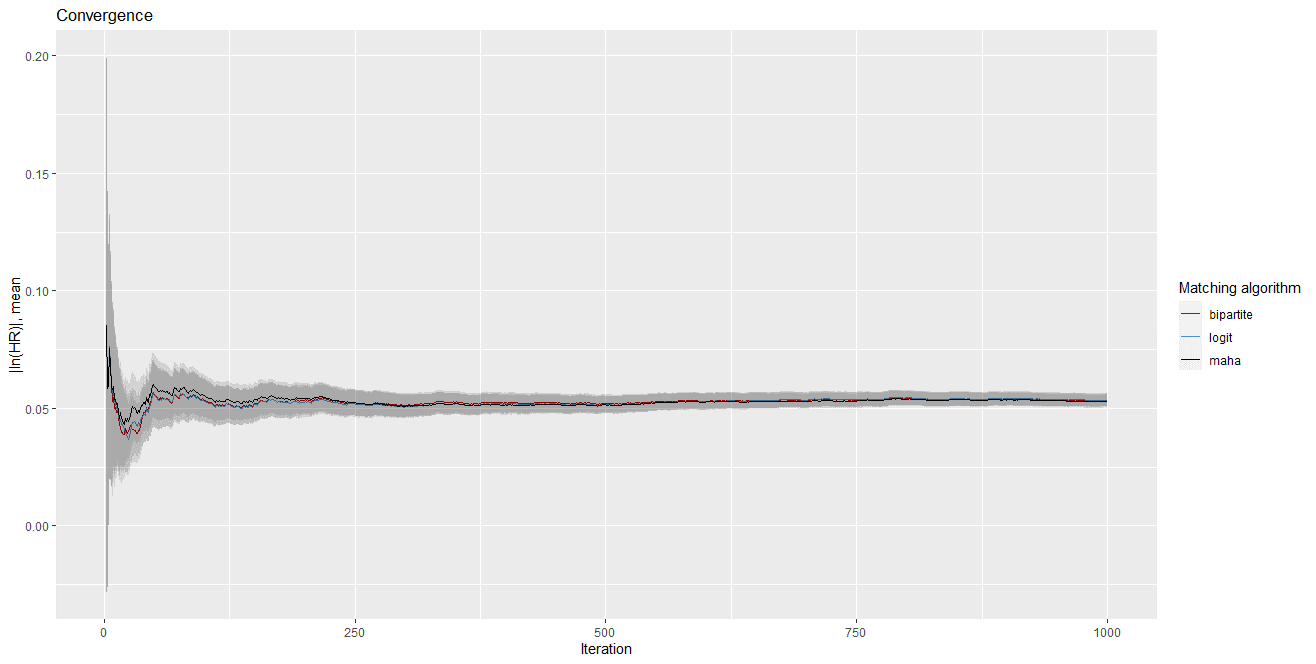 |
| Histogram of ln(HR)  CM-649 Asian patients, PFS, CHEMO, CPS<5  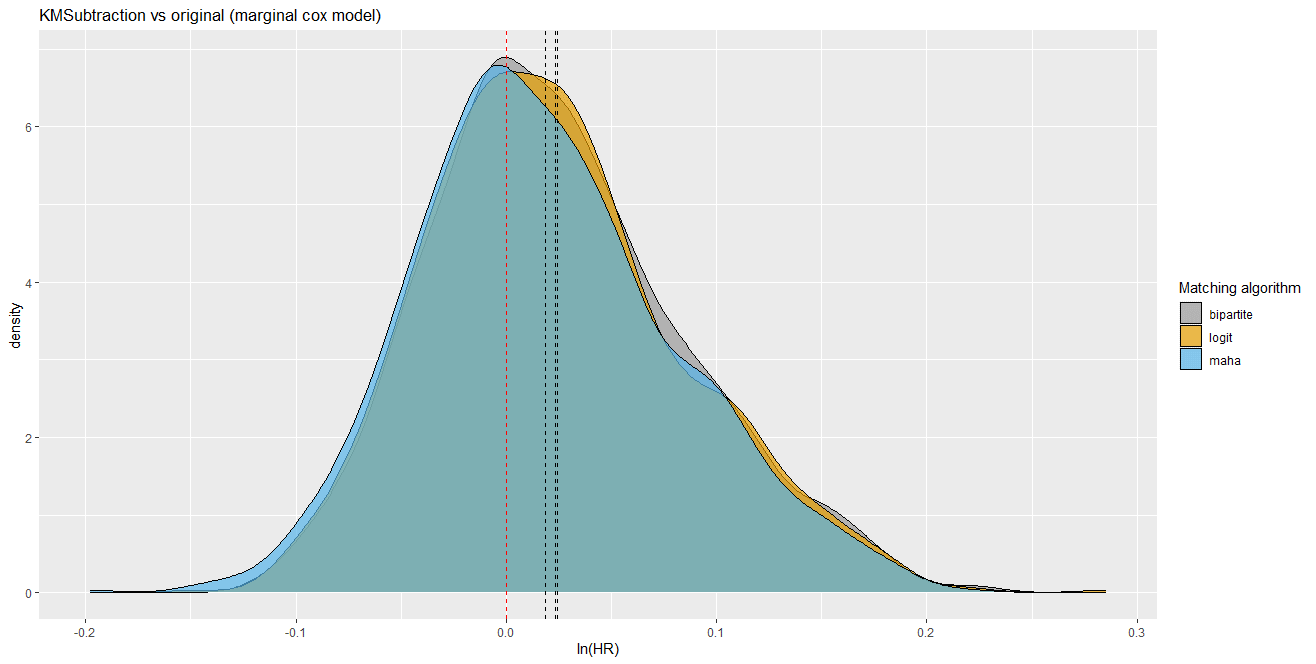 | | Histogram of \|ln(HR)\|  CM-649 Asian patients, PFS, CHEMO, CPS<5  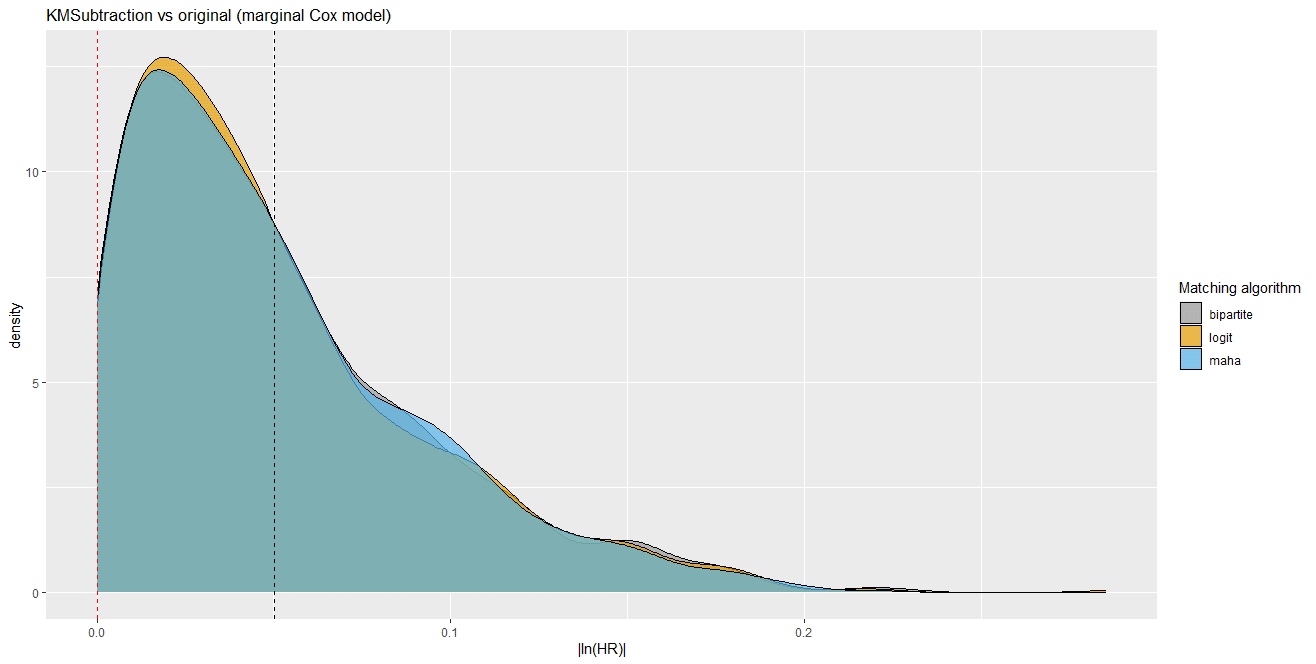 | Convergence plot  CM-649 Asian patients, PFS, CHEMO, CPS<5  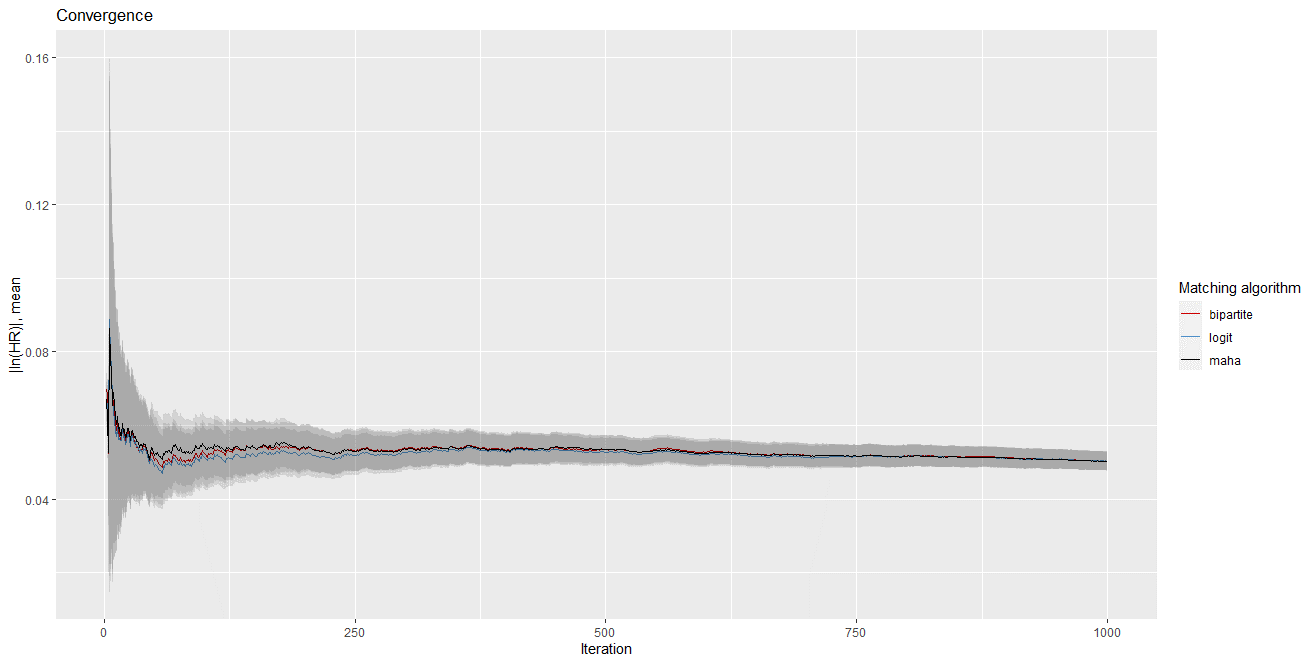 |
| Histogram of ln(HR)  CM-649 Asian patients, PFS, ICI+CHEMO, CPS<1 | | Histogram of \|ln(HR)\|  CM-649 Asian patients, PFS, ICI+CHEMO, CPS<1 | Convergence plot  CM-649 Asian patients, PFS, ICI+CHEMO, CPS<1 |
| Histogram of ln(HR)  CM-649 Asian patients, PFS, CHEMO, CPS<1  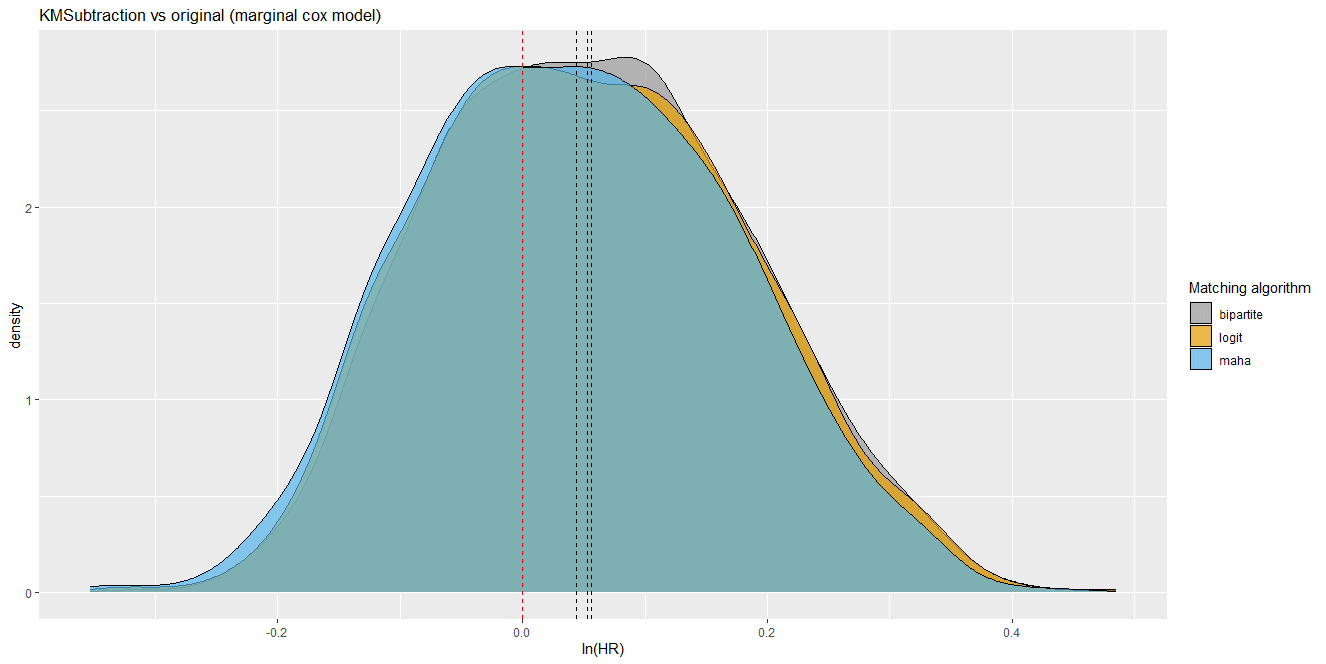 | | Histogram of \|ln(HR)\|  CM-649 Asian patients, PFS, CHEMO, CPS<1  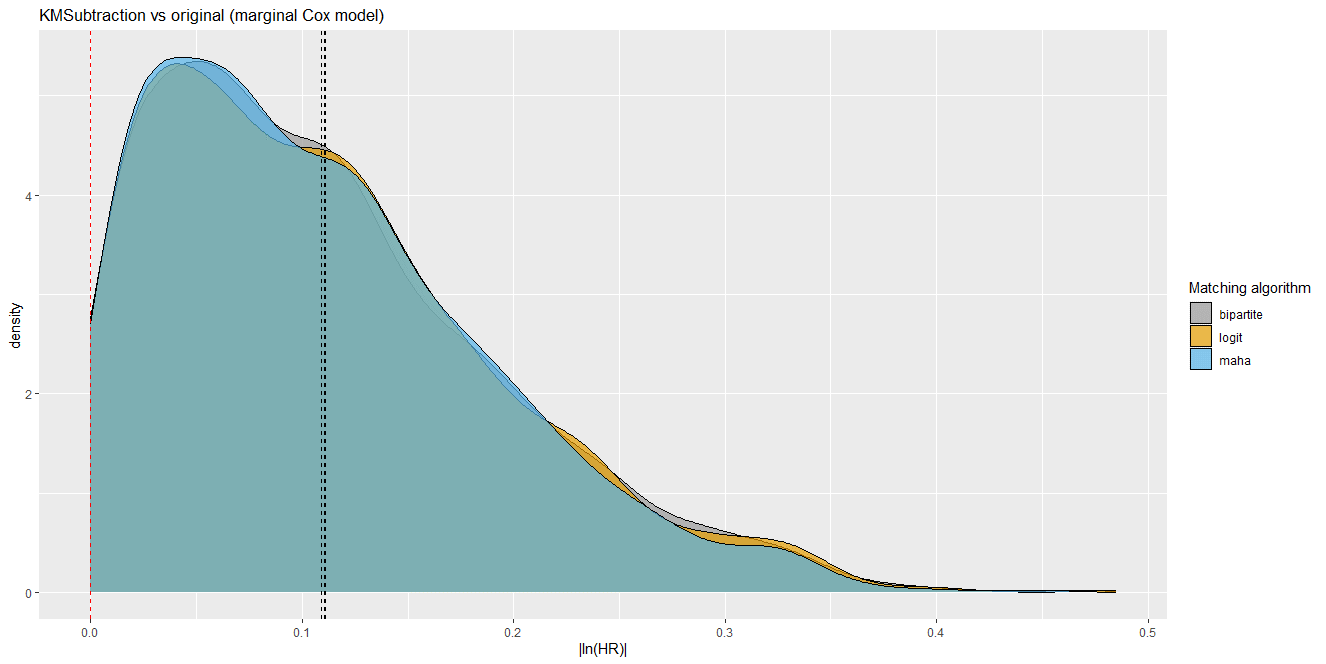 | Convergence plot  CM-649 Asian patients, PFS, CHEMO, CPS<1  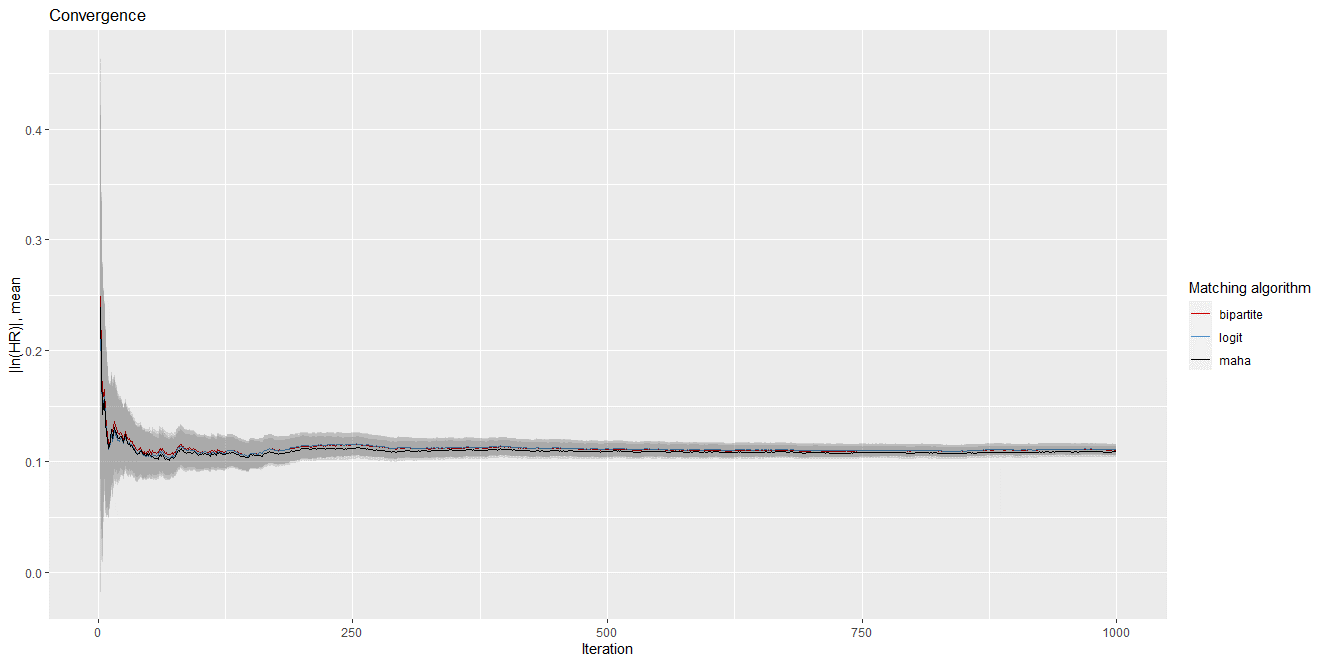 |
| Histogram of ln(HR)  ORIENT-16, OS, ICI+CHEMO, CPS<5  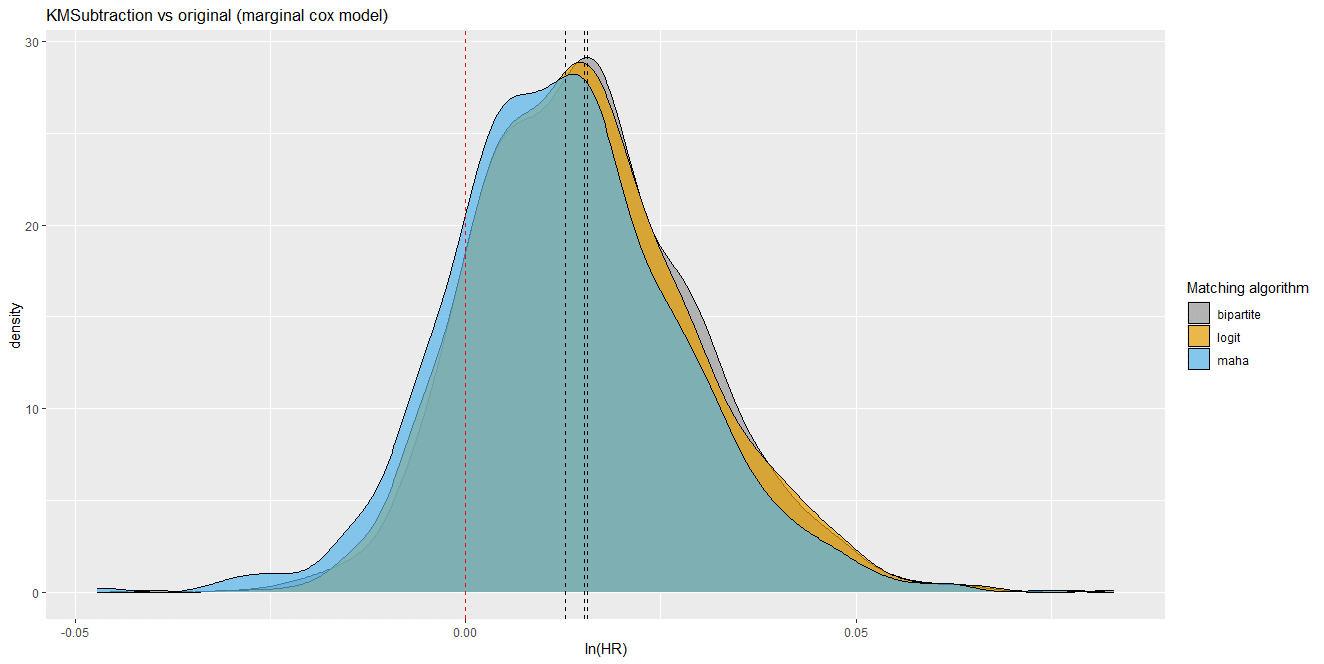 | | Histogram of \|ln(HR)\|  ORIENT-16, OS, ICI+CHEMO, CPS<5  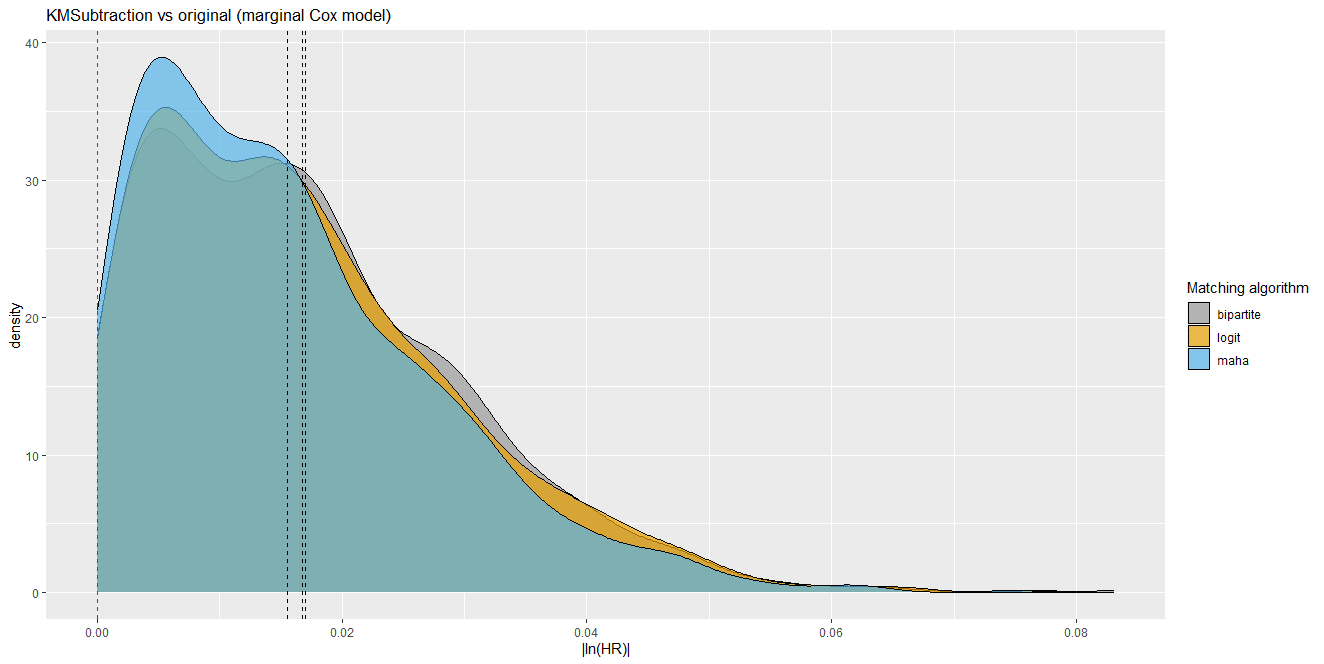 | Convergence plot  ORIENT-16, OS, ICI+CHEMO, CPS<5  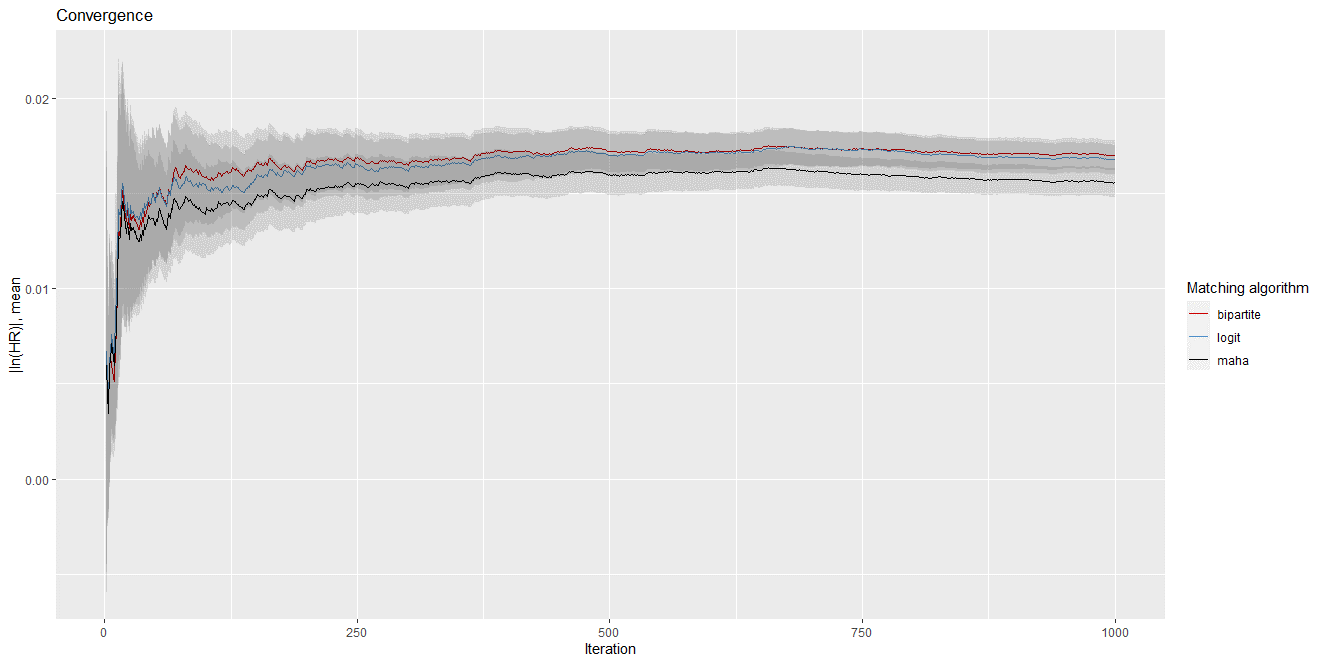 |
| Histogram of ln(HR)  ORIENT-16, OS, CHEMO, CPS<5  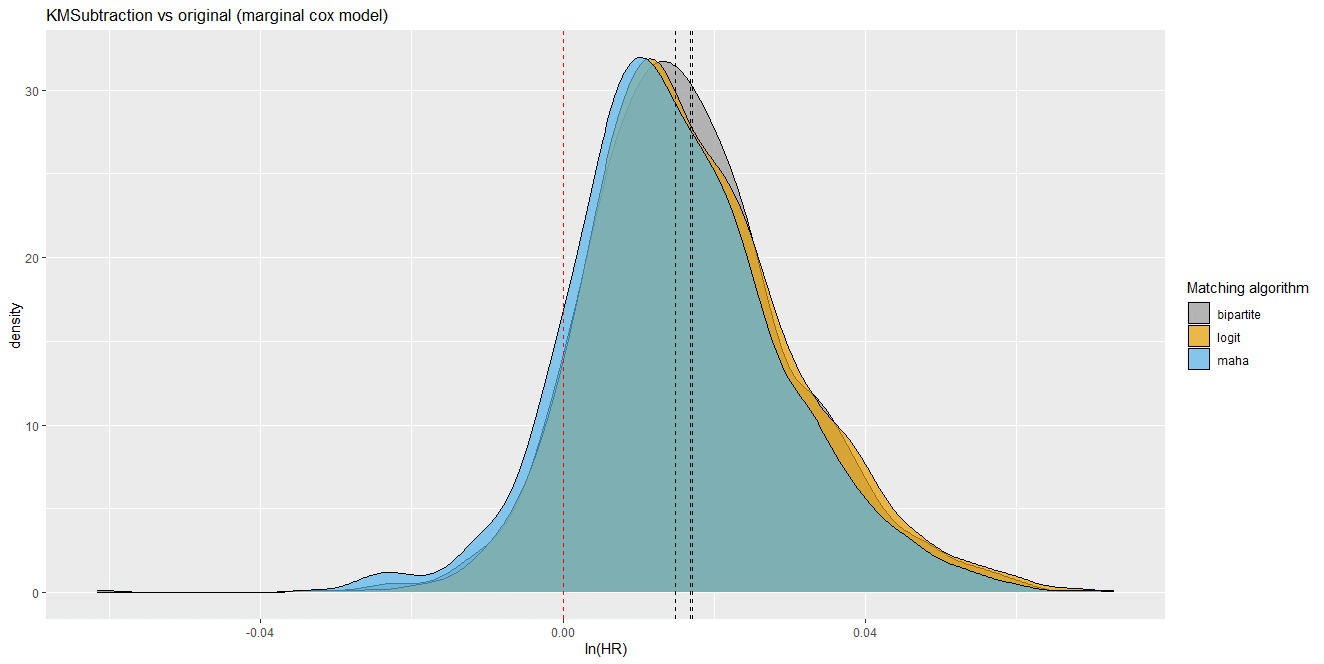 | | Histogram of \|ln(HR)\|  ORIENT-16, OS, CHEMO, CPS<5  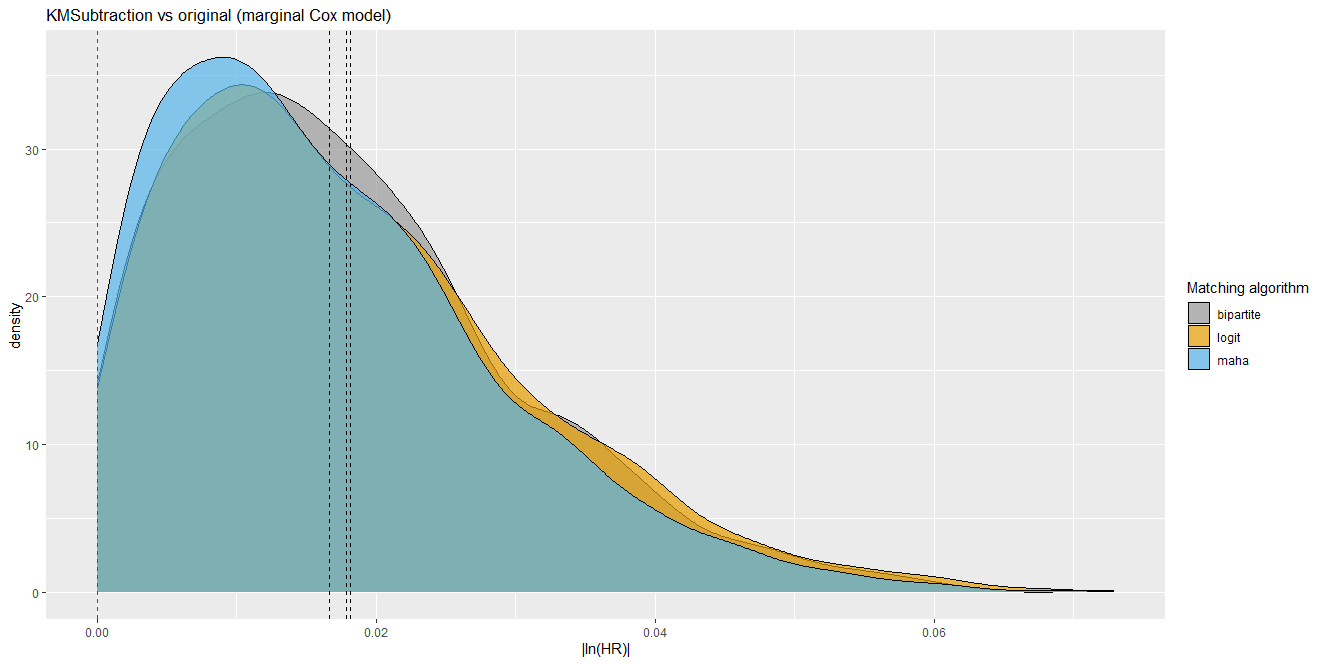 | Convergence plot  ORIENT-16, OS, CHEMO, CPS<5  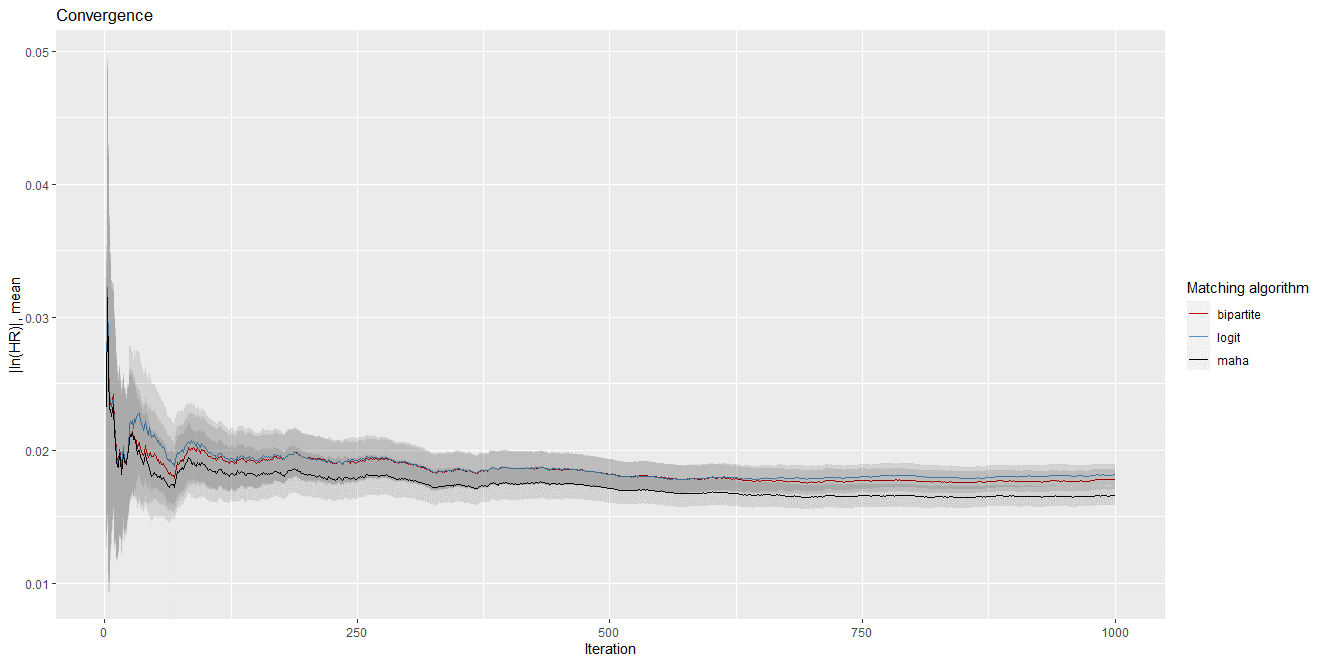 |
| Histogram of ln(HR)  ORIENT-16, PFS, ICI+CHEMO, CPS<5  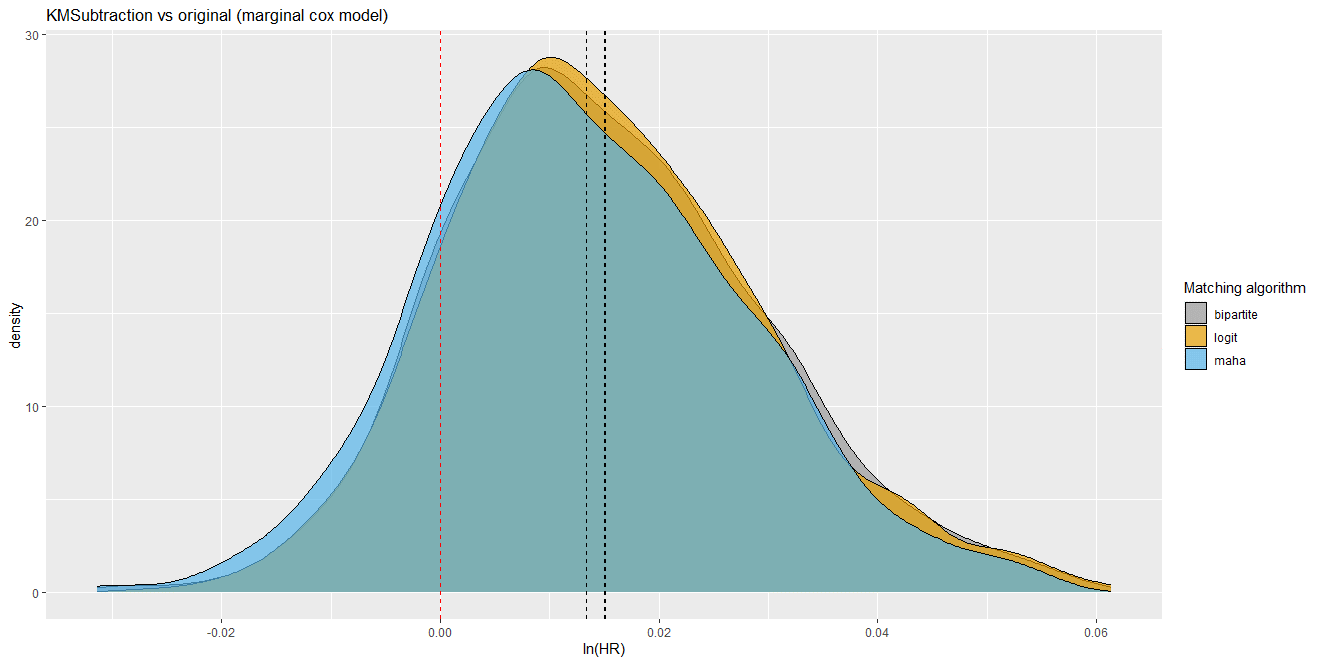 | | Histogram of \|ln(HR)\|  ORIENT-16, PFS, ICI+CHEMO, CPS<5  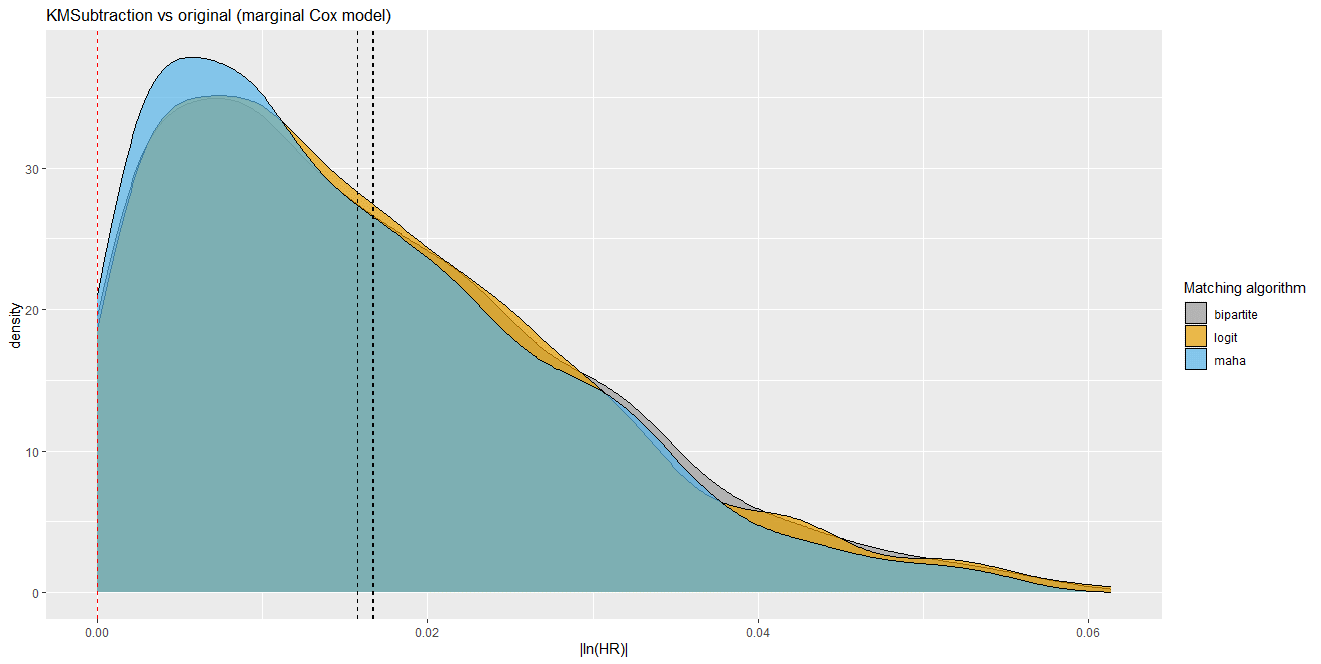 | Convergence plot  ORIENT-16, PFS, ICI+CHEMO, CPS<5  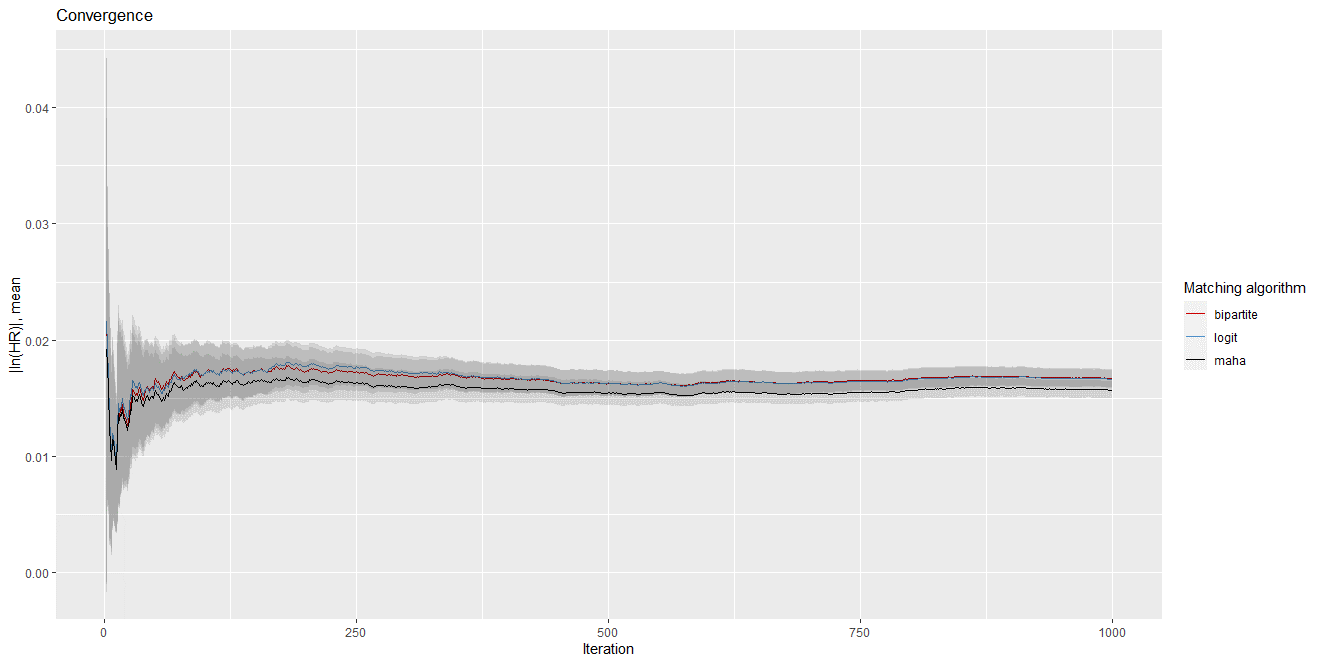 |
| Histogram of ln(HR)  ORIENT-16, PFS, CHEMO, CPS<5  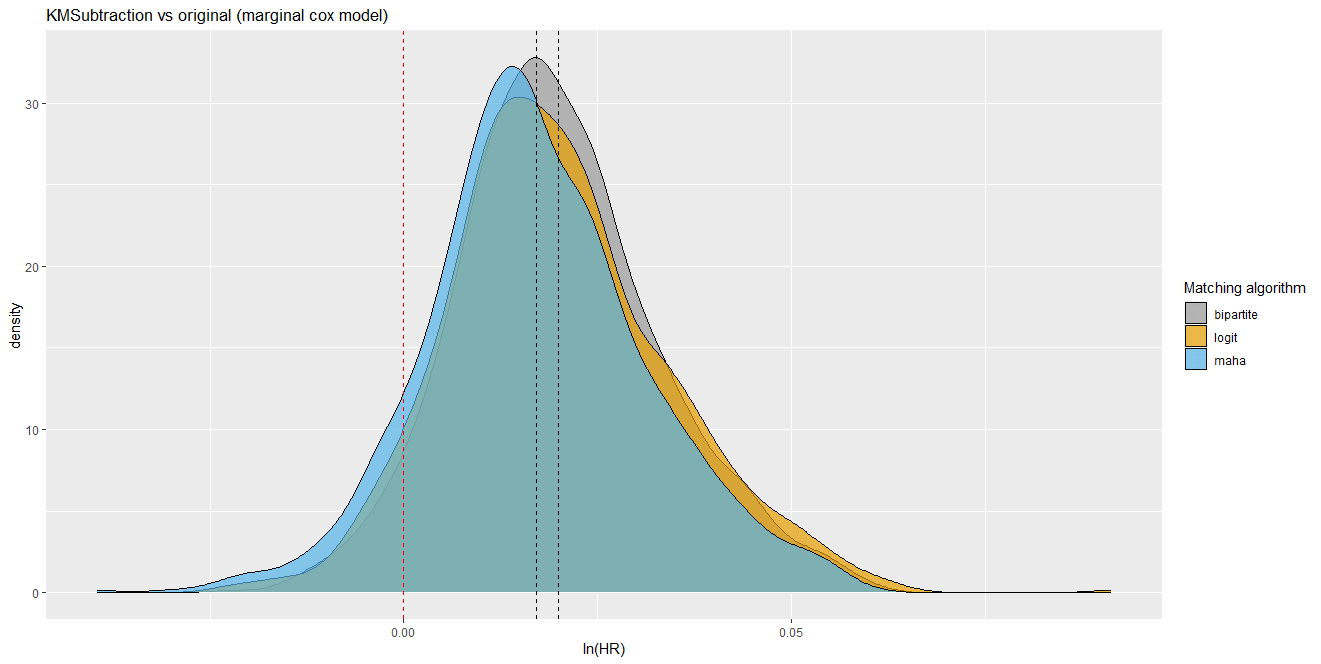 | | Histogram of \|ln(HR)\|  ORIENT-16, PFS, CHEMO, CPS<5  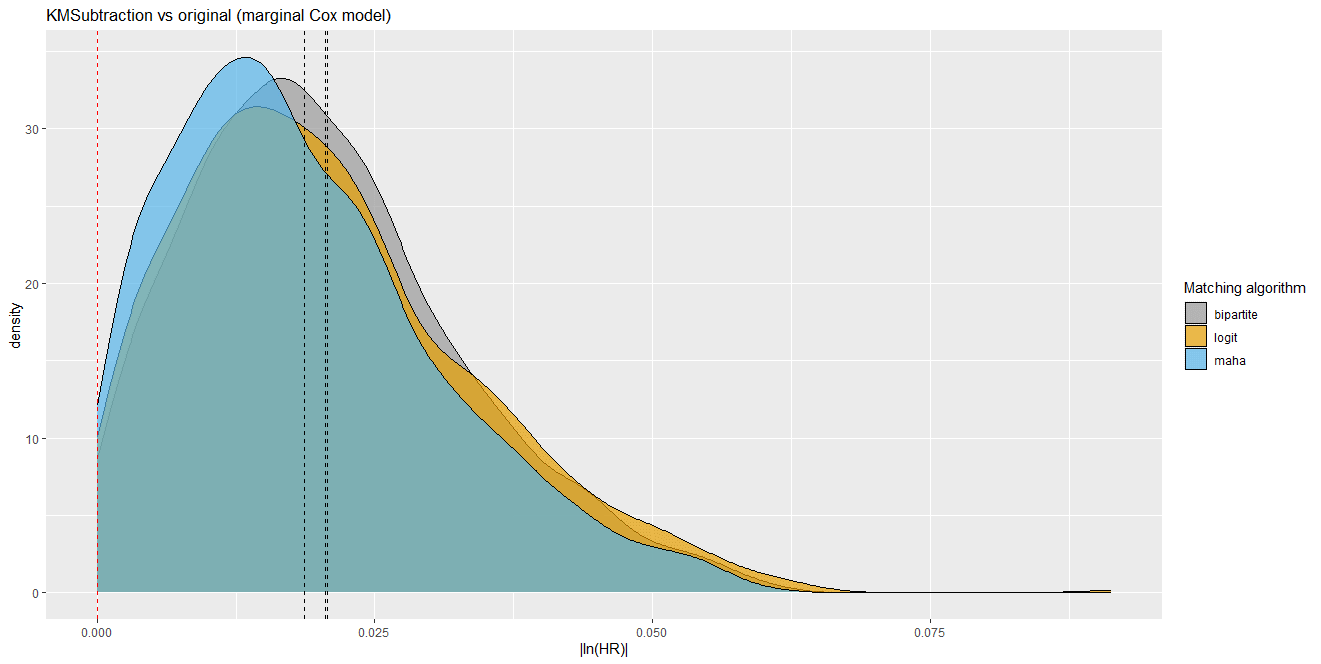 | Convergence plot  ORIENT-16, PFS, CHEMO, CPS<5  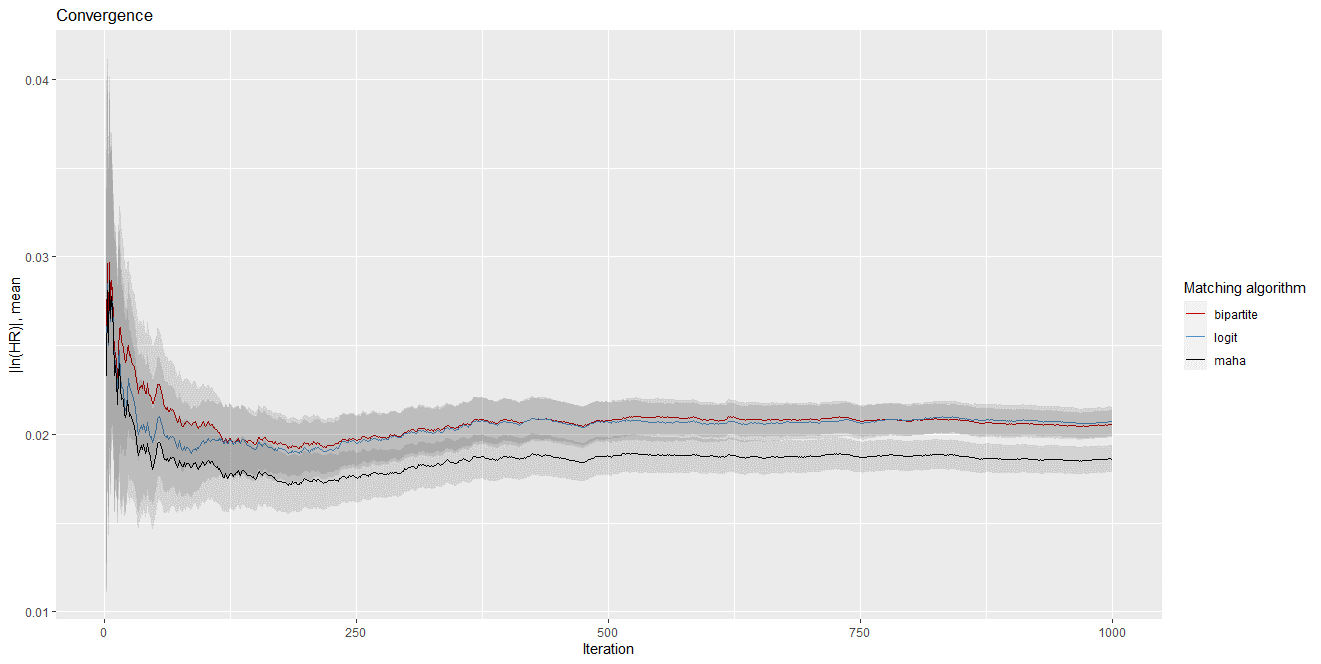 |
| Histogram of ln(HR)  KN-062 global patients, OS, ICI+CHEMO, CPS<10  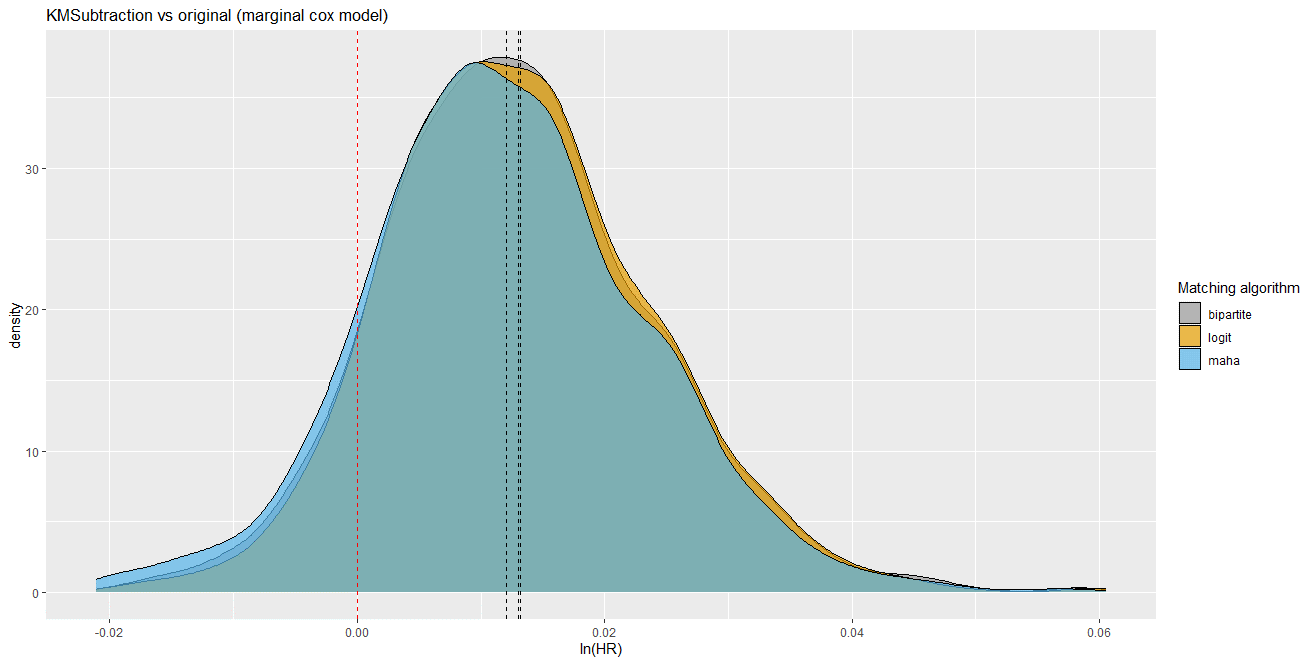 | | Histogram of \|ln(HR)\|  KN-062 global patients, OS, ICI+CHEMO, CPS<10  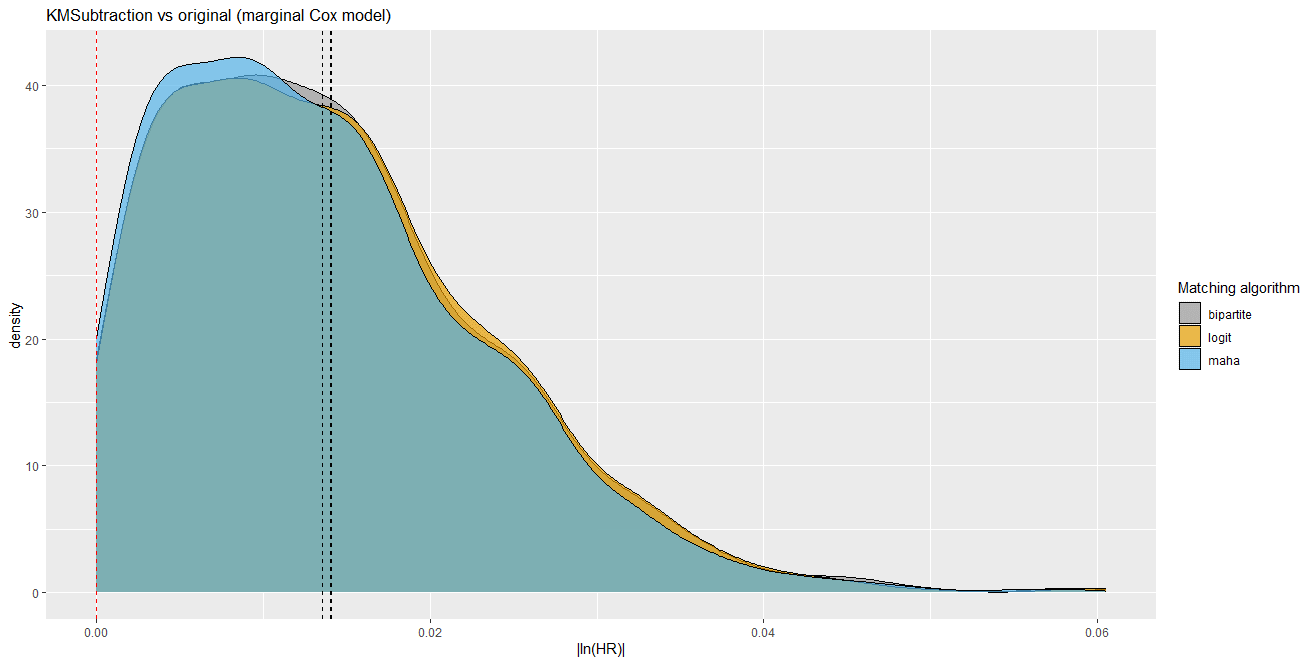 | Convergence plot  KN-062 global patients, OS, ICI+CHEMO, CPS<10  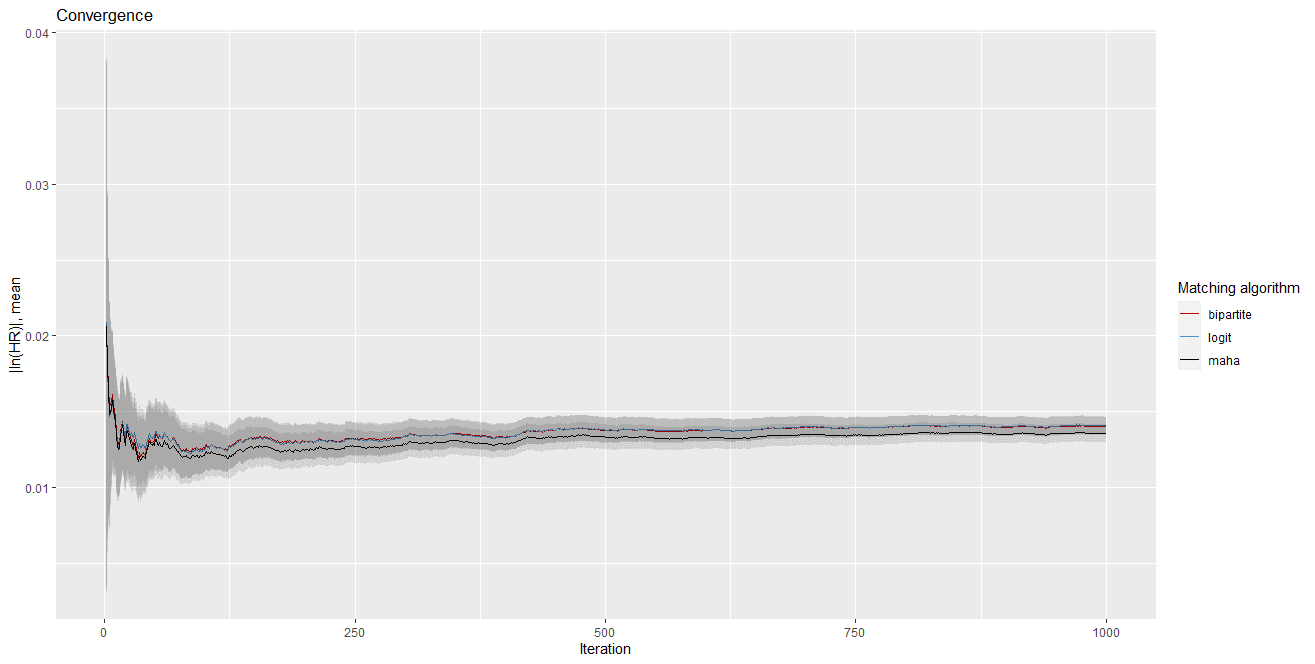 |
| Histogram of ln(HR)  KN-062 global patients, OS, CHEMO, CPS<10  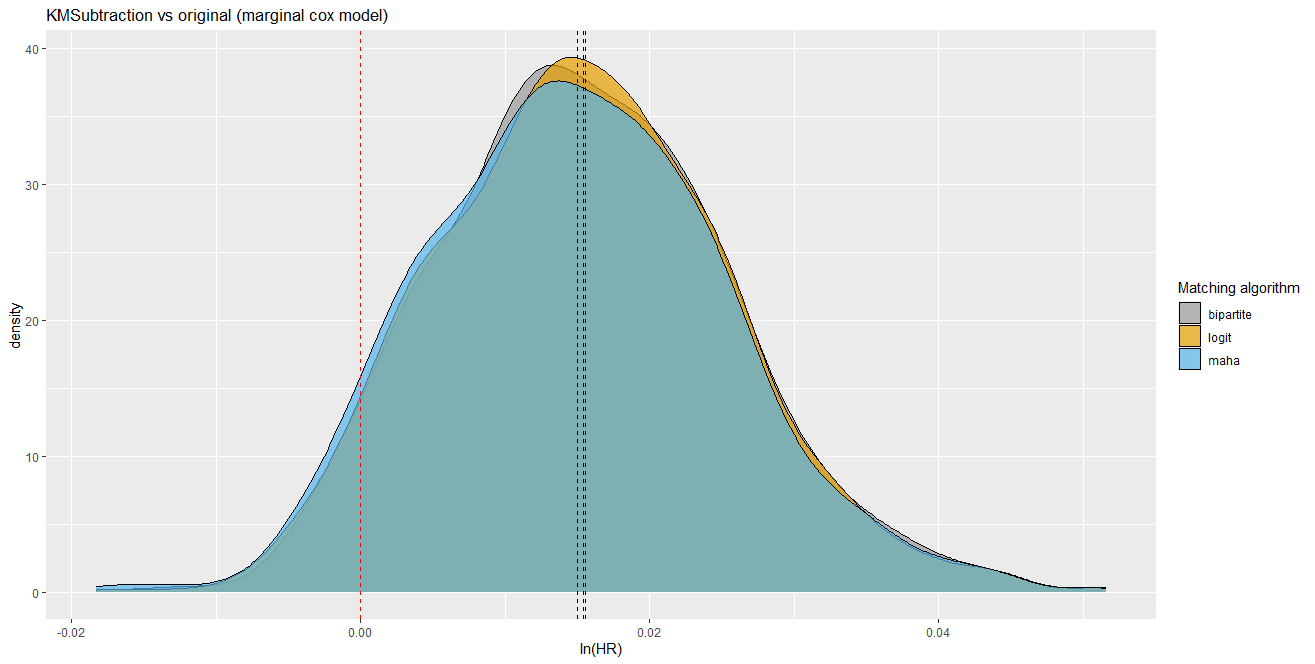 | | Histogram of \|ln(HR)\|  KN-062 global patients, OS, CHEMO, CPS<10  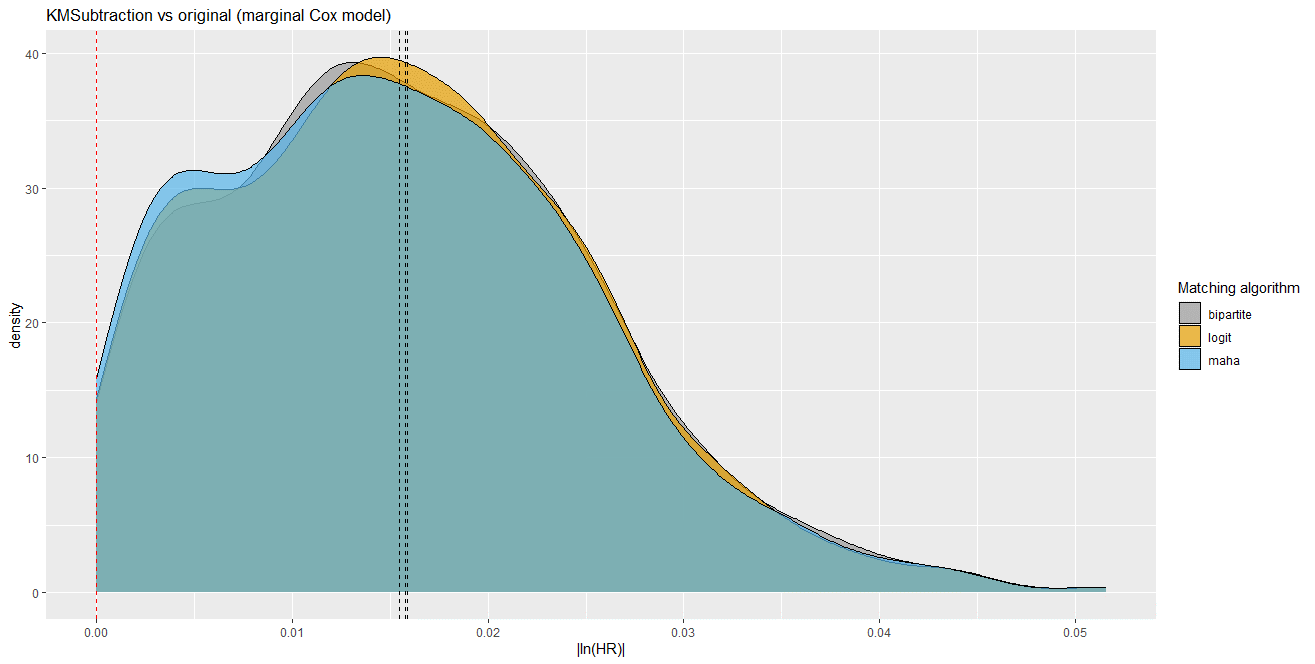 | Convergence plot  KN-062 global patients, OS, CHEMO, CPS<10  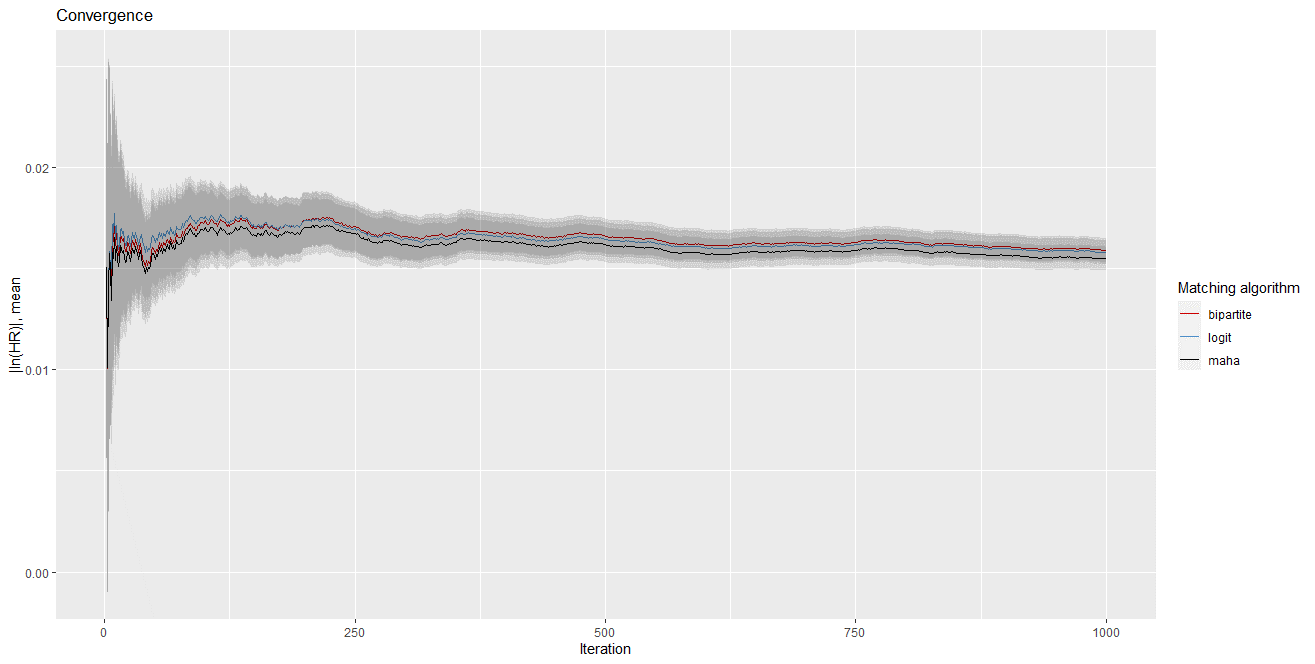 |
| Histogram of ln(HR)  KN-062 global patients, PFS, ICI+CHEMO, CPS<10 | | Histogram of \|ln(HR)\|  KN-062 global patients, PFS, ICI+CHEMO, CPS<10 | Convergence plot  KN-062 global patients, PFS, ICI+CHEMO, CPS<10 |
| Histogram of ln(HR)  KN-062 global patients, PFS, CHEMO, CPS<10 | | Histogram of \|ln(HR)\|  KN-062 global patients, PFS, CHEMO, CPS<10 | Convergence plot  KN-062 global patients, PFS, CHEMO, CPS<10 |
| Histogram of ln(HR)  KN-062 Asian patients, OS, ICI+CHEMO, CPS<10 | | Histogram of \|ln(HR)\|  KN-062 Asian patients, OS, ICI+CHEMO, CPS<10 | Convergence plot  KN-062 Asian patients, OS, ICI+CHEMO, CPS<10 |
| Histogram of ln(HR)  KN-062 Asian patients, OS, CHEMO, CPS<10 | | Histogram of \|ln(HR)\|  KN-062 Asian patients, OS, CHEMO, CPS<10 | Convergence plot  KN-062 Asian patients, OS, CHEMO, CPS<10 |
| Histogram of ln(HR)  KN-062 Asian patients, PFS, ICI+CHEMO, CPS<10 | | Histogram of \|ln(HR)\|  KN-062 Asian patients, PFS, ICI+CHEMO, CPS<10 | Convergence plot  KN-062 Asian patients, PFS, ICI+CHEMO, CPS<10 |
| Histogram of ln(HR)  KN-062 Asian patients, PFS, CHEMO, CPS<10 | | Histogram of \|ln(HR)\|  KN-062 Asian patients, PFS, CHEMO, CPS<10 | Convergence plot  KN-062 Asian patients, PFS, CHEMO, CPS<10 |
| Histogram of ln(HR)  KN-590, OS, ICI+CHEMO, all patients | | Histogram of \|ln(HR)\|  KN-590 OS, ICI+CHEMO, all patients | Convergence plot  KN-062, OS, ICI+CHEMO, all patients |
| Histogram of ln(HR)  KN-590, OS, CHEMO, all patients | | Histogram of \|ln(HR)\|  KN-590, OS, CHEMO, all patients | Convergence plot  KN-590, OS, CHEMO, all patients |
| Histogram of ln(HR)  KN-590, OS, ICI+CHEMO, CPS≥10 | | Histogram of \|ln(HR)\|  KN-590, OS, ICI+CHEMO, CPS≥10 | Convergence plot  KN-062, OS, ICI+CHEMO, CPS≥10 |
| Histogram of ln(HR)  KN-590, OS, CHEMO, CPS≥10 | | Histogram of \|ln(HR)\|  KN-590, OS, CHEMO, CPS≥10 | Convergence plot  KN-590, OS, CHEMO, CPS≥10 |
| Histogram of ln(HR)  KN-590, OS, ICI+CHEMO, CPS<10 | | Histogram of \|ln(HR)\|  KN-590, OS, ICI+CHEMO, CPS<10 | Convergence plot  KN-062, OS, ICI+CHEMO, CPS<10 |
| Histogram of ln(HR)  KN-590, OS, CHEMO, CPS<10 | | Histogram of \|ln(HR)\|  KN-590, OS, CHEMO, CPS<10 | Convergence plot  KN-590, OS, CHEMO, CPS<10 |
| Histogram of ln(HR)  KN-590, PFS, ICI+CHEMO, all patients | | Histogram of \|ln(HR)\|  KN-590, PFS, ICI+CHEMO, all patients | Convergence plot  KN-062, PFS, ICI+CHEMO, all patients |
| Histogram of ln(HR)  KN-590, PFS, CHEMO, all patients | | Histogram of \|ln(HR)\|  KN-590, PFS, CHEMO, all patients | Convergence plot  KN-590, PFS, CHEMO, all patients |

# Table S9. Sensitivity analysis for the efficacy of ICI plus chemotherapy compared to chemotherapy using the Bayesian random-effect method

| Variables |  | No. of studies | *I*^2^ (%) | Pooled HR (95% CI) |
| --- | --- | --- | --- | --- |
| **OS** |  |  |  |  |
| Global patients | Half-Norm (0.5) | 6 | 9.4 | 0.79 (0.71,0.88) |
|  | Half-Norm (1.0) |  |  | 0.79 (0.71,0.88) |
| Asian patients | Half-Norm (0.5) | 4 | 54.3 | 0.75 (0.55,1.00) |
|  | Half-Norm (1.0) |  |  | 0.75 (0.52,1.05) |
| **OS in global patients** |  |  |  |  |
| CPS≥1 | Half-Norm (0.5) | 4 | 24.1 | 0.74 (0.62,0.89) |
|  | Half-Norm (1.0) |  |  | 0.74 (0.61,0.91) |
| CPS<1 | Half-Norm (0.5) | 3 | 0 | 0.89 (0.63,1.24) |
|  | Half-Norm (1.0) |  |  | 0.89 (0.56,1.38) |
| CPS≥5 | Half-Norm (0.5) | 2 | 34.1 | 0.65 (0.37,1.12) |
|  | Half-Norm (1.0) |  |  | 0.65 (0.25,1.68) |
| CPS<5 | Half-Norm (0.5) | 2 | 0 | 0.89 (0.51,1.52) |
|  | Half-Norm (1.0) |  |  | 0.89 (0.34,2.25) |
| CPS≥10 | Half-Norm (0.5) | 4 | 31.7 | 0.69 (0.51,0.95) |
|  | Half-Norm (1.0) |  |  | 0.69 (0.48,1.02) |
| CPS<10 | Half-Norm (0.5) | 3 | 0 | 0.83 (0.57,1.12) |
|  | Half-Norm (1.0) |  |  | 0.82 (0.50,1.26) |
| **OS in Asian patients** |  |  |  |  |
| CPS≥1 | Half-Norm (0.5) | 3 | 0 | 0.67 (0.48,0.96) |
|  | Half-Norm (1.0) |  |  | 0.67 (0.42,1.09) |
| CPS<1 | Half-Norm (0.5) | 2 | 0 | 0.76 (0.36,1.57) |
|  | Half-Norm (1.0) |  |  | 0.75 (0.23,2.38) |
| CPS≥5 | Half-Norm (0.5) | 2 | 0 | 0.57 (0.32,1.00) |
|  | Half-Norm (1.0) |  |  | 0.57 (0.22,1.48) |
| CPS<5 | Half-Norm (0.5) | 2 | 0 | 0.82 (0.44,1.53) |
|  | Half-Norm (1.0) |  |  | 0.82 (0.29,2.30) |
| CPS≥10 | Half-Norm (0.5) | 2 | 26.8 | 0.64 (0.33,1.37) |
|  | Half-Norm (1.0) |  |  | 0.65 (0.21,2.22) |
| **PFS** |  |  |  |  |
| Global Patients | Half-Norm (0.5) | 6 | 16.8 | 0.74 (0.65,0.83) |
|  | Half-Norm (1.0) |  |  | 0.74 (0.64,0.83) |
| Asian patients | Half-Norm (0.5) | 4 | 0 | 0.63 (0.51,0.79) |
|  | Half-Norm (1.0) |  |  | 0.63 (0.49,0.80) |
| **PFS in global patients** |  |  |  |  |
| CPS≥1 | Half-Norm (0.5) | 3 | 0 | 0.76 (0.59,0.99) |
|  | Half-Norm (1.0) |  |  | 0.76 (0.54,1.07) |
| CPS<1 | Half-Norm (0.5) | 2 | 0 | 0.91 (0.53,1.57) |
|  | Half-Norm (1.0) |  |  | 0.91 (0.36; 2.31) |
| CPS≥5 | Half-Norm (0.5) | 2 | 0 | 0.67 (0.39,1.11) |
|  | Half-Norm (1.0) |  |  | 0.67 (0.27,1.63) |
| CPS<5 | Half-Norm (0.5) | 2 | 85.0 | 0.76 (0.36,1.55) |
|  | Half-Norm (1.0) |  |  | 0.76 (0.22,2.51) |
| CPS≥10 | Half-Norm (0.5) | 3 | 0 | 0.63 (0.41,0.92) |
|  | Half-Norm (1.0) |  |  | 0.63 (0.35,1.06) |
| CPS<10 | Half-Norm (0.5) | 3 | 0 | 0.87 (0.62,1.19) |
|  | Half-Norm (1.0) |  |  | 0.87 (0.55,1.33) |
| **PFS in Asian patients** |  |  |  |  |
| CPS≥1 | Half-Norm (0.5) | 2 | 0 | 0.59 (0.33,1.07) |
|  | Half-Norm (1.0) |  |  | 0.59 (0.22,1.60) |
| CPS≥5 | Half-Norm (0.5) | 2 | 0 | 0.59 (0.32,1.05) |
|  | Half-Norm (1.0) |  |  | 0.59 (0.21,1.58) |
| CPS<5 | Half-Norm (0.5) | 2 | 0 | 0.62 (0.33,1.22) |
|  | Half-Norm (1.0) |  |  | 0.63 (0.22,1.88) |

OS: Overall survival; PFS: Progress-free survival; HR: Hazard ratio; 95%CI: 95% confidence interval.

# Table S10. Sensitivity analysis for the efficacy of ICI plus chemotherapy compared to chemotherapy after excluding ORIENT-16 and ATT-04 from the global analysis

| Variables | *n* of study | *I*^2^ | Pooled HR [95%CI] | Predictive value (%) | Ratio (95% CI) |
| --- | --- | --- | --- | --- | --- |
| OS |  |  |  |  |  |
| Global | 4 | 0 | 0.79 [0.74; 0.85] | -0.051 | 1.05 (0.80,1.39) |
| Asian | 4 | 54.3 | 0.75 [0.57; 0.98] |  |  |
| OS in Global |  |  |  |  |  |
| CPS≥1 | 3 | 0 | 0.76 [0.70; 0.83] | 0.211 | 0.83 (0.68,1.01) |
| CPS <1 | 2 | 0 | 0.92 [0.77; 1.10] |  |  |
| CPS≥5 | 1 | 0 | 0.70 [0.60; 0.81] | **0.343** | **0.74 (0.59,0.95)** |
| CPS <5 | 1 | 0 | 0.94 [0.78; 1.13] |  |  |
| CPS≥10 | 3 | 26.5 | 0.73 [0.48; 1.11] | 0.151 | 0.87 (0.56,1.34) |
| CPS <10 | 3 | 0 | 0.84 [0.75; 0.94] |  |  |
| OS in Asian |  |  |  |  |  |
| CPS≥1 | 3 | 0 | 0.66 [0.57; 0.77] | 0.152 | 0.87 (0.57,1.32) |
| CPS <1 | 2 | 0 | 0.76 [0.52; 1.13] |  |  |
| CPS≥5 | 2 | 0 | 0.58 [0.47; 0.70] | **0.414** | **0.71 (0.51,0.97)** |
| CPS <5 | 2 | 0 | 0.82 [0.64; 1.05] |  |  |
| CPS≥10 | 2 | 26.8 | 0.63 [0.06; 7.18] | 0.175 | 0.85 (0.07,9.8) |
| CPS <10 | 1 | 0 | 0.74 [0.45; 1.21] |  |  |
| PFS |  |  |  |  |  |
| Global | 4 | 0 | 0.77 [0.71; 0.83] | -0.169 | 1.20 (1.03,1.40) |
| Asian | 4 | 0 | 0.64 [0.56; 0.73] |  |  |
| PFS in Global |  |  |  |  |  |
| CPS≥1 | 3 | 0 | 0.75 [0.69; 0.81] | 0.213 | 0.82 (0.67,1.01) |
| CPS <1 | 2 | 0 | 0.91 [0.75; 1.10] |  |  |
| CPS≥5 | 1 | 0 | 0.69 [0.59; 0.80] | 0.348 | 0.74 (0.58,0.95) |
| CPS <5 | 1 | 0 | 0.93 [0.77; 1.13] |  |  |
| CPS≥10 | 3 | 0 | 0.63 [0.54; 0.74] | **0.381** | **0.72 (0.6,0.88)** |
| CPS <10 | 3 | 0 | 0.87 [0.77; 0.97] |  |  |
| PFS in Asian |  |  |  |  |  |
| CPS≥1 | 2 | 0 | 0.59 [0.46; 0.77] | -0.136 | 1.16 (0.4,3.39) |
| CPS <1 | 1 | 0 | 0.51 [0.18; 1.45] |  |  |
| CPS≥5 | 2 | 0 | 0.60 [0.48; 0.74] | 0.017 | 0.98 (0.79,1.22) |
| CPS <5 | 2 | 0 | 0.61 [0.48; 0.79] |  |  |
| CPS≥10 | 1 | 0 | 0.46 [0.24; 0.90] | **0.804** | 0.55 (0.27,1.12) |
| CPS <10 | 1 | 0 | 0.83 [0.51; 1.36] |  |  |

OS: Overall survival; PFS: Progress-free survival; HR: Hazard ratio; 95%CI: 95% confidence interval.

# **Figure S1. PRISMA flowchart of study inclusions and exclusions**.

CPS, combined positive score; OS, overall survival; PFS, progression-free survival.

# Figure S2. Overall survival curves of ICI plus chemotherapy versus chemotherapy groups based on the reconstructed IPD data

# **Figure S3. Progress-free survival curves of ICI plus chemotherapy versus chemotherapy groups based on the** reconstructed IPD data.

# Figure S4. Funnel plots for OS and PFS.
